# Pyrazolo-fused 4-azafluorenones as key reagents for the synthesis of fluorescent dicyanovinylidene-substituted derivatives†

**DOI:** 10.1039/c9ra04682h

**Published:** 2019-08-30

**Authors:** Jessica Orrego-Hernández, Carolina Lizarazo, Justo Cobo, Jaime Portilla

**Affiliations:** Bioorganic Compounds Research Group, Department of Chemistry, Universidad de los Andes Carrera 1 No. 18A-10 Bogotá 111711 Colombia jportill@uniandes.edu.co; Departamento de Química Orgánica e Inorgánica, Universidad de Jaén 23071 Jaén Spain

## Abstract

A green process to access pyrazolo-fused 4-azafluorenones (indeno[1,2-*b*]pyrazolo[4,3-*e*]pyridines, IPP) 4a–x*via* the three-component reaction between indan-1,3-dione (1), benzaldehydes 2 and 5-amino-1-arylpyrazoles 3 is described. These compounds were successfully used as precursors of the novel dicyanovinylidene derivatives 7a–d containing different acceptor (A) or donor (D) aryl groups at position 4 of its fused system. The structures of products obtained (4a–x and 7a–d) were determined based on NMR experiments, HRMS analysis, and X-ray diffraction studies for 7b. The photophysical and computational studies of 7a–d showed that these products are modulable ICT fluorophores, even some preliminary tests revealed that these compounds could be used as fluorescent chemodosimeters for cyanide detection.

## Abstract 2

A green method for the three-component synthesis of an indeno[1,2-*b*]pyrazolo[4,3-*e*]pyridines library under microwave irradiation and their use in the preparation of novel fluorescent dicyanovinylidene-substituted derivatives is provided.

## Graphical abstract

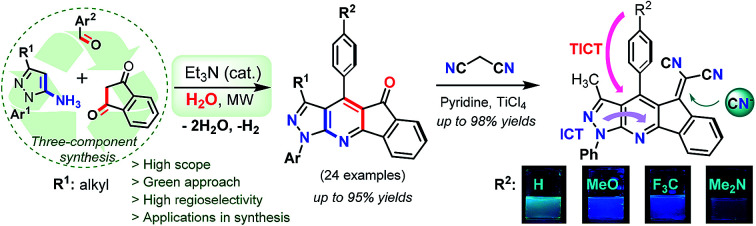

## 1. Introduction

**Fig. 1:**
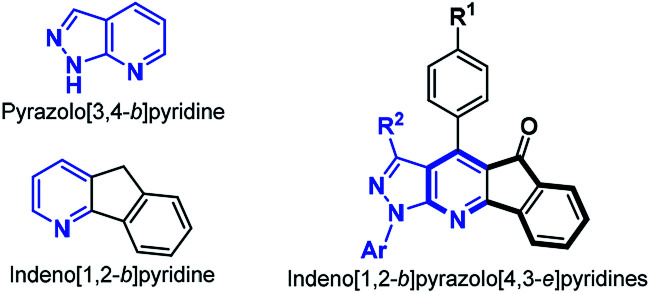
Pyrazolo[3,4-*b*]pyridine, indeno[1,2-*e*]pyridine and their combined-fused derivatives. The 4-azafluorene moiety is remarked in a bold line.

In recent years, the development of a highly efficient atom and step economic synthesis of fused aza-heterocycles to yield biologically active compounds has been actively pursued, and thus has become an important area of research in organic and medicinal chemistry.^1–3^ In particular, pyrazolo[3,4-*b*]pyridines (PP) are of biomedical importance and have been extensively studied for their broad biological activity.^4–6^ Likewise, indeno[1,2-*b*]pyridines (4-azafluorenones, IP) have shown potential as anticancer, antioxidant, antihistamine and antidepressant agents.^7,8^ Both 4-azafluorenones and pyrazolo[3,4-*b*]pyridines have found applications in materials science due to their amazing photophysical properties.^9,10^ Thus, the development of efficient methods for the synthesis and functionalization of fused systems of these two structural moieties (*i.e.*, pyrazolo-fused 4-azafluorenones) is highly desirable (Fig. 1).

**Scheme 1:**
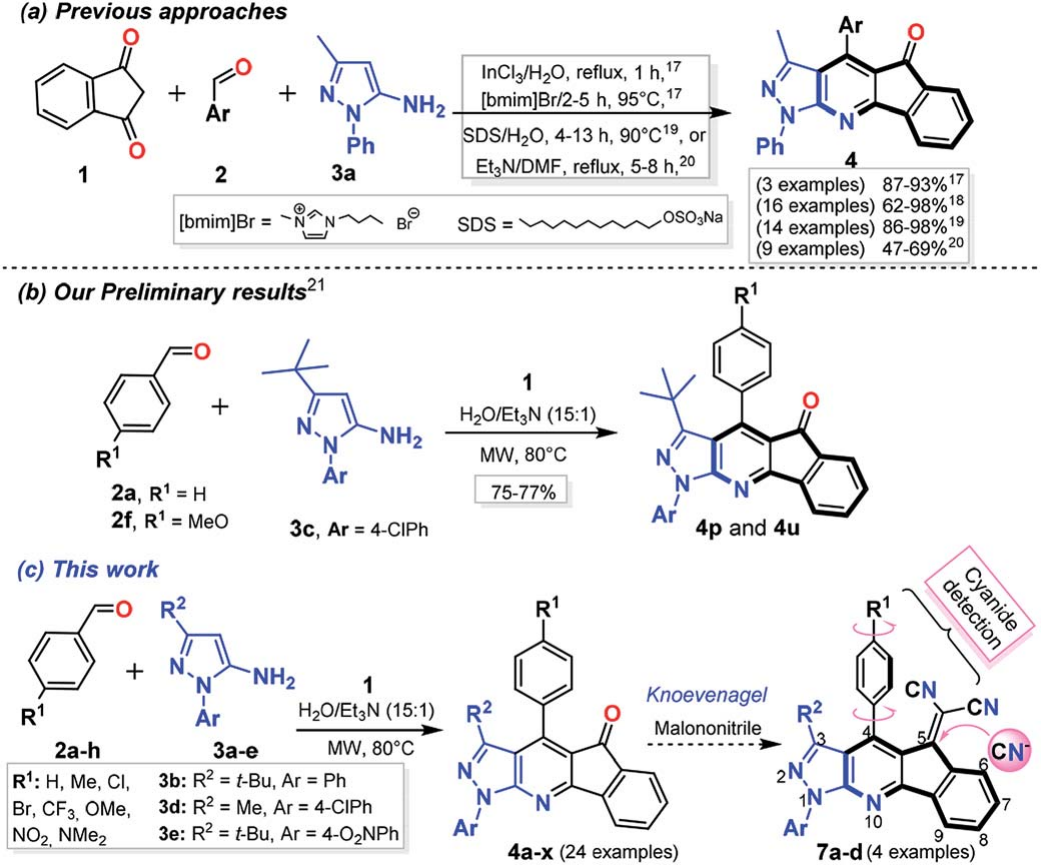
Synthesis of indeno[1,2-*b*]pyrazolo[4,3-*e*]pyridin-5-ones 4 and 4a–x.

Numerous approaches for the synthesis of PP^11,12^ and their fused derivatives have been reported,^13,14^ those reactions involved the interaction of 1,3-bis-electrophilic compounds with *N*-substituted 5-aminopyrazoles.^11–14^ Moreover, multi-component reactions (MCRs) are used in the synthesis of these compounds by the formation *in situ* of the bis-electrophilic intermediate. This approach has been widely used in diversity-oriented synthesis (DOS) of biologically active heterocyclic compounds.^15,16^ However, there are few reports addressing the preparation of indeno-fused PP, and those synthetic procedures have some limitations (*e.g.*, moderate yields, the use of additives or catalysts, ionic liquids as solvent, and reduced substrate scope). Indeed, the most remarkable limitation is the substrate scope, since reactions have been restricted to the use of 5-amino-1-phenyl-3-methylpyrazole (3a) with only a few reported exceptions (Scheme 1a).^17–20^ One of the few examples using a different amine was done from our group, we reported an initial study of the crystal structure of indeno[1,2-*b*]pyrazolo[4,3-*e*]pyridines (IPP) 4p and 4u which was prepared by the three-component reaction between indan-1,3-dione (1), benzaldehydes 2a or 2f, and 5-amino-3-*tert*-butyl-1-(4-chlorophenyl)pyrazole (3c) in good yields. Those reactions were achieved at 80 °C under microwave irradiation (MW) using triethylamine (Et_3_N) as a catalyst in water (Scheme 1b).^21^

Continuing with the development of synthetic methods to obtain pyrazole-fused aza-heterocycles^22–25^ along with our interest to improve the scope of our preliminary results by using different starting 5-aminopyrazoles, we report an extension of the MW-assisted synthesis of indeno[1,2-*b*]pyrazolo[4,3-*e*]pyridines 4a–x*via* the respective multicomponent reaction using the 5-amino-1-arylpyrazoles 3a–e of diverse reactivity. It is important to note that the electron-withdrawing aryl groups at position 1 of the starting amines (*i.e.*, 3c–e) decrease its reactivity towards cyclocondensation reactions,^22^ which would complicate the synthesis of some IPP 4a–x (Scheme 1c). In addition, IPP derivatives have a structural analogy with the fluorene core (Fig. 1), whose derivatives have important fluorescent properties with applications on the design of OLEDs and organic transistors.^26–29^ Due to the structural features of ketones 4a–x, the post-functionalization reactions and the physicochemical study of their respective functional products are of great interest. The synthesis of the dicyanovinylidene derivatives 7a–d and their photophysical properties with an initial application in fluorescent probes were also included, which has been an area that has been recently studied in our group (Scheme 1c).^22,30–33^

## 2. Results and discussion

### 2.1. Synthesis

Starting from our preliminary and promising results of the synthesis of IPP 4p and 4u,^21^ we have studied a range of reaction using different starting 5-aminopyrazoles 3a–e and benzaldehydes 2a–h (Scheme 1c). Our study of these polycyclic compounds synthesis began by optimization of the reaction conditions (conventional or MW heating, solvent, base, temperature, and reaction time) by using as a model reaction an equimolar mixture of indan-1,3-dione (1), benzaldehyde (2a) and 5-amino-1-phenyl-3-methyl-1*H*-pyrazol (3a). As expected, the reaction gave the desired IPP 4a in good yield under the same conditions reported in our previous work (in H_2_O : Et_3_N at 80 °C for 10 min under MW^21^), and yield was improved by prolonging the reaction time, but not the temperature (Table 1, entries 1–3). Heating under reflux in different solvents using triethylamine as a catalyst offered the product 4a but in low to moderate yields (Table 1, entries 4–6). We found out that higher temperatures favors formations of the IPP 4a,^20^ while lower temperatures result in low yield (Table 1, entry 5 *vs.* 6).^34^

**Table 1:** Reaction condition optimization for the synthesis of 4a^a^

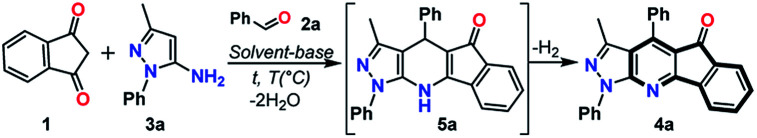				
Entry	Solvent : base	*T* (°C)	Time *t*	Yield [%]
1	H_2_O : Et_3_N	80^b^	10 min	68
2	H_2_O : Et_3_N	80^b^	15 min	84
3	H_2_O : Et_3_N	100^b^	15 min	83
4	H_2_O : Et_3_N	Reflux^c^	15 min	44
5	DMF : Et_3_N	Reflux^c^	24 h	60
6	EtOH : Et_3_N	Reflux^c^	24 h	25
7^d^	H_2_O	80^b^	24 h	—
8	EtOH	80^b^	15 min	43
9	H_2_O : KOH	80^b^	15 min	22
10	H_2_O : K_2_CO_3_	80^d^	15 min	20

a Reaction conditions: equimolar quantities (0.25 mmol) of 1, 2a and 3a.

b Run in 10 mL sealed tube under MW in 0.7 mL of solvent (0.1 equiv. of base) or a mixture H_2_O : Et_3_N (15 : 1 v/v).

c Conventional heating in a solvent : Et_3_N mixture (2 mL, 15 : 1 v/v).

d A dihydropyridine intermediate was isolated, see ESI.

When the reaction was carried out without base or other bases (KOH or K_2_CO_3_) in water or ethanol under MW, the formation of 4a was diminished (Table 1, entries 7–10). Consequently, the optimal conditions were set to obtain 4a in a similar way to those reported in our preliminary study (Table 1, entry 2).^21^ In general, low yields for the formation of 4a were observed when ethanol was used as a solvent (Table 1, entries 6 and 8). These conditions are closely related to those used for pyrazolo[5,1-*b*]quinazolines synthesis reported by Chebanov *et al.* (by using dimedone, arylaldehydes and a *NH*-pyrazole).^35^ On the other hand, when the reaction was carried out in only water (Table 1, entry 7), the dihydropyridine 5a was isolated instead of final product 4a. The structure of this intermediate was deduced by NMR spectroscopy and HPLC-HRMS spectrometry, concluding that the Et_3_N not only promotes the formation of 5a, but also favors its final oxidation to afford 4a (see Fig. S1 and S2, ESI†).

**Scheme 2:**
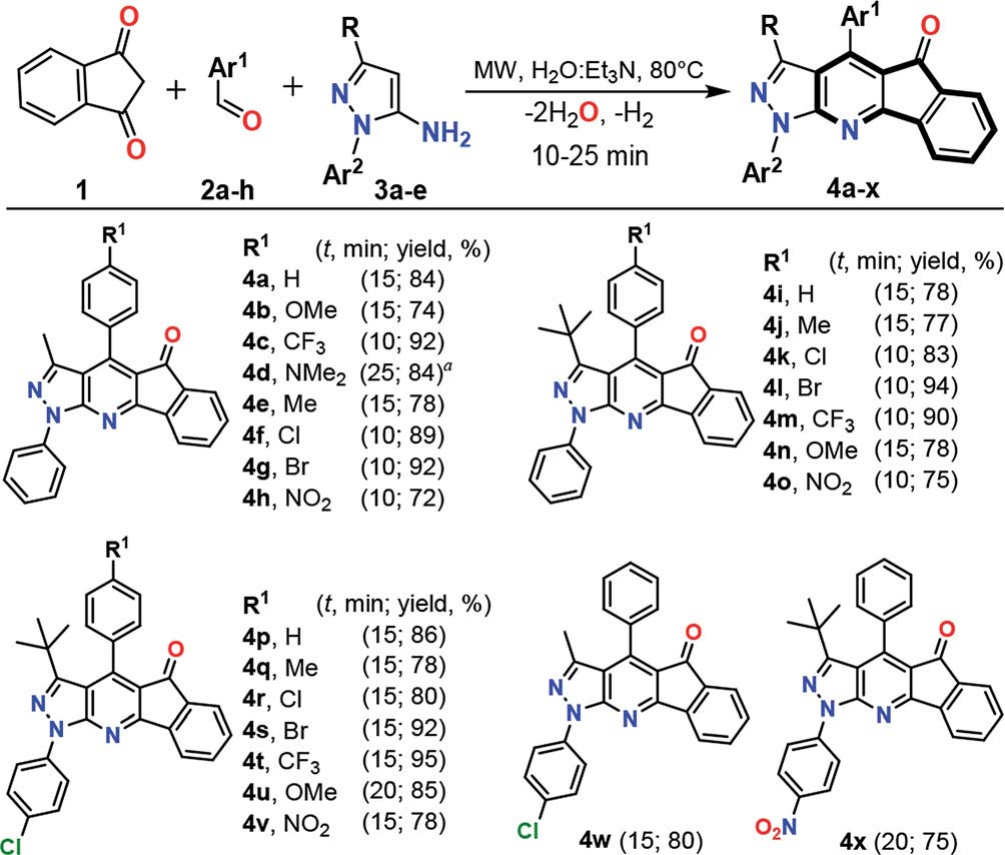
MW-assisted synthesis of pyrazolo-fused 4-azafluorenones 4a–x. Reaction conditions: equimolar quantities (0.25 mmol) of 1, 2a–h and 3a–e in a mixture H_2_O : Et_3_N (0.7 mL, 15 : 1 v/v). Run in 10 mL sealed tube under MW. ^*a*^Intermediate 6h was isolated after 10 min of reaction; see ESI† for details.

Once the optimal conditions to obtain 4a were achieved, we explored a range of benzaldehydes 2a–h and aminopyrazoles 3a–e (prepared and available in our lab^23^) in order to test their reactivities and produce the variously substituted IPP 4a–x. Thus, the reaction under MW conditions for 10–25 min of an equimolar quantity of precursors 1, 2a–h and 3a–e gave the expected products 4a–x in good yields. Almost all reactions showed a low electronic effect of the substituent groups on the precursor's reactivity, but longer reaction times are required when less reactive amines are used (*e.g.*, to form the products 4p–x, where amines 3 have electron-withdrawing groups).^22,23^ Moreover, in the reactions using benzaldehydes having electron-donating groups, longer reaction times are required to give the desired products (Scheme 2). The formation of the fused compounds 4a–x was confirmed by their complete spectral characterization (see Experimental, ESI†).

**Scheme 3:**
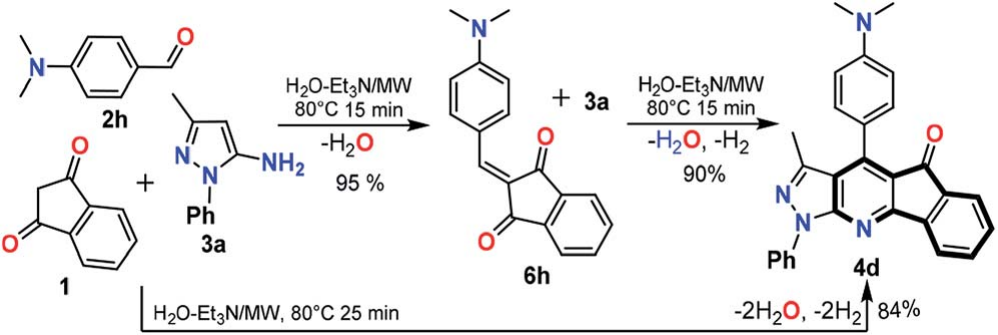
Synthesis of 4d*via* the intermediate 6h; see ESI† for details.

Casually, in the reaction using the poorly electrophilic benzaldehyde 2h under the general conditions by MW (80 °C in H_2_O : Et_3_N for 15 min) a different compound was formed 6h. HRMS and NMR analysis confirmed that this product was obtained by the condensation of 1 with 2h without the participation of the respective amine 3a (see Experimental in ESI†). Subsequent MW reaction of the intermediate 6h with one equiv. of 3a (for 15 min at 80 °C) leads to the desired product 4d in 90% yield, while a longer reaction time (25 min) directly obtain 4d*via* the multicomponent reaction (Scheme 3). These results confirm that the synthesis of the IPP 4a–x proceeds by the intermediate 6h, which then reacts with the amine 3a with subsequent loss of water and hydrogen molecules, in agreement with previous works.^17–20^ Broadly, this methodology was optimized and successfully tested using various substrates allowing its generality and greener approach.

**Scheme 4:**
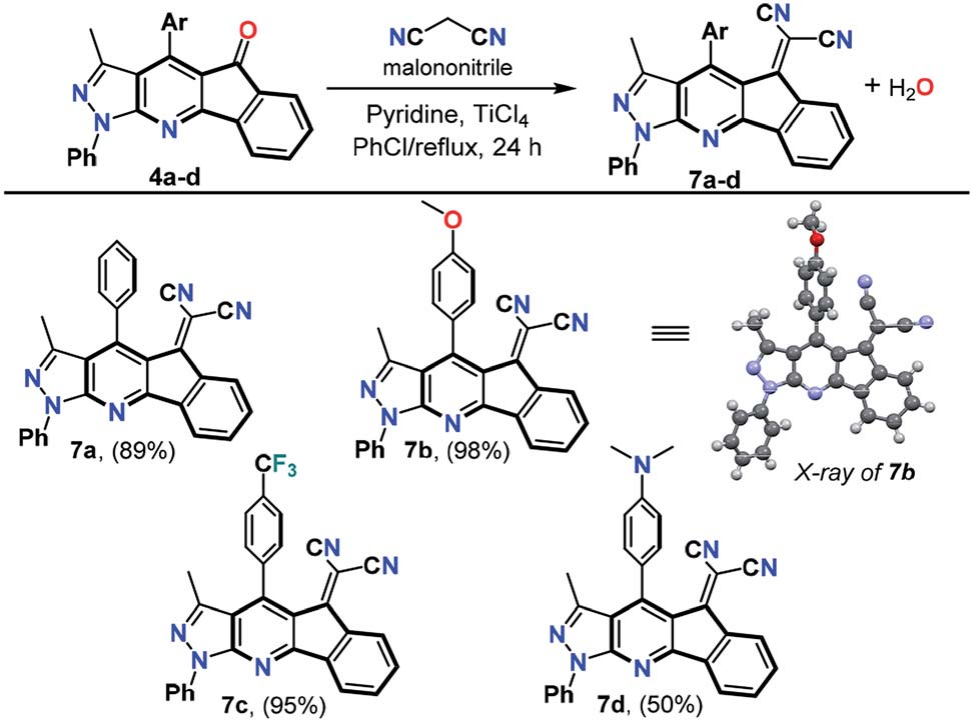
Synthesis of dicyanovinylidene derivatives 7a–d. Reaction conditions: 4a–d (1 equiv.), malononitrile (10 equiv.), pyridine (20 equiv.), and TiCl_4_ (10 equiv.); see ESI† for details.

With the ketones 4a–x in hand, we carried out Knoevenagel reaction with malononitrile to produce the dicyanovinylidene derivatives 7a–d substituted at position 4 with different donor (D) or acceptor (A) aryl groups. Products 7a–d were prepared in good to excellent yields using an excess of malononitrile in the presence of titanium chloride (TiCl_4_) and pyridine in chlorobenzene at reflux for 24 h (Scheme 4). These reaction conditions are analogous to those reported with fluorenones containing a sterically hindered carbonyl group,^36–38^ such as the structures 4a–x. Therefore, special reaction conditions were required to yield compounds 7a–d since the traditional methods did not work.^31,32,39^ These compounds were synthesized considering the important photophysical properties of pyrazole derivatives^22,30–32,40,41^ and the azafluorenone moiety.^42^ In addition, the dicyanovinylidene is widely used as an acceptor moiety in the design of D–π–A dyes that exhibit intramolecular charge transfer (ICT) photophysical process.^31,43^ The structures of the products 7a–d were determined by HRMS analysis, ^1^H spectroscopy, and ^13^C NMR spectroscopy. Recrystallization of the product 7b from *N*,*N*-dimethylformamide (DMF) afforded crystals of suitable size and quality for single-crystal X-ray diffraction analysis (Fig. S12, ESI†).^44^

### 2.2. Photophysical studies

**Fig. 2:**
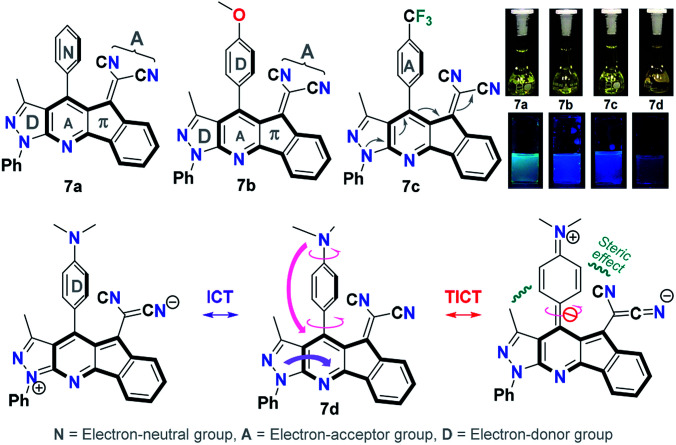
Structure of 7a–d. Photographs were taken using 50 μM solutions in DMSO. A hand-held UV lamp under long wavelength (*λ* = 365 nm) was used.

**Fig. 3:**
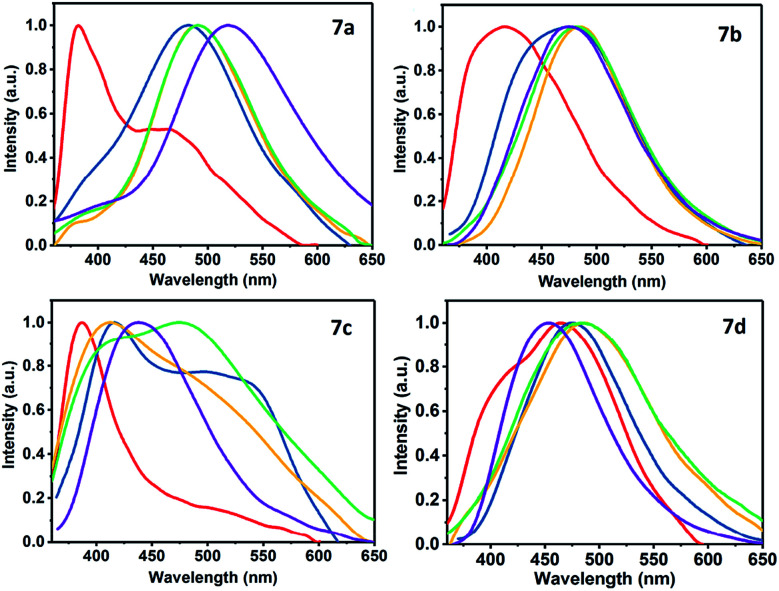
Normalized fluorescence spectra of 7a–d in different solvents. Red (PhMe), blue (DCM), yellow (acetone), green (MeCN), and purple (DMSO).

Solvatochromic studies of dicyanovinylidene derivatives 7a–d with different electron-donor (D) and electron-acceptor (A) groups were carried out in order to establish if these products can be used as new organic fluorophores (Fig. 2). The UV-vis absorption spectra (Fig. S3†) and fluorescence emission (Fig. 3 and S4†) were taken in solvents of different polarity such as toluene (PhMe), dichloromethane (DCM), acetone, acetonitrile (ACN), and dimethylsulfoxide (DMSO) at 50 μM (Table S1†). The UV-vis spectra of 7a–d showed two distinctive absorption bands around 300 and 430 nm, the first can be assigned to transitions π → π* and the second (with the lower intensity) around 430 nm can be attributed to transitions So → intramolecular charge transfer (ICT). This band is caused by an ICT from the pyrrole-type N atom of the pyrazolic ring to a C≡N group, and its intensity reveals a weak ICT effect consistent with its poor solvatochromism in polar solvents (Fig. 2 and S3†).

Regarding the emission spectra of 7a–d, both the quantum yields and fluorescence do not have a significant dependence on the solvent polarity and the 4-aryl group. Products 7b and 7c showed a higher fluorescence emission in DMSO and a hypsochromic shift compared with 7a and 7d. The compound 7d had the higher fluorescence in acetonitrile with respect to 7a–c, showing a bathochromic shift. The compound 7b displayed the highest emission of fluorescence in acetone, whereas in dichloromethane it was the compound 7c. Finally, the use of toluene as a solvent did not show significant fluorescence emission in the heterocycles 7a–d (Fig. S4 and Table S1†). Fig. 3 shows the direct effect of the solvent in each of the products 7a–d, where 7d is the only compound that shows a bathochromic shift as the polarity of the solvent increases. This result indicates that there is a more significant ICT in 7d due to the strong donor character of the dimethylamino group (NMe_2_) that makes the D–π–A system more efficient.

Although 7d exhibited a greater ICT *versus*7a–c, the fluorescence emission in DMSO (*ϕ* = 0.002) is very low compared to acetonitrile (*ϕ* = 0.181). This phenomenon can be the result of a positive solvatokinetic effect that consists in a reduction of the quantum yield due to a high degree of ICT that causes an increase of the speed of non-radiative relaxation of the excited state.^45^ Likewise, a dual emission in the fluorescence spectra was observed mainly in toluene for 7a, 7c and 7d. The first band is assigned to the locally excited state (LE) by ICT processes, while the second to a twisted intramolecular charge transfer (TICT) processes.^46^ These results agree with the structural features of 7a–d, since the TICT phenomena are very sensitive to D → A efficacy and strength, the molecular microenvironment and steric effect between groups near the D–A junction.^22^ The structural relaxation of excited states in weakly polar solvents (reduced interaction with 7a–d in its excited state) allows a greater freedom of rotation in the D–A junctions, thus offering a dual fluorescence (Fig. 2, 3 and S4†).^22^

**Scheme 5:**
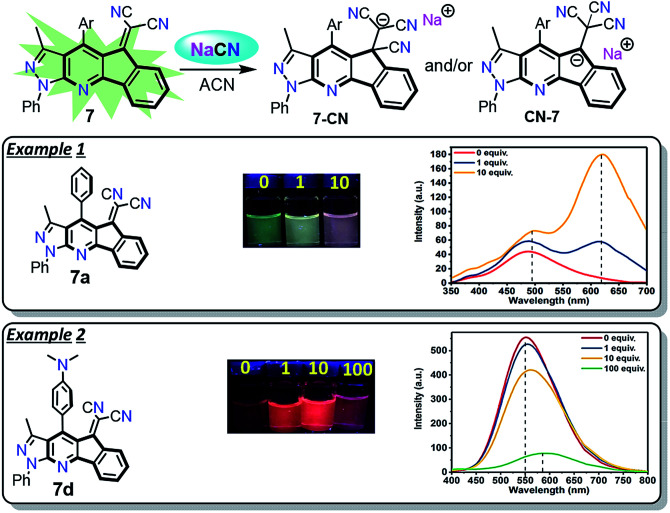
Fluorescent probes for CN^−^ detection. Solutions 50 μM in ACN with CN^−^ (0 to 100 equiv.). Photograph was taken using a hand-held UV lamp (*λ* = 365 nm).

Preliminary UV-vis and fluorescence studies of 7a–d in the presence of different ions were carried out to identify the possible application of these compounds in chemosensors design. From qualitative test of 7a–d in acetonitrile with anions, cyanide ion (CN^−^) caused a significant change in the absorption and fluorescence emission of 7a–d. Besides, the fluorescence emission effect by adding different equivalents of CN^−^ to solutions of 7a–d in acetonitrile was evaluated (Fig. S10 and S11 and Table S3, ESI†). From the UV-vis absorption spectra, a decrease of the band round 430 nm was observed after CN^−^ was added, showing that a nucleophilic addition occurred on the dicyanovinylidene group (Fig. S10†).^32^ The emission spectra of 7a–c showed a new band round 620 nm with an increased fluorescence upon CN^−^ addition (Fig. S11†). The increase in quantum yield is 6 times higher after adding 10 equiv. of CN^−^ (Table S3†). An exception of this trend occurred with 7d, a decrease in the emission around 550 nm with a slight bathochromic shift was observed when CN^−^ was added (Fig. S11†). After adding 100 equiv. of CN^−^, it was observed that the quantum yield was 8 times lower compared with the initial fluorescence (Table S3† and Scheme 5).

The preliminary results in CN^−^ sensing showed that 7d, substituted with the 4-Et_2_NPh group, is the least reactive of the compounds 7a–d. Likewise, the regioisomeric addition products 7-CN or CN-7 could be obtained according to their better stabilization. Possibly, CN-7 is most favored when derivatives 7a–c were used since the emission spectra show a high bathochromic shift, which is characteristic for this type of highly-conjugated heteroaromatic anion (Scheme 5 and Fig. S11†). The study towards the design of TURN-ON and TURN-OFF sensors based on the structures of 7a–d is still ongoing.

### 2.3. Computational calculation

**Fig. 4:**
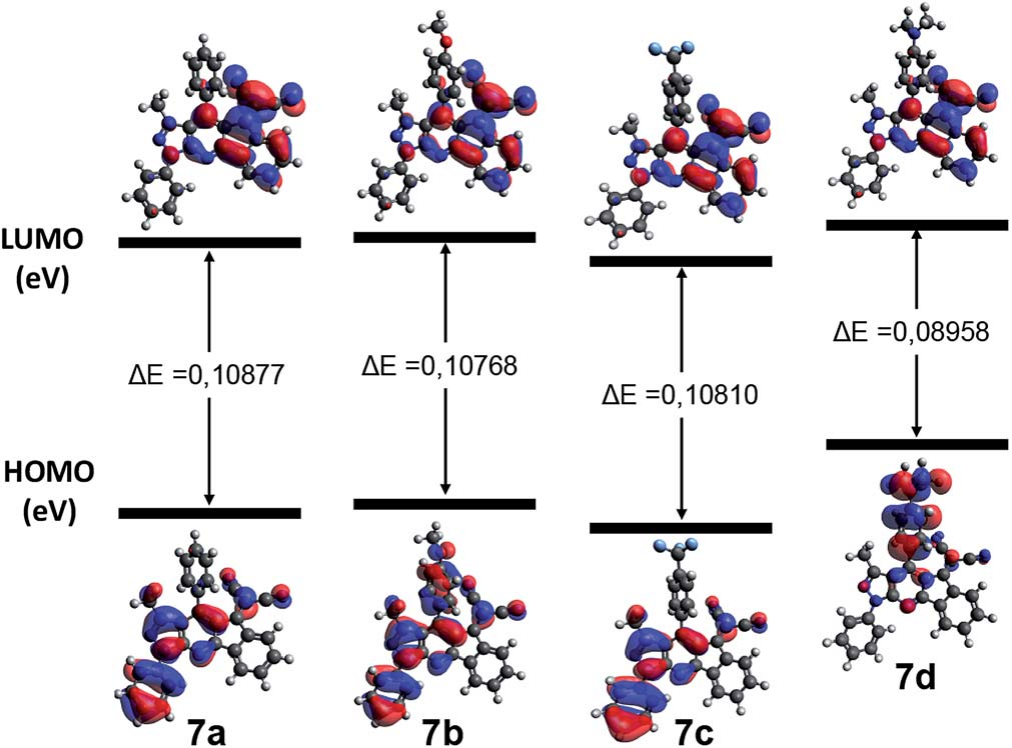
Frontier orbitals of 7a–d generated using Avogadro.^48^

In order to understand the nature of the electronic transitions present in the compounds 7a–d, TD-DFT calculations were done in gas phase. The *λ* max values were obtained from the theoretical UV-vis spectra with a theory level B3LYP/6-31G (d,p). The maximum calculated UV absorptions, the theoretical electronic excitation energies, the calculated oscillator forces and the electronic gas phase transitions are detailed in Table S2.† The band between 340 and 350 nm is assigned mainly to the transition HOMO−5 → LUMO in 7a, HOMO−1 → LUMO in 7b, HOMO−4 → LUMO in 7c, and HOMO−5 → LUMO in 7d, where it is predicted that these transitions are of nature π → π*. According to the diagrams of frontier orbitals of these transitions (Fig. 4 and S5–S9 of ESI†), the electronic nature of the substituent groups of the aryl ring has a significant influence on the ICT towards the dicyanovinylidene group, where 7a showed a charge transfer from the 4-phenyl group and the indene moiety.

Compound 7b is carried out from the *p*-methoxyphenyl group and the phenylpyrazole fragment, while at 7c, this phenomenon occurs from the entire molecule. Finally, for 7d this CT is favored mainly from the *p*-dimethylaminophenyl group to dicyanovinylidene group, due to the strong donor character of this substituent. The energy levels of the frontier orbitals of compounds 7a–d are illustrated in the Fig. 4. These results showed that an electron withdrawing group such as CF_3_ could stabilize the energy levels of both the HOMO and LUMO orbitals, due to its inductive effect. On the other hand, electron-donating groups such as OMe and NMe_2_, destabilize both border orbitals.^47^ Additionally, comparing the band gap energy (Δ*E*) of the compounds 7a–d, it can be concluded that 7d has a lower Δ*E* due to the strong electron-donor character of the dimethylamino group (NMe_2_) that favors a higher CT, which makes it less reactive towards nucleophilic addition reactions.

## 3. Conclusions

In summary, we have developed a MW-assisted method to promote the three-component synthesis of the attractive synthetic scaffold IPP 4a–x using diverse starting *N*-arylpyrazoles and Et_3_N as catalyst. With this green approach using water as a solvent, high yields of all products with short reaction times were obtained. The IPP 4a–x are of great interest as reagents to prepare important derivatives with biological and optical applications. Thus, novel dicyanovinylidene derivatives 7a–d were synthesized from precursors 4a–d and their photophysical properties were studied, which proved that the IPP core is a modular fluorophore acting *via* an ICT phenomenon. Additionally, preliminary UV-vis and fluorescence spectroscopic studies showed that the products 7a–d could be used as fluorescent chemodosimeters for cyanide detection.

## Conflicts of interest

The authors declare no competing financial interest.

## Supplementary Material

RA-009-C9RA04682H-s001

RA-009-C9RA04682H-s002
