# Supplementary material for: Pyrazolo-fused 4-azafluorenones as key reagents for the synthesis of fluorescent dicyanovinylidene-substituted derivatives
Source: RSC Adv. 2019 Aug 30;9(47):27318–23. doi: 10.1039/c9ra04682h (PMC9070601; doi:10.1039/c9ra04682h)
Supplement: RA-009-C9RA04682H-s001 [file RA-009-C9RA04682H-s001.pdf]

Electronic Supplementary Material (ESI) for RSC Advances.
This journal is © The Royal Society of Chemistry 2019

Supporting information

Pyrazolofused 4-azafluorenones as key reagents for the synthesis of fluorescent dicyanovinilydene-substituted derivatives

Jessica Orrego-Hernández,[a] Carolina Lizarazo,[a] Justo Cobo,[b] and Jaime Portilla*,[a]

[a] *Bioorganic Compounds Research Group, Department of Chemistry, Universidad de los Andes, Carrera 1 No. 18A-10, Bogotá, Colombia*

[b] *Departamento de Química Orgánica e Inorgánica, Universidad de Jaén, 23071 Jaén, Spain*

*E-mail: jportill@uniandes.edu.co; Tel: +57 1 3394949. Ext. 2080

Contents

1. Overview of Substrates, Intermediates and Products Numbering……………………….........S2

2. Experimental procedures and characterization data……………………………………......S4

3. Supplementary Analytical Data (Identification of intermediate **5a**)…………….….………S16

4. Photophysical studies……………………………………………………..…...….....S17

5. Computational calculations………………………..……………...………….…….......S18

6. Fluorescent chemosensors **7a-d** for detection of cyanide………………………...…..........S23

7. Copies of NMR spectra…………………………………………...………………...S25

8. ORTEP Drawing for Structure **7b**…………………..S54

9. HRMS analysis data of the final products **7a-d**……………………………………………..S55

10. References………………………………………………………………………S60

1. Overview of Substrates Intermediates and Products Numbering

*1.1. Substrates **1**, **2a-h**, **3a-e**, and intermediates **6h** and **5a***

Indan-1,3-dione **1**, 4-R-benzaldehydes **2a-h** and 3-alkyl-5-amino-1-aryl-1*H*-pyrazoles **3a-e**

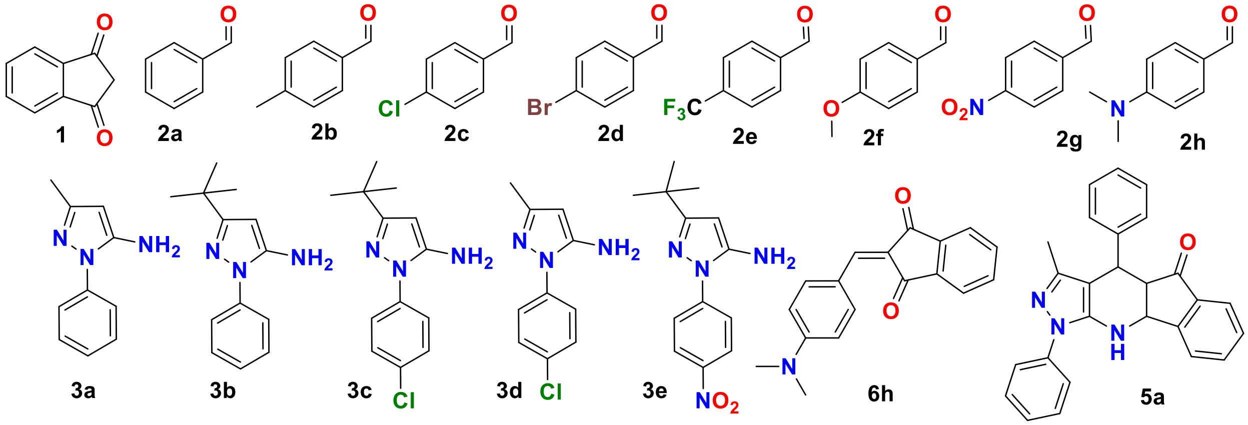

*1.2. Products **4a-x** and final products **7a-d***

4-Aryl-3-methyl-1-phenylindeno[1,2-*b*]pyrazolo[4,3-*e*]pyridin-5(1*H*)-ones **4a-h** and 4-Aryl-3-(*tert*-butyl)-1-phenylindeno[1,2-*b*]pyrazolo[4,3-*e*]pyridin-5(1*H*)-ones **4i-o**.

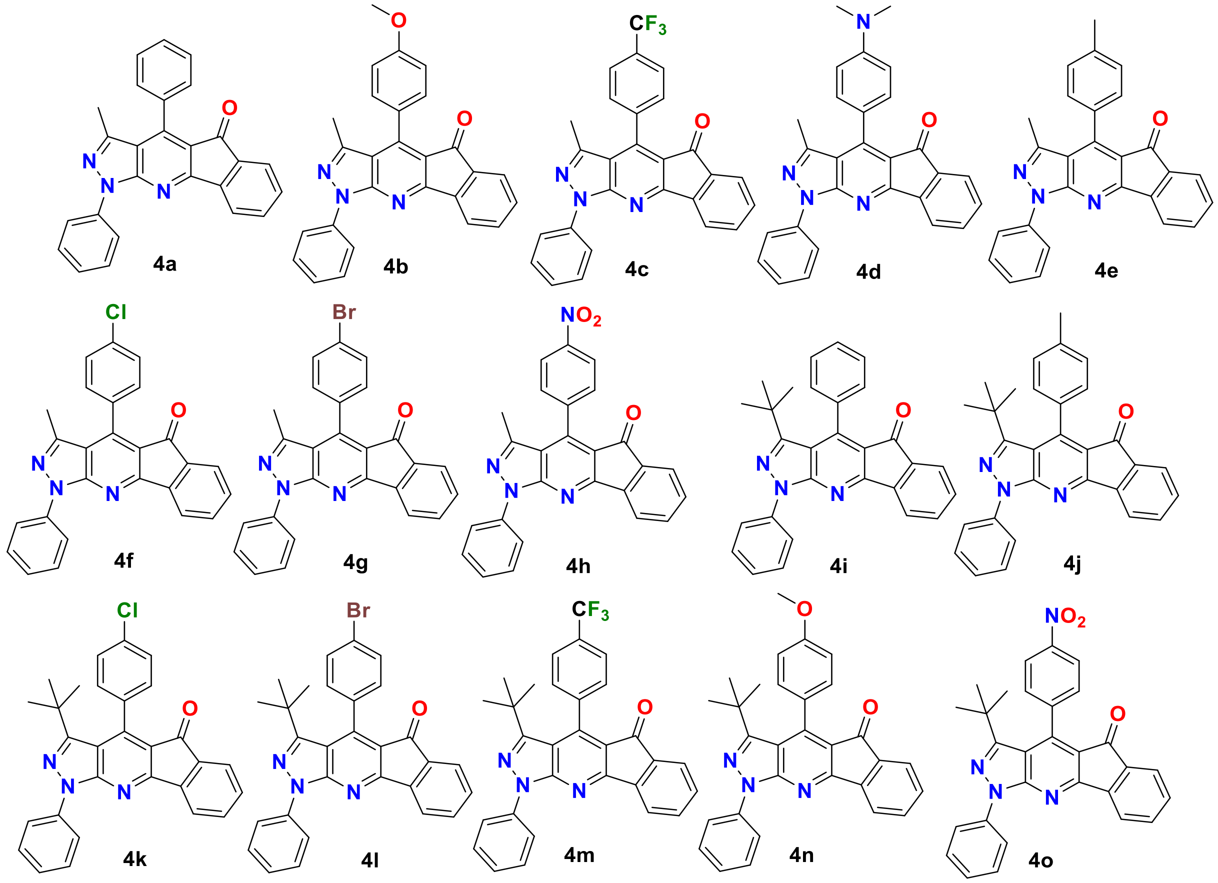

Aryl-3-(*tert*-butyl)-1-(4-chlorophenyl)indeno[1,2-*b*]pyrazolo[4,3-*e*]pyridin-5(1*H*)-ones **4p-v**, 1-(4-chlorophenyl)-3-methyl-4-phenylindeno[1,2-*b*]pyrazolo[4,3-*e*]pyridin-5(1*H*)-one **4w** 3-(*tert*-butyl)-1-(4-nitrophenyl)-4-phenylindeno[1,2-*b*]pyrazolo[4,3-*e*]pyridin-5(1*H*)-one **4x**, and 2-(4-aryl-3-methyl-1-phenylindeno[1,2-*b*]pyrazolo[4,3-*e*]pyridin-5(1*H*)-ylidene)malononitrile **7a-d**.

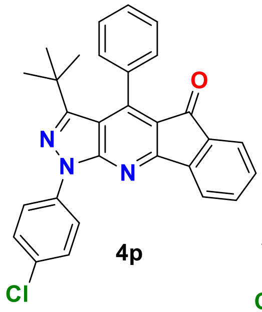

4p

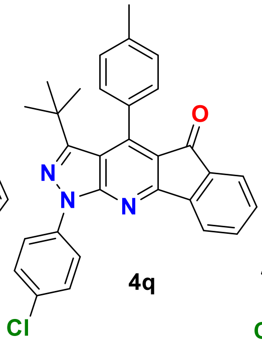

4q

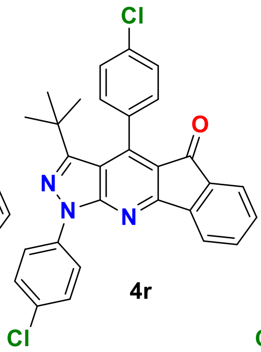

4r

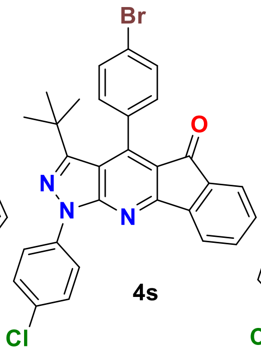

4s

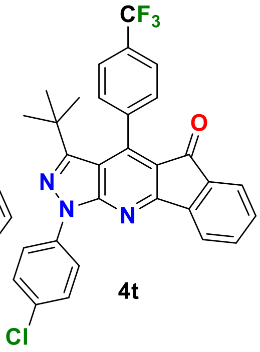

4t

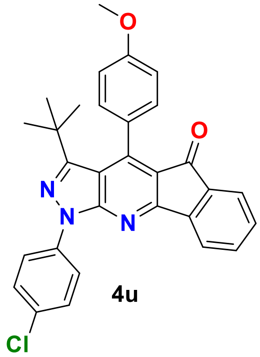

4u

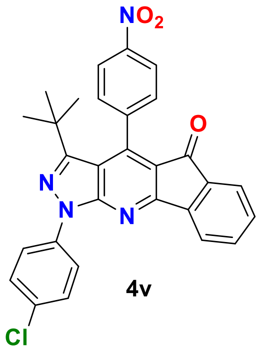

4v

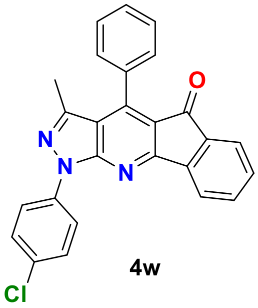

4w

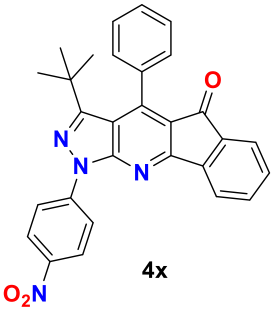

4x

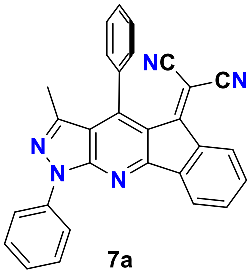

7a

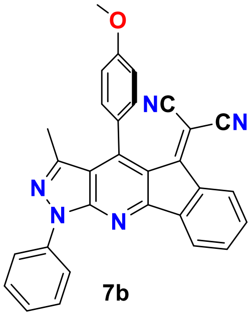

7b

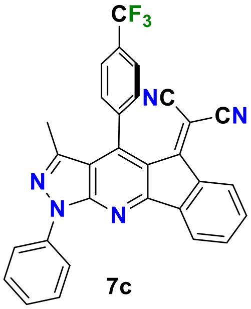

7c

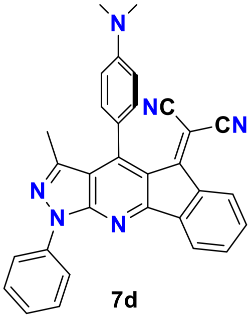

7d

2. Experimental procedures and characterization data

2.1. General information. All reagents were purchased from commercial sources and used without further purification, unless otherwise noted. All starting materials were weighed and handled in air at room temperature. The reactions were monitored by TLC and were visualized by UV (254 nm). Column and Flash chromatography was performed on silica gel (230-400 mesh or 70-230 mesh, respectively). All reactions under microwave (MW) irradiation were performed using a sealed reaction vessel (10 mL, max pressure = 300 psi) containing a Teflon coated stirring bar (obtained from CEM). MW-assisted reactions were performed in a CEM Discover focused microwave (ν = 2.45 GHz) reactor, equipped with a built-in pressure measurement sensor, and with a vertically focused IR temperature sensor; controlled temperature, power, and time settings were used for all reactions. NMR spectra were recorded at 400 MHz (^{1}H) and 100 MHz (^{13}C) at 298 K using tetramethylsilane (0 ppm) as the internal reference. NMR spectroscopic data were recorded in $CDCl_3$ and DMSO-d_6 using as internal standards the residual non-deuterated signal for ^{1}H NMR and the deuterated solvent signal for ^{13}C NMR spectroscopy. DEPT spectra were used for the assignment of carbon signals. Chemical shifts (δ) are given in ppm and coupling constants (J) are given in Hz. The following abbreviations are used for multiplicities: s = singlet, d = doublet, t = triplet, and m = multiplet. Melting points were collected using a Stuart SMP10 melting point apparatus, and the acquired data are uncorrected. The mass spectra were recorded on a Hewlett Packard HP Engine-5989 spectrometer (equipped with a direct inlet probe) and operating at 70 eV, and the High Resolution Mass Spectra (HRMS) by electron impact were recorded on a Micromass AutoSpec-Ultima, magnetic sector mass spectrometer at 70 eV. HPLC-HRMS data were obtained on an Agilent Technologies Q-TOF 6520 spectrometer via an electrospray ionization (ESI, 4000 V). X-ray diffraction intensities were collected on a Bruker D8 Venture diffractometer. Crystallographic data were recorded on a diffractometer using graphite-monochromated Mo Kα radiation (λ= 0.71073 Å). Structures were solved using an interactive algorithm,[1] subsequently completed by a difference Fourier map, and refined using the program SHELXL2014[2] and the graphic material was prepared using the Mercury 3.10 software.[3] The electronic absorption spectra were measured on Varian Cary 100 Conc (Agilent Technologies) spectrophotometer in a quartz cuvette having a path length of 1 cm. The fluorescence emission spectra were recorded by using a CARY Eclipse (Agilent Technologies) fluorescence spectrophotometer in a quartz cell (1 cm path length). UV-vis and fluorescence measurements

were performed at room temperature (20 °C). For fluorescence measurements, both the excitation and emission slit widths were 5 nm.

2.2. Synthesis and characterization

*2.2.1. General Procedure for the synthesis of 3-alkyl-1,4-bis(aryl)indeno[1,2-b]pirazolo[4,3-e]pyridin-5(1H)-ones **4a-x** and 2-(4-dimethylaminobenzylidene)indene-1,3(2H)-dione (**6h**).* A mixture of equimolar quantities (0.25 mmol of each component) of indan-1,3-dione (**1**, **1**, 37 mg), 4-R-benzaldehyde **2**, and 3-alkyl-5-amino-1*H*-pyrazole **3** in water:triethylamine (0.7 ml, 15:1 v/v) was placed in a reaction tube of a CEM Discover, containing a magnetic stirring bar. The tube was sealed with a plastic MW septum and was irradiated at 80 °C (100 W, monitored by an IR temperature sensor) and maintained at this temperature for 10-25 min. The reaction mixture was cooled to 50 °C by airflow and then was partitioned between dichloromethane and water. The organic layer was washed with water and dried over anhydrous sodium sulfate. Subsequently, solvent was removed by rotary evaporation under reduced pressure and the residue was purified by flash chromatography on silica gel (eluent: CH_2Cl_2) to give the expected indenopirazolopyridin-5-ones **4a-x** and the intermediate **6h**.

*3-Methyl-1,4-diphenylindeno[1,2-b]pyrazolo[4,3-e]pyridin-5(1H)-one (**4a**).* By following the general procedure for 15 min, **4a** was obtained as yellow crystals (81.3 mg, 84%). Mp 225-227 °C (Lit.[4] 220-221 °C). ^{1}H NMR ($CDCl_3$): δ = 2.05 (s, 3H), 7.35 (t, *J* = 7.5 Hz, 1H), 7.40-7.47 (m, 3H), 7.50-7.62 (m, 7H), 7.96 (d, *J* = 7.4 Hz, 1H), 8.31 (d, *J* = 8.7 Hz, 2H); ^{13}C NMR ($CDCl_3$): δ = 14.8 (CH_3), 115.6 (C), 120.0 (C), 121.4 (CH), 121.5 (CH), 123.4 (CH), 126.3 (CH), 128.0 (CH), 128.6 (CH), 129.0 (CH), 129.2 (CH), 131.4 (CH), 132.8 (C), 134.6 (CH), 137.4 (C), 139.0 (C), 142.4 (C), 145.8 (C), 145.9 (C), 152.8 (C), 165.1 (C), 189.9 (C); MS (EI) m/z 387 (M^+, 100%), 386 45), 372 (5), 345 (5). HRMS (IE) m/z calcd. for $C_{26}H_{17}N_3O$ $[M]^+$: 387.1372; found 387.1366.

*4-(4-Methoxyphenyl)-3-methyl-1-phenylindeno[1,2-b]pyrazolo[4,3-e]pyridin-5(1H)-one (**4b**).* By following the general procedure for 15 min, **4b** was obtained as yellow crystals (77.3 mg, 74%). Mp 244-246 °C (Lit.[4] 224-225 °C). ^{1}H NMR ($CDCl_3$): δ = 2.12 (s, 3H), 3.91 (s, 3H), 7.06 (d, *J* = 8.9 Hz, 2H), 7.34 (t, *J* = 7.4 Hz, 1H), 7.41 (m, 3H), 7.52-7.62 (m, 4H), 7.95 (d, *J* = 7.5 Hz, 1H), 8.31 (d, *J* = 8.8 Hz, 2H); ^{13}C NMR ($CDCl_3$): δ = 15.2 (CH_3), 55.3 (CH_3), 113.4 (CH), 115.8 (C), 120.0 (C), 121.4 (CH), 121.6 (CH), 123.4 (CH), 124.6 (C), 129.0 (CH), 130.5 (CH), 131.4 (CH),

134.6 (CH), 137.4 (C), 139.0 (C), 142.4 (C), 146.0 (C), 146.1 (C), 152.8 (C), 160.5 (C), 165.2 (C), 190.0 (C); MS (EI) m/z 417 (M^+, 100%), 402 (4), 386 (15). HRMS (IE) m/z calcd. for $C_{27}H_{19}N_3O_2$ $[M]^+$: 417.1477; found 417.1476.

3-Methyl-1-phenyl-4-(4-(trifluoromethyl)phenyl)indeno[1,2-b]pyrazolo[4,3-e]pyridin-5(1H)-one (4c). By following the general procedure for 10 min, **4c** was obtained as white powder (104.8 mg, 92%). Mp 265-267 °C (Lit.[4] 280-281 °C). ^{1}H NMR ($CDCl_3$): δ = 2.05 (s, 3H), 7.37 (t, *J* = 7.4 Hz, 1H), 7.45 (t, *J* = 7.4 Hz, 1H), 7.54-7.63 (m, 6H), 7.80 (d, *J* = 8.1 Hz, 2H), 7.99 (d, *J* = 7.3 Hz, 1H), 8.30 (d, *J* = 8.6 Hz, 2H); ^{13}C NMR ($CDCl_3$): δ = 14.9 (CH_3), 115.1 (C), 119.9, 122.7, 125.4, 128.1 (CF_3, q, J = 367.5 Hz), 120.0 (C), 121.6 (CH), 121.7 (CH), 123.6 (CH), 125.1 (CH, q, J = 3.7 Hz), 126.6 (CH), 129.1 (CH), 129.2 (CH), 130.8, 131.2, 131.5, 131.8 (C-CF_3, q, J = 33.7 Hz), 131.7 (CH), 135.0 (CH), 136.6 (C), 137.3 (C), 138.8 (C), 143.8 (C), 145.5 (C), 152.8 (C), 165.0 (C), 189.8 (C); MS (EI) m/z 455 (M^+, 100%), 440 (6), 413 (6), 386 (5). HRMS (IE) m/z calcd. for $C_{27}H_{16}F_3N_3O$ $[M]^+$: 455.1245; found 455.1241.

4-(4-(Dimethylamino)phenyl)-3-methyl-1-phenylindeno[1,2-b]pyrazolo[4,3-e]pyridin-5(1H)-one (4d). By following the general procedure for 25 min, **4d** was obtained as orange crystals (90.4 mg, 84%). Mp 223-225 °C (Lit.[4] 242-244 °C). ^{1}H NMR ($CDCl_3$): δ = 2.22 (s, 3H), 3.07 (s, 6H), 6.83 (d, *J* = 8.9 Hz, 2H), 7.33 (t, *J* = 7.5 Hz, 1H), 7.40 (m, 3H), 7.55 (m, 3H), 7.62 (d, *J* = 7.4 Hz, 1H), 7.95 (d, *J* = 7.4 Hz, 1H), 8.32 (d, *J* = 8.7 Hz, 2H); ^{13}C NMR ($CDCl_3$): δ = 15.6 (CH_3), 40.3 (CH_3), 111.0 (CH), 115.8 (C), 119.4 (C), 119.8 (C), 121.2 (CH), 121.6 (CH), 123.3 (CH), 126.2 (CH), 129.0 (CH), 130.8 (CH), 131.2 (CH),134.4 (CH), 137.6 (C), 139.1 (C), 142.4 (C), 146.2 (C), 147.4 (C), 151.1 (C), 153.0 (C), 165.4 (C), 190.1 (C); MS (EI) m/z 430 (M^+, 100%), 415 (5), 386 (7). HRMS (IE) m/z calcd. for $C_{28}H_{22}N_4O$ $[M]^+$: 430.1778; found 430.1794.

3-Methyl-1-phenyl-4-(p-tolyl)indeno[1,2-b]pyrazolo[4,3-e]pyridin-5(1H)-one (4e). By following the general procedure for 15 min, **4e** was obtained as pale-yellow crystals (78.3 mg, 78%). Mp >300 °C (Lit.[4] 217-218 °C). ^{1}H NMR ($CDCl_3$): δ = 2.09 (s, 3H), 2.49 (s, 3H), 7.32-7.36 (m, 5H), 7.42 (t, *J* = 7.5 Hz, 1H), 7.53-7.62 (m, 4H), 7.96 (d, *J* = 7.4 Hz, 1H), 8.31 (d, *J* = 8.6 Hz, 2H); ^{13}C NMR ($CDCl_3$): δ = 15.0 (CH_3), 21.6 (CH_3), 115.7 (C), 120.1 (C), 121.4 (CH), 121.6 (CH), 123.4 (CH), 126.3 (CH), 128.7 (CH), 129.0 (CH), 129.7 (C), 131.4 (CH), 134.6 (CH), 137.4 (C), 139.0 (C), 139.1 (C), 142.4 (C), 146.0 (C), 146.3 (C), 152.8 (C), 165.1 (C), 189.9 (C); MS (EI) m/z 401

(M^+, 100%), 386 (41), 324 (8). HRMS (IE) m/z calcd. for $C_{27}H_{19}N_3O$ $[M]^+$: 401.1528; found 401.1517.

*4-(4-Chlorophenyl)-3-methyl-1-phenylindeno[1,2-b]pyrazolo[4,3-e]pyridin-5(1H)-one (**4f**).* By following the general procedure for 10 min, **4f** was obtained as yellow crystals (93.9 mg, 89%). Mp 268-270 °C (Lit.[4] 269-270 °C). ^{1}H NMR ($CDCl_3$): δ = 2.08 (s, 3H), 7.35 (t, *J* = 7.4 Hz, 1H), 7.38-7.44 (m, 3H), 7.50-7.61 (m, 6H), 7.95 (d, *J* = 7.4 Hz, 1H), 8.29 (d, *J* = 8.9 Hz, 2H); ^{13}C NMR ($CDCl_3$): δ = 15.0 (CH_3), 115.3 (C), 120.0 (C), 121.5 (CH), 121.6 (CH), 123.5 (CH), 126.5 (CH), 128.3 (CH), 129.1 (CH), 130.2 (CH), 131.1 (C), 131.6 (CH), 134.8 (CH), 135.4 (C), 137.3 (C), 139.0 (C), 142.3 (C), 144.4 (C), 145.6 (C), 152.7 (C), 165.0 (C), 189.8 (C); MS (EI) m/z 423/421 (M^+, 37/100%), 406 (5), 386 (10). HRMS (IE) m/z calcd. for $C_{26}H_{16}ClN_3O$ $[M]^+$: 421.0982; found 421.0978.

*4-(4-Bromophenyl)-3-methyl-1-phenylindeno[1,2-b]pyrazolo[4,3-e]pyridin-5(1H)-one (**4g**).* By following the general procedure for 10 min, **4g** was obtained as pale-yellow crystals (107.3 mg, 92%). Mp 264-265 °C (Lit.[4] 274-276 °C). ^{1}H NMR ($CDCl_3$): δ = 2.10 (s, 3H), 7.33-7.38 (m, 3H), 7.50-7.64 (m, 5H), 7.67 (d, *J* = 8.9 Hz, 2H), 7.98 (d, J = 7.4 Hz, 1H), 8.29 (d, *J* = 8.9 Hz, 2H); ^{13}C NMR ($CDCl_3$): δ = 15.0 (CH_3), 115.2 (C), 119.9 (C), 121.5 (CH), 121.6 (CH), 123.6 (CH), 123.7 (C), 126.5 (CH), 129.1 (CH), 130.4 (CH), 131.3 (CH), 134.8 (CH), 135.8 (C), 138.9 (C), 142.4 (C), 144.3 (C), 145.6 (C), 152.8 (C), 165.1 (C), 189.8 (C); MS (EI) m/z 467/465 (M^+, 97/100%), 450 (5), 386 (15). HRMS (IE) m/z calcd. for $C_{26}H1_6BrN_3O$ $[M]^+$: 465.0477; found 465.0494.

*3-Methyl-4-(4-nitrophenyl)-1-phenylindeno[1,2-b]pyrazolo[4,3-e]pyridin-5(1H)-one (**4h**).* By following the general procedure for 10 min, **4h** was obtained as pale-yellow crystals (77.8 mg, 72%). Mp 245-246 °C (Lit.[4] >300 °C). ^{1}H NMR ($CDCl_3$): δ = 2.05 (s, 3H), 7.38 (t, *J* = 7.4 Hz, 1H), 7.46 (t, *J* = 7.4 Hz, 1H), 7.57 (t, *J* = 7.6 Hz, 1H), 7.64 (m, 4H), 8.00 (d, *J* = 7.9 Hz, 1H), 8.29 (d, *J* = 7.6 Hz, 2H), 8.41 (d, *J* = 8.7 Hz, 2H); ^{13}C NMR ($CDCl_3$): δ = 14.9 (CH_3), 114.7 (C), 119.9 (C), 121.7 (CH), 123.3 (CH), 123.7 (CH), 126.7 (CH), 131.8 (CH), 135.1 (CH), 137.2 (C), 138.7 (C), 139.6 (C), 142.3 (C), 142.5 (C), 145.1 (C), 148.4 (C), 152.8 (C), 165.0 (C), 189.7 (C); MS (EI) m/z 432 (M^+, 100%), 417 (3), 386 (9), 345 (5). HRMS (IE) m/z calcd. for $C_{26}H_{16}N_4O_3$ $[M]^+$: 432.1222; found 432.1231.

*3-(tert-Butyl)-1,4-diphenylindeno[1,2-b]pyrazolo[4,3-e]pyridin-5(1H)-one (**4i**).* By following the general procedure for 15 min, **4i** was obtained as yellow crystals (83.8 mg, 78%). Mp 230-232 °C; ^{1}H NMR ($CDCl_3$): δ = 1.15 (s, 9H), 7.36 (m, 3H), 7.42 (t, *J* = 7.4 Hz, 1H), 7.49-7.61 (m, 7H), 7.99 (d, *J* = 7.4 Hz, 1H), 8.37 (d, *J* = 8.7 Hz, 2H); ^{13}C NMR ($CDCl_3$): δ = 30.2 (CH3), 34.2 (C), 114.8 (C), 121.0 (C), 121.3 (CH), 122.0 (CH), 123.4 (CH), 126.1 (CH), 127.9 (CH), 128.7 (CH), 128.8 (CH), 129.0 (CH), 131.4 (CH), 134.6 (CH), 136.2 (C), 137.6 (C), 139.2 (C), 142.1 (C), 146.2 (C), 153.6 (C), 157.0 (C), 163.9 (C), 190.0 (C); MS (EI) m/z 429 (M^+, 73%), 414 (100), 399 (23), 384 (24). HRMS (IE) m/z calcd. for $C_{29}H_{23}N_3O$ $[M]^+$: 429.1841; found 429.1843.

*3-(tert-Butyl)-1-phenyl-4-(p-tolyl)indeno[1,2-b]pyrazolo[4,3-e]pyridin-5(1H)-one (**4j**).* By following the general procedure for 15 min, **4j** was obtained as pale-yellow crystals (85.4 mg, 77%). Mp 207-208 °C; ^{1}H NMR ($CDCl_3$): δ = 1.16 (s, 9H), 2.50 (s, 3H), 7.24 (d, *J* = 8.6 Hz, 2H), 7.32-7.39 (m, 3H), 7.41 (t, *J* = 7.4 Hz, 1H), 7.54-7.61 (m, 4H), 7.98 (d, *J* = 7.4 Hz, 1H), 8.37 (d, *J* = 8.6 Hz, 2H); ^{13}C NMR ($CDCl_3$): δ = 21.6 (CH_3), 30.3 (CH_3), 34.3 (C), 115.1 (C), 121.2 (C), 121.3 (CH), 122.0 (CH), 123.4 (CH), 126.3 (CH), 128.5 (CH), 128.6 (CH), 128.9 (CH), 131.4 (CH), 133.2 (C), 134.6 (CH), 137.6 (C), 138.6 (C), 139.2 (C), 142.1 (C), 146.6 (C), 153.5 (C), 157.1 (C), 163.9 (C), 190.1 (C); MS (EI) m/z 443 (M^+, 70%), 428 (100), 413 (25), 398 (28). HRMS (IE) m/z calcd. for $C_{30}H_{25}N_3O$ $[M]^+$: 443.1998; found 443.2004.

*3-(tert-Butyl)-4-(4-chlorophenyl)-1-phenylindeno[1,2-b]pyrazolo[4,3-e]pyridin-5(1H)-one (**4k**).* By following the general procedure for 10 min, **4k** was obtained as yellow crystals (96.1 mg, 83%). Mp 235-237 °C; ^{1}H NMR ($CDCl_3$): δ = 1.16 (s, 9H), 7.30 (d, *J* = 8.5 Hz, 2H), 7.36 (t, *J* = 7.5 Hz, 1H), 7.42 (t, *J* = 7.4 Hz, 1H), 7.51 (d, *J* = 8.6 Hz, 1H), 7.54-7.62 (m, 4H), 7.98 (d, *J* = 7.4 Hz, 1H), 8.36 (d, *J* = 8.7 Hz, 2H); ^{13}C NMR ($CDCl_3$): δ = 30.4 (CH_3), 34.2 (C), 114.6 (C), 120.9 (C), 121.4 (CH), 122.0 (CH), 123.5 (CH), 126.4 (CH), 128.3 (CH), 129.0 (CH), 130.2 (CH), 131.6 (CH), 134.6 (C), 134.8 (CH), 135.0 (C), 137.5 (C), 139.1 (C), 142.0 (C), 144.7 (C), 153.5 (C), 156.8 (C), 163.8 (C), 190.0 (C); MS (EI) m/z 465/463 (M^+, 23/65%), 450/448 (36/100), 435/433 (6/18). HRMS (IE) m/z calcd. for $C_{29}H_{22}ClN_3O$ $[M]^+$: 463.1451; found 463.1458.

*4-(4-Bromophenyl)-3-(tert-butyl)-1-phenylindeno[1,2-b]pyrazolo[4,3-e]pyridin-5(1H)-one (**4l**).* By following the general procedure for 10 min, **4l** was obtained as yellow crystals (119.5 mg, 94%). Mp 233-235 °C; ^{1}H NMR ($CDCl_3$): δ = 1.16 (s, 9H), 7.24 (d, J = 8.1 Hz, 2H), 7.36 (t, *J* =

7.5 Hz, 1H), 7.42 (t, *J* = 7.4 Hz, 1H), 7.54-7.61 (m, 4H), 7.65 (d, *J* = 8.2 Hz, 1H), 7.97 (d, *J* = 7.4 Hz, 1H), 8.35 (d, *J* = 8.7 Hz, 2H); ^{13}C NMR ($CDCl_3$): δ = 30.4 (CH_3), 34.2 (C), 114.5 (C), 121.4 (C), 122.0 (CH), 123.1 (C), 123.5 (CH), 126.4 (CH), 129.0 (CH), 130.5 (CH), 131.2 (CH), 131.6 (CH), 134.8 (CH), 135.1 (C), 137.4 (C), 139.1 (C), 142.0 (C), 144.6 (C), 153.5 (C), 156.8 (C), 163.8 (C), 189.9 (C); MS (EI) m/z 509/507 (M^+, 79/69%), 494/492 (100/87), 398 (50). HRMS (IE) m/z calcd. for $C_{29}H_{22}BrN_3O$ $[M]^+$: 507.0946; found 507.0945.

*3-(tert-Butyl)-1-phenyl-4-(4-(trifluoromethyl)phenyl)indeno[1,2-b]pyrazolo[4,3-e]pyridin-5(1H)-one (**4m**).* By following the general procedure for 10 min, **4m** was obtained as pale-yellow crystals (112.0 mg, 90%). Mp 250-252 °C; ^{1}H NMR ($CDCl_3$): δ = 1.13 (s, 9H), 7.38 (t, *J* = 7.4 Hz, 1H), 7.43 (t, *J* = 7.5 Hz, 1H), 7.51 (d, *J* = 8.0 Hz, 1H), 7.56-7.62 (m, 4H), 7.79 (d, *J* = 7.9 Hz, 2H), 7.99 (d, *J* = 7.4 Hz, 1H), 8.36 (d, *J* = 8.7 Hz, 2H); ^{13}C NMR ($CDCl_3$): δ = 30.3 (CH_3), 34.2 (C), 114.3 (C), 120.7 (C), 121.5 (CH), 122.1 (CH), 123.5 (CH), 125.0 (CH, q, *J* = 3.7 Hz,), 126.5 (CH), 129.0 (CH), 129.4 (CH), 130.6, 131.0, 131.3, 131.6 (C-CF3, q, *J* = 33.0 Hz), 131.7 (CH), 134.9 (CH), 137.4 (C), 139.1 (C), 140.1 (C), 142.0 (C), 144.2 (C), 153.5 (C), 156.7 (C), 163.8 (C), 189.89 (C); MS (EI) m/z 497 (M^+, 58%), 482 (100), 455 (18). HRMS (IE) m/z calcd. for $C_{30}H_{22}F_3N_3O$ $[M]^+$: 497.1715; found 497.1718.

*3-(tert-Butyl)-4-(4-methoxyphenyl)-1-phenylindeno[1,2-b]pyrazolo[4,3-e]pyridin-5(1H)-one (**4n**).* By following the general procedure for 10 min, **4n** was obtained as orange crystals (89.5 mg, 78%). Mp 228-230 °C; ^{1}H NMR ($CDCl_3$): δ = 1.17 (s, 9H), 3.92 (s, 3H), 7.05 (d, *J* = 8.7 Hz, 2H), 7.27 (d, *J* = 8.7 Hz, 2H), 7.35 (t, *J* = 7.4 Hz, 1H), 7.54-7.61 (m, 4H), 7.98 (d, *J* = 7.3 Hz, 1H), 8.37 (d, *J* = 8.7 Hz, 2H); ^{13}C NMR ($CDCl_3$): δ = 30.3 (CH_3), 34.3 (C), 55.3 (CH_3), 113.4 (CH), 115.3 (C), 121.3 (CH), 121.4 (C), 122.0 (CH), 123.4 (CH), 126.3 (CH), 128.3 (C), 129.0 (CH), 130.0 (CH), 131.4 (CH), 134.6 (CH), 137.6 (C), 139.2 (C), 142.1 (C), 146.4 (C), 153.6 (C), 157.1 (C), 160.0 (C), 163.9 (C), 190.2 (C); MS (EI) m/z 459 (M^+, 79%), 444 (100), 429 (36), 414 (21). HRMS (IE) m/z calcd. for $C_{30}H_{25}N_3O_2$ $[M]^+$: 459.1947; found 459.1931.

*3-(tert-Butyl)-4-(4-nitrophenyl)-1-phenylindeno[1,2-b]pyrazolo[4,3-e]pyridin-5(1H)-one (**4o**).* By following the general procedure for 10 min, **4o** was obtained as pale-orange crystals (89.0 mg, 75%). Mp 270-272 °C; ^{1}H NMR ($CDCl_3$): δ = 1.14 (s, 9H), 7.39 (t, *J* = 7.4 Hz, 1H), 7.45 (t, *J* = 7.5 Hz, 1H), 7.56-764 (m, 6H), 8.00 (d, *J* = 7.4 Hz, 1H), 8.35 (d, *J* = 8.6 Hz, 2H), 8.40 (d, *J* = 8.7

Hz, 2H); ^{13}C NMR ($CDCl_3$): δ = 30.4 (CH_3), 34.2 (C), 113.8 (C), 120.5 (C), 121.6 (CH), 122.1 (CH), 123.3 (CH), 123.6 (CH), 126.7 (CH), 129.0 (CH), 130.2 (CH), 131.8 (CH), 135.0 (CH), 137.3 (C), 139.0 (C), 142.0 (C), 143.0 (C), 143.2 (C), 148.2 (C), 156.5 (C), 163.8 (C), 189.8 (C); MS (EI) m/z 474 (M^+, 53%), 459 (100), 432 (15), 398 11). HRMS (IE) m/z calcd. for $C_{29}H_{22}N_4O_3$ $[M]^+$: 474.1692; found 474.1696.

*3-(tert-Butyl)-1-(4-chlorophenyl)-4-phenylindeno[1,2-b]pyrazolo[4,3-e]pyridin-5(1H)-one (**4p**).* By following the general procedure for 15 min, **4p** was obtained as yellow crystals (99.7 mg, 86%). Mp 233-235 °C (Lit.[5] 237-238 °C); ^{1}H NMR ($CDCl_3$): δ = 1.14 (s, 9H), 7.35 (d, *J* = 8.7 Hz, 2H), 7.43 (t, *J* = 7.5 Hz, 1H), 7.49-762 (m, 7H), 7.96 (d, *J* = 7.5 Hz, 1H) and 8.35 (d, *J* = 8.9 Hz, 2H); ^{13}C NMR ($CDCl_3$): δ = 30.2 (CH_3), 34.3 (C), 115.0 (C), 121.1 (C), 121.3 (CH), 122.9 (CH), 123.5 (CH), 127.9 (CH), 128.7 (CH), 128.9 (CH), 129.0 (CH), 131.5 (C), 131.6 (CH), 134.7 (CH), 136.0 (C), 137.5 (C), 137.8 (C), 141.9 (C), 146.3 (C), 153.5 (C), 157.3 (C), 164.0 (C), 189.8 (C); MS (EI) m/z 465/463 (M^+, 21/71%), 450/448 (37/100), 435/433 (6/14), 420/418 (19/21). HRMS (IE) m/z calcd. for $C_{29}H_{22}ClN_3O$ $[M]^+$: 463.1451; found 463.1469. The characterization data for **4p** match previously reported data by us.[5]

*3-(tert-Butyl)-1-(4-chlorophenyl)-4-(p-tolyl)indeno[1,2-b]pyrazolo[4,3-e]pyridin-5(1H)-one (**4q**).* By following the general procedure for 15 min, **4q** was obtained as yellow crystals (88.0 mg, 78%). Mp 213-215 °C; ^{1}H NMR ($CDCl_3$): δ = 1.15 (s, 9H), 2.50 (s, 3H), 7.23 (d, *J* = 8.0 Hz, 2H), 7.33 (d, *J* = 7.9 Hz, 2H), 7.42 (t, *J* = 7.4 Hz, 1H), 7.52 (d, *J* = 9.0 Hz, 2H), 7.56-761 (m, 2H), 7.96 (d, *J* = 7.5 Hz, 1H), 8.38 (d, *J* = 8.9 Hz, 2H); ^{13}C NMR ($CDCl_3$): δ = 21.6 (CH_3), 30.2 (CH_3), 34.3 (C), 115.2 (C), 121.2 (CH), 121.3 (C), 122.9 (CH), 123.5 (CH), 128.5 (CH), 128.6 (CH), 129.0 (CH), 129.7 (C), 131.5 (CH), 133.0 (C), 134.6 (CH), 137.6 (C), 137.8 (C), 138.7 (C), 141.9 (C), 146.7 (C), 153.5 (C), 157.4 (C), 164.0 (C), 189.9 (C); MS (EI) m/z 479/477 (M^+, 27/76%), 464/462 (38/100), 449/447 (9/27), 434/432 (27/32). HRMS (IE) m/z calcd. for $C_{30}H_{24}ClN_3O$ $[M]^+$: 447.1608; found 447.1605.

*3-(tert-Butyl)-1,4-bis(4-chlorophenyl)indeno[1,2-b]pyrazolo[4,3-e]pyridin-5(1H)-one (**4r**).* By following the general procedure for 15 min, **4r** was obtained as yellow crystals (99.7 mg, 80%). Mp 260-262 °C; ^{1}H NMR ($CDCl_3$): δ = 1.15 (s, 9H), 7.29 (d, *J* = 8.7 Hz, 2H), 7.43 (t, *J* = 7.5 Hz, 1H), 7.49-762 (m, 6H), 7.97 (d, *J* = 7.4 Hz, 1H), 8.36 (d, *J* = 9.0 Hz, 2H); ^{13}C NMR ($CDCl_3$): δ = 30.3 (CH_3), 34.3 (C), 114.8 (C), 121.1 (C), 121.4 (CH), 122.9 (CH), 123.6 (CH), 128.3 (CH),

129.0 (CH), 130.2 (CH), 131.6 (CH), 131.7 (C), 134.5 (C), 134.8 (CH), 135.1 (C), 137.4 (C), 137.7 (C), 141.8 (C), 144.8 (C), 153.5 (C), 157.1 (C), 163.9 (C), 189.8 (C); MS (EI) m/z 501/500/499/497 (M^+, 7/10/52/68%), 484/483/482/467/457 (75/11/100/13/23). HRMS (IE) m/z calcd. for $C_{29}H_{21}Cl_2N_3O$ $[M]^+$: 497.1062; found 497.1056.

*4-(4-Bromophenyl)-3-(tert-butyl)-1-(4-chlorophenyl)indeno[1,2-b]pyrazolo[4,3-e]pyridin-5(1H)-one (**4s**)*. By following the general procedure for 15 min, **4s** was obtained as pale-yellow crystals (124.8 mg, 92%). Mp 252-254 °C; ^{1}H NMR ($CDCl_3$): δ = 1.15 (s, 9H), 7.23 (d, J = 8.4 Hz, 2H), 7.43 (t, J = 7.5 Hz, 1H), 7.52 (d, J = 9.0 Hz, 2H), 7.57-763 (m, 2H), 7.66 (d, J = 8.3 Hz, 2H), 7.97 (d, J = 7.5 Hz, 1H), 8.36 (d, J = 8.9 Hz, 2H); ^{13}C NMR ($CDCl_3$): δ = 30.3 (CH_3), 34.3 (C), 114.7 (C), 121.0 (C), 121.4 (CH), 123.0 (CH), 123.2 (C), 123.6 (CH), 129.1 (CH), 130.5 (CH), 131.2 (CH), 131.7 (CH), 131.8 (C), 134.9 (CH), 135.0 (C), 135.1 (C), 137.4 (C), 137.7 (C), 141.8 (C), 144.7 (C), 153.5 (C), 157.1 (C), 163.9 (C), 189.8 (C); MS (EI) m/z 545/544/543/541 (M^+, 21/20/100/68%), 530/529/528/526/513 (14/17/98/70/15). HRMS (IE) m/z calcd. for $C_{29}H_{21}BrClN_3O$ $[M]^+$: 541.0557; found 541.0541.

*3-(tert-Butyl)-1-(4-chlorophenyl)-4-(4-(trifluoromethyl)phenyl)indeno[1,2-b]pirazolo[4,3-e]pyridin-5(1H)-one (**4t**)*. By following the general procedure for 15 min, **4t** was obtained as yellow crystals (126.4 mg, 95%). Mp 226-227 °C; ^{1}H NMR ($CDCl_3$): δ = 1.12 (s, 9H), 7.44 (t, J = 7.4 Hz, 1H), 7.50 (d, J = 8.0 Hz, 2H), 7.53 (d, J = 8.9 Hz, 2H), 7.57 (d, J = 7.4 Hz, 1H), 7.62 (t, J = 7.5 Hz, 1H), 7.79 (d, J = 8.1 Hz, 2H), 7.98 (d, J = 7.4 Hz, 1H), 8.36 (d, J = 9.0 Hz, 2H); ^{13}C NMR ($CDCl_3$): δ = 30.2 (CH_3), 34.2 (C), 114.4 (C), 120.0, 122.7, 125.4, 128.1 (CF_3, q, J = 272.2 Hz), 120.9 (C), 121.5 (CH), 123.0 (CH), 123.6 (CH), 125.0 (CH, q, J = 3.7 Hz), 129.1 (CH), 129.4 (CH), 130.7, 131.0, 131.4, 131.7 (C-CF_3, q, J = 32.3 Hz), 131.7 (CH), 131.8 (C), 135.0 (CH), 137.4 (C), 137.7 (C), 139.9 (C), 141.8 (C), 144.2 (C), 153.5 (C), 157.0 (C), 163.9 (C), 189.7 (C); MS (EI) m/z 533/531 (M^+, 27/68%), 518/516 (35/100), 489/487 (6/16). HRMS (IE) m/z calcd. for $C_{30}H_{21}ClF_3N_3O$ $[M]^+$: 531.1325; found 531.1304.

*3-(tert-Butyl)-1-(4-chlorophenyl)-4-(4-methoxyphenyl)indeno[1,2-b]pyrazolo[4,3-e]pyridin-5(1H)-one (**4u**)*. By following the general procedure for 20 min, **4u** was obtained as pale-orange crystals (105.0 mg, 79%). Mp 225-227 °C (Lit.[5] 230-231 °C); ^{1}H NMR ($CDCl_3$): δ = 1.16 (s, 9H), 3.92 (s, 3H), 7.05 (d, J = 8.7 Hz, 2H), 7.26 (d, J = 8.7 Hz, 2H), 7.42 (t, J = 7.4 Hz, 1H), 7.51 (d, J

= 8.9 Hz, 2H), 7.56-761 (m, 2H), 7.96 (d, *J* = 7.4 Hz, 1H), 8.37 (d, *J* = 9.0 Hz, 2H); ^{13}C NMR ($CDCl_3$): δ = 30.2 (CH_3), 34.4 (C), 55.3 (CH_3), 113.4 (CH), 115.5 (C), 121.3 (CH), 121.5 (C), 122.9 (CH), 123.5 (CH), 128.1 (CH), 129.0 (CH), 130.0 (CH), 131.4 (C), 131.5 (CH), 134.7 (CH), 137.6 (C), 137.8 (C), 138.7 (C), 141.9 (C), 146.5 (C), 153.5 (C), 157.4 (C), 164.0 (C), 190.0 (C); MS (EI) m/z 495/493 (M^+, 28/77%), 480/478 (36/100), 465/463 (11/37). HRMS (IE) m/z calcd. for $C_{30}H_{24}ClN_3O_2$ $[M]^+$: 493.1557; found 493.1544. The characterization data for **4p** match previously reported data by us.[5]

*3-(tert-Butyl)-1-(4-chlorophenyl)-4-(4-nitrophenyl)indeno[1,2-b]pyrazolo[4,3-e]pyridin-5(1H)-one (**4v**).* By following the general procedure for 15 min, **4v** was obtained as yellow crystals (99.2 mg, 78%). Mp 288-290 °C; ^{1}H NMR ($CDCl_3$): δ = 1.14 (s, 9H), 7.45 (t, *J* = 7.4 Hz, 1H), 7.51-758 (m, 5H), 7.63 (t, *J* = 7.4 Hz, 1H), 7.99 (d, *J* = 7.5 Hz, 1H), 8.36 (d, *J* = 9.1 Hz, 1H), 8.40 (d, *J* = 8.7 Hz, 1H); ^{13}C NMR ($CDCl_3$): δ = 30.4 (CH_3), 34.2 (C), 114.0 (C), 120.6 (C), 121.6 (CH), 123.0 (CH), 123.3 (CH), 123.7 (CH), 129.1 (CH), 130.2 (CH), 131.9 (CH), 132.0 (C), 135.1 (CH), 137.3 (C), 137.6 (C), 141.8 (C), 143.0 (C), 148.3 (C), 153.5 (C), 156.8 (C), 163.9 (C), 189.6 (C); MS (EI) m/z 510/508 (M^+, 23/67%), 495/493 (36/100), 468/466 (5/14). HRMS (IE) m/z calcd. for $C_{29}H_{21}ClN_4O_3$ $[M]^+$: 508.1302; found 508.1300.

*1-(4-Chlorophenyl)-3-methyl-4-phenylindeno[1,2-b]pyrazolo[4,3-e]pyridin-5(1H)-one (**4w**).* By following the general procedure for 15 min, **4w** was obtained as yellow crystals (84.4 mg, 80%). Mp 240-242 °C; ^{1}H NMR ($CDCl_3$): δ = 2.04 (s, 3H), 7.40-7.46 (m, 3H), 7.48-7.55 (m, 5H), 7.58-7.63 (m, 2H), 7.96 (d, *J* = 7.5 Hz, 1H), 8.32 (d, *J* = 8.9 Hz, 2H); ^{13}C NMR ($CDCl_3$): δ = 14.8 (CH_3), 115.7 (C), 120.2 (C), 121.4 (CH), 122.4 (CH), 123.5 (CH), 128.0 (CH), 128.6 (CH), 129.1 (CH), 129.2 (CH), 131.6 (CH), 132.6 (C), 134.8 (CH), 137.3 (C), 137.6 (C), 142.3 (C), 145.9 (C), 146.2 (C), 152.8 (C), 165.2 (C), 189.8 (C); MS (EI) m/z 423/421 (M^+, 36/100%). HRMS (IE) m/z calcd. for $C_{26}H_{16}ClN_3O$ $[M]^+$: 421.0982; found 421.0988.

*3-(tert-Butyl)-1-(4-nitrophenyl)-4-phenylindeno[1,2-b]pyrazolo[4,3-e]pyridin-5(1H)-one (**4x**).* By following the general procedure for 15 min, **4x** was obtained as yellow crystals (89.0 mg, 75%). Mp 245-247 °C; ^{1}H NMR (DMSO-d_6, 120 °C): δ = 1.11 (s, 9H), 7.39 (d, *J* = 7.9 Hz, 2H), 7.47-756 (m, 6H), 8.05 (d, *J* = 7.4 Hz, 1H), 8.42 (d, *J* = 9.2 Hz, 2H), 8.71 (d, *J* = 9.1 Hz, 2H); ^{13}C NMR (DMSO-d_6, 120 °C): δ = 29.2 (CH_3), 33.4 (C), 114.7 (C), 120.0 (C), 120.5 (CH), 120.7 (CH), 122.1 (CH), 124.0 (CH), 126.9 (CH), 128.1 (CH), 128.2 (CH), 131.4 (CH), 134.4 (CH), 134.8 (C),

135.0 (C), 136.4 (C), 140.4 (C), 143.0 (C), 144.4 (C), 145.6 (C), 157.6 (C), 163.2 (C), 187.5 (C);MS (EI) m/z 474 (M^+, 30%), 459 (100), 444 (33), 429 (17). HRMS (IE) m/z calcd. for $C_{29}H_{22}N_4O_3$ $[M]^+$: 474.1692; found 474.1692.

2-(4-(Dimethylamino)benzylidene)-1H-indene-1,3(2H)-dione ***6h***. By following the general procedure for 10 min, **6h** was obtained as bright-orange crystals (65.8 mg, 95%). Mp 192-193 °C (Lit. [37] 203 °C). ^{1}H NMR ($CDCl_3$): δ = 3.13 (s, 6H), 6.73 (d, *J* = 9.2 Hz, 2H), 7.71 (m, 2H), 7.77 (s, 1H), 7.91 (m, 2H), 8.52 (d, *J* = 9.0 Hz, 2H); ^{13}C NMR ($CDCl_3$): δ = 40.0 (CH_3), 111.4 (CH), 122.0 (C), 122.4 (CH), 123.0 (C), 134.0 (CH), 134.3 (CH), 137.9 (CH), 139.9 (C), 142.2 (C), 147.5 (CH), 153.9 (C), 189.9 (C), 191.7 (C); MS (EI) m/z 277 (M^+, 100%), 260 (7), 233 (10). HRMS (IE) m/z calcd. for $C_{18}H_{15}NO_2$ $[M]^+$: 277.1103; found 277.1096.

2.2.2. General procedure for the synthesis of 2-(4-aryl-3-methyl-1-phenylindeno[1,2-b]pyrazolo[4,3-e]pyridin-5(1H)-ylidene)malononitrile ***7a-d***. A mixture of the appropiate ketone **4a-d** (0.22 mmol) and malononitrile (27 mg, 2.2 mmol) in 20 mL of chlorobenzene was added pyridine (0.36 mL, 4.4 mmol) and $TiCl_4$ (0.24 mL, 2.2 mmol) under argon atmosphere. The mixture was stirred at room temperature for 15 min and it was heated at reflux for 20 h. Then, equal amounts of pyridine and $TiCl_4$ was added and the final mixture was heated at reflux for 4 h. The resulting crude was added 20 mL of water and partitioned with DCM. The organic layer was washed with water and dried over anhydrous Na_2SO_4. Later, the solvent was removed under vacuum and the residue was purified by flash chromatography (eluent: CH_2Cl_2:MeOH 50:1 v/v) to give the pure products **7a-d** as orange solids.

*2-(3-Methyl-1,4-diphenylindeno[1,2-b]pyrazolo[4,3-e]pyridin-5(1H)-ylidene)malononitrile (**7a**)*. By following the general procedure, **7a** was obtained as orange powder (96.0 mg, 96%). Mp. 235-237 °C. ^{1}H NMR ($CDCl_3$): δ = 2.10 (s, 3H), 7.37 (t, *J* = 7.4 Hz, 1H), 7.48-7.65 (m, 9H), 8.05 (d, *J* = 7.0 Hz, 1H), 8.26 (d, *J* = 8.5 Hz, 2H), 8.49 (d, *J* = 7.8 Hz, 1H); ^{13}C NMR ($CDCl_3$): δ = 16.1 (CH_3), 111.7 (C), 114.8 (C), 115.7 (C), 121.7 (CH), 121.8 (C), 122.4 (CH), 125.9 (CH), 126.7 (CH), 128.7 (C), 129.2 (CH), 129.6 (CH), 129.9 (CH), 130.6 (CH), 131.5 (CH), 134.1 (CH), 134.2 (C), 138.6 (C), 138.7 (C), 139.9 (C), 146.0 (C), 147.7 (C), 152.3 (C), 160.0 (C), 162.1 (C). HRMS (ESI+) m/z calcd. for $C_{29}H_{18}N_5$ $[M+H]^+$: 436.1562; found 436.1548.

*2-(4-(4-Methoxyphenyl)-3-methyl-1-phenylindeno[1,2-b]pyrazolo[4,3-e]pyridin-5(1H)-ylidene)malononitrile (**7b**)*. By following the general procedure, **7b** was obtained as orange powder (100.0 mg, 98%). Mp. 244-246 °C. Recrystallization of **7b** from DMF afforded crystals of suitable size and quality for single-crystal X-ray diffraction analysis. ^{1}H NMR ($CDCl_3$): δ = 2.19 (s, 3H), 3.93 (s, 3H), 7.14 (d, *J* = 8.7 Hz, 2H), 7.37 (t, *J* = 7.4 Hz, 1H), 7.43 (d, *J* = 8.7 Hz, 2H), 7.49 (t, *J* = 7.6 Hz, 1H), 7.56 (t, *J* = 7.8 Hz, 2H), 7.61 (t, *J* = 7.5 Hz, 1H), 8.04 (d, *J* = 7.0 Hz, 1H), 8.26 (d, *J* = 7.4 Hz, 2H), 8.48 (d, *J* = 7.9 Hz, 1H); ^{13}C NMR ($CDCl_3$): δ = 16.4 (CH_3), 55.5 (CH_3), 111.9 (C), 114.9 (C), 115.2 (CH), 115.9 (C), 121.7 (CH), 122.0 (C), 122.4 (CH), 125.8 (CH), 126.3 (C), 126.6 (CH), 128.3 (C), 129.2 (CH), 131.3 (CH), 131.4 (CH), 134.0 (CH), 138.9 (C), 139.9 (C), 146.0 (C), 160.4 (C), 161.5 (C), 161.9 (C). HRMS (ESI+) m/z calcd. for $C_{30}H_{20}N_5O$ $[M+H]^+$: 466.1668; found 466.1647. Crystal data for **7b** were deposited at CCDC (1886021): Chemical formula $C_{30}H_{19}N_5O$, Mr 187.20, Monoclinic, C2/c, 100 K, cell dimensions a, b, c (Å) 14.878(7), 12.149(6), 27.262(12) A α, β, γ (º) 90, 96.052(12), 90. V (Å^3) 4900 (4), Z = 8, F(000)= 1936, Dx (Mg m-3) = 1.262, Mo Kα, μ (mm-1)= 0.080, Crystal size (mm) = 0.307 x 0.200 x 0.180. Data collection: Diffractometer Bruker D8 Venture (APEX 3), Monochromator multilayer mirror, CCD rotation images, thick slices φ & θ scans, Mo INCOATEC high brilliance microfocus sealed tube (λ= 0.71073 Å), multiscan absorption correction (SADABS-2016/2), Tmin, Tmax 0.6907 0.7456. No. of measured, independent and observed [I > 2σ(I)] reflections 70658, 5639, 4245, Rint= 0.091 (0.041), θ values (°): θmax = 27.5, θmin = 2.25; Range h = -19→19, k = -15→15, l = -35→35, Refinement on F^2:$R[F^2 > 2\sigma(F^2)]$= 0.0637, $wR(F^2)$= 0.0915, S=1.149. No. of reflections 4245, No. of parameters 327, No. of restraints 0. Weighting scheme: $w = 1/\sigma^2(Fo^2) + (0.0346P)^2 + 13.4077P$ where $P = (Fo^2 + 2Fc^2)/3$. (Δ/σ) < 0.001, Δρmax, Δρmin (e Å^{-3}) 0.310, -0.350.

*2-(3-Methyl-1-phenyl-4-(4-(trifluoromethyl)phenyl)indeno[1,2-b]pyrazolo[4,3-e]pyridin-5(1H)-ylidene)malononitrile (**7c**)*. By following the general procedure, **7c** was obtained as orange powder (111.0 mg, 95%). Mp. 272-273 °C. ^{1}H NMR ($CDCl_3$): δ = 2.07 (s, 3H), 7.38 (t, *J* = 7.4 Hz, 1H), 7.49-7.58 (m, 3H), 7.61-7.66 (m, 3H), 7.88 (d, *J* = 8.0 Hz, 2H), 8.06 (d, *J* = 7.1 Hz, 1H), 8.25 (d, *J* = 8.7 Hz, 2H), 8.49 (d, *J* = 7.9Hz, 1H); ^{13}C NMR ($CDCl_3$): δ = 16.2 (CH_3), 111.9 (C), 114.4 (C), 115.2 (C), 121.7 (CH), 122.4 (C), 122.5 (CH), 125.2 (C), 126.0 (CH), 126.6 (CH*o*-CF_3, q, *J* = 3.7 Hz), 126.9 (CH), 129.2 (CH), 130.4 (CH), 131.7 (CH), 132.6 (C*i*-CF_3 q, *J* = 67 Hz), 134.3 (CH),

137.9 (C), 138.5 (C), 138.6 (C), 139.7 (C), 145.5 (C), 145.6 (C), 152.2 (C), 159.8 (C), 162.2 (C). HRMS (ESI+) m/z calcd. for $C_{30}H_{27}F_3N_5$ [M+H]$^+$: 504.1436; found 504.1419.

*2-(4-(4-(Dimethylamino)phenyl)-3-methyl-1-phenylindeno[1,2-b]pyrazolo[4,3-e]pyridin-5(1H)-ylidene)malononitrile (**7d**)*. By following the general procedure, **7d** was obtained as green powder (53.2 mg, 50%). Mp. >300 °C. ^{1}H NMR ($CDCl_3$): δ = 2.29 (s, 3H), 3.10 (s, 6H), 6.88 (d, *J* = 8.7 Hz, 2H), 7.33-7.37 (m, 3H), 7.48 (t, *J* = 7.6 Hz, 1H), 7.53-7.61 (m, 3H), 8.01 (d, *J* = 7.1 Hz, 1H), 8.26 (d, *J* = 8.4 Hz, 2H), 8.44 (d, *J* = 7.9 Hz, 1H); ^{13}C NMR ($CDCl_3$): δ = 16.8 (CH_3), 40.2 (CH_3), 112.0 (C), 112.6 (CH), 115.1 (C), 115.9 (C), 121.0 (C), 121.7 (CH), 122.0 (CH), 122.2 (CH), 125.6 (CH), 126.5 (CH), 129.1 (CH), 131.1 (CH), 131.2 (CH), 133.7 (CH), 138.8 (C), 139.9 (C), 146.2 (C), 149.1 (C), 151.7 (C), 152.6 (C), 161.1 (C), 161.8 (C). HRMS (ESI+) m/z calcd. for $C_{31}H_{23}N_6$ [M+H]$^+$: 479.1984; found 479.1969.

2.3. Photophysical properties. The solvochromic studies of compounds **7a-d** were carried out with 50 μM solutions in toluene (PhMe), dichloromethane (DCM), acetonitrile (ACN), acetone, and dimethyl sulfoxide (DMSO). Fluorescence response in photographs was at an excitation of 365 nm using a UV lamp. The relative quantum yields were obtained using quinine sulfate (ϕ_F = 0.59 in 0.15 M $HClO_4$) [38] as reference and calculated according to the following equation[6-8]

$$\varphi_{f,x} = \varphi_{f,st} \cdot \frac{F_x}{F_{st}} \cdot \frac{1-10^{-A_{st}}}{1-10^{-A_x}} \cdot \frac{\eta_x^2}{\eta_{st}^2}$$

where x and st indicate the sample and standard solution, respectively, ϕ is the quantum yield, F is the integrated area of the emission, A is the absorbance at the excitation wavelength, and η is the index of refraction of the solvents.

2.4. Computation details. Theoretical calculations were obtained using DFT performed using Gaussian 09.[9] The DFT calculations employed the B3LYP hybrid functional and the 6-311G+(d,p) basis set. All geometries were optimized in the ground state without solvent effects. Time-dependent (TD-DFT) calculations were performed on optimized geometries. The visual software used in this work was Avogadro 1.2.0 to analyze the output files performed in the calculations.[10]

3. Supplementary Analytical Data (Identification of intermediate 5a)

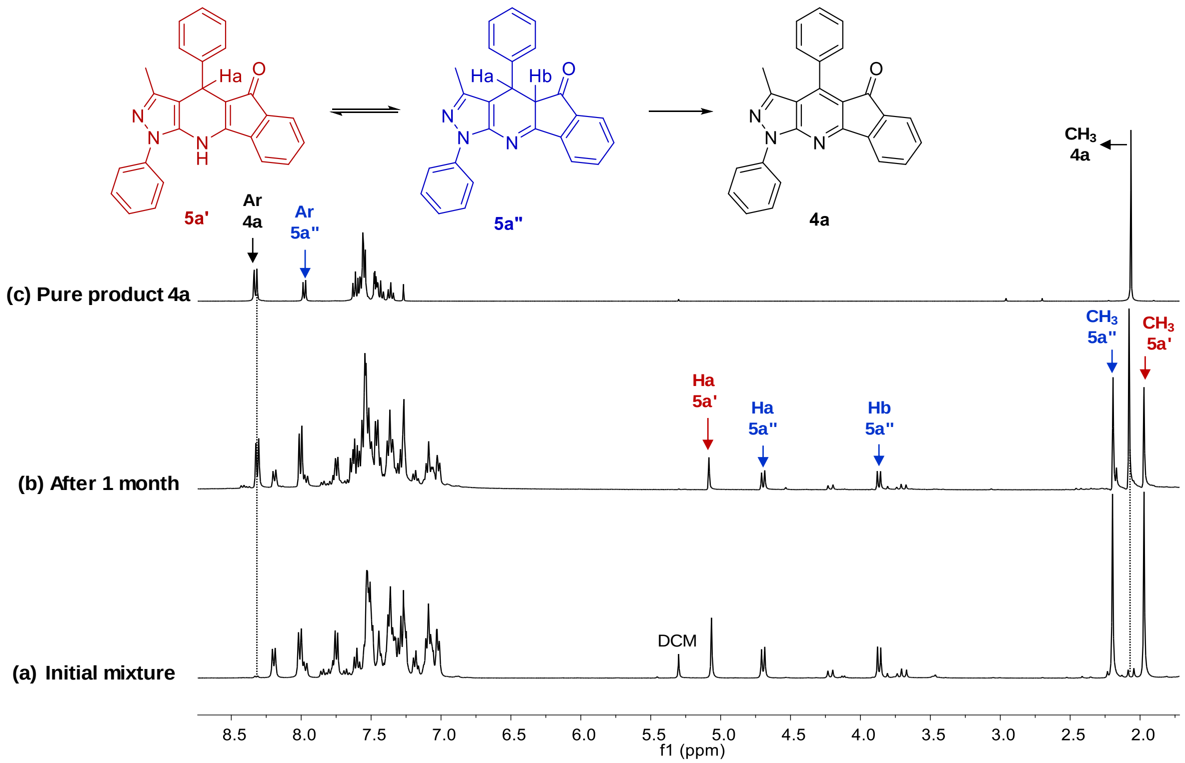

Fig. S 1. Identification by ^{1}H NMR ($CDCl_3$) of the intermediates **5a'-5a"** *versus* ^{1}H NMR of **4a**

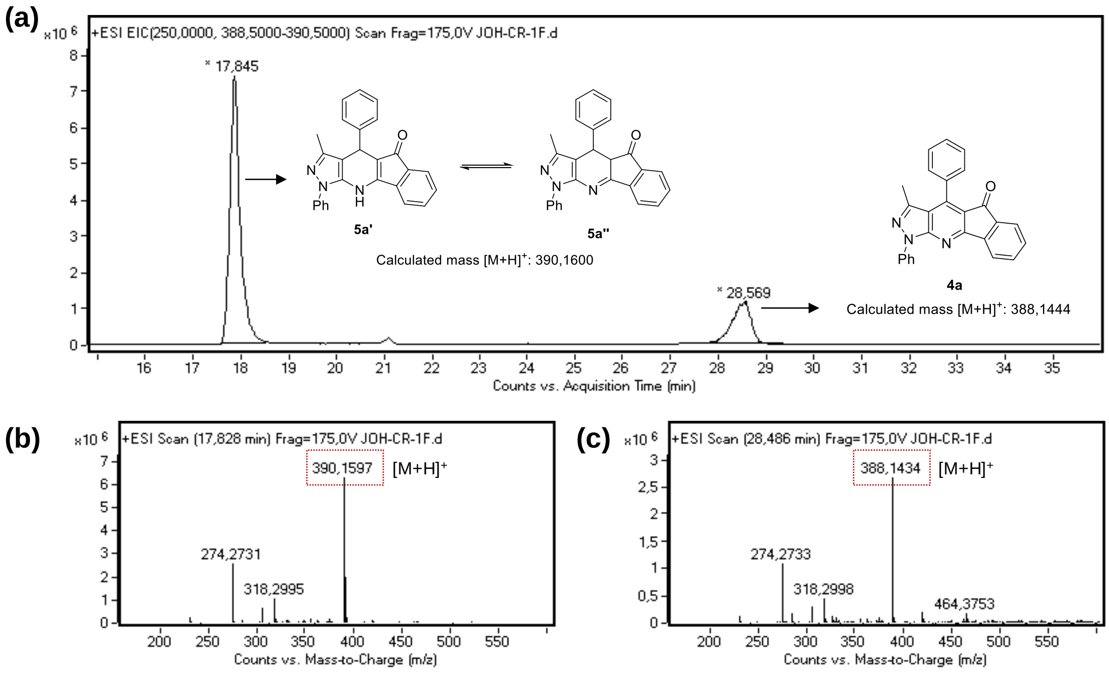

Fig. S2. HPLC-HRMS of the mixture of **5a'**, **5a"** and **4a**

4. Photophysical studies

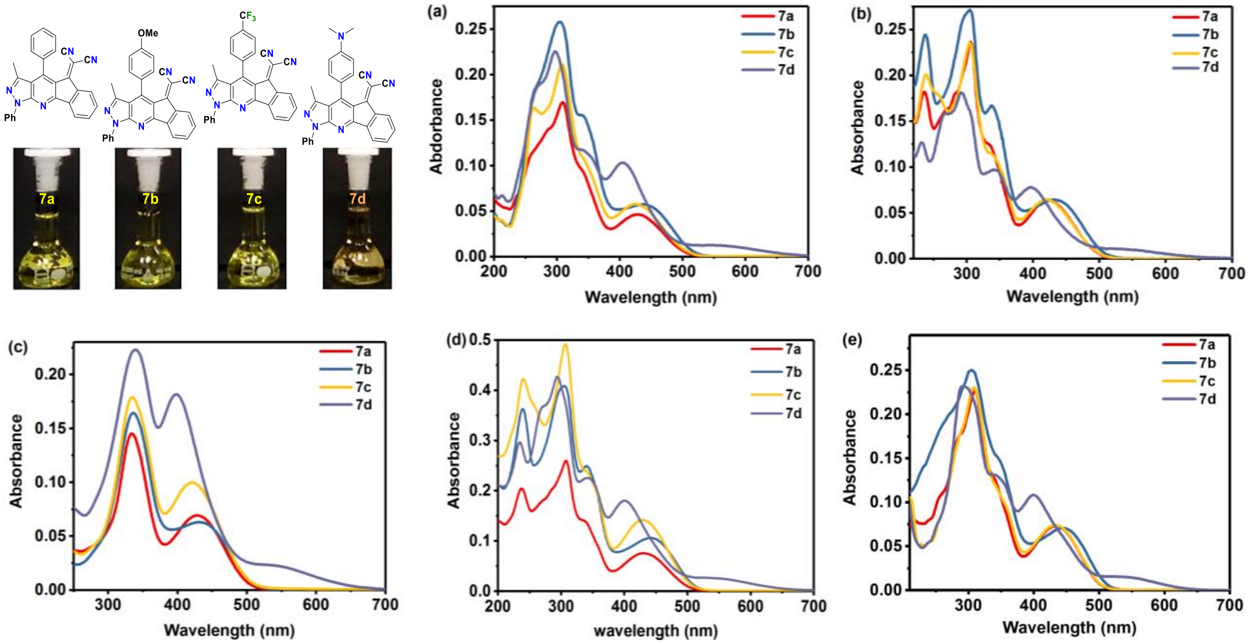

Fig. S3. UV-vis spectra of **7a-d** (10 μM). (a) DMF, (b) ACN, (c) acetone, (d) DCM, and (e) toluene. Photograph of compounds **7a-d** in DMSO under natural.

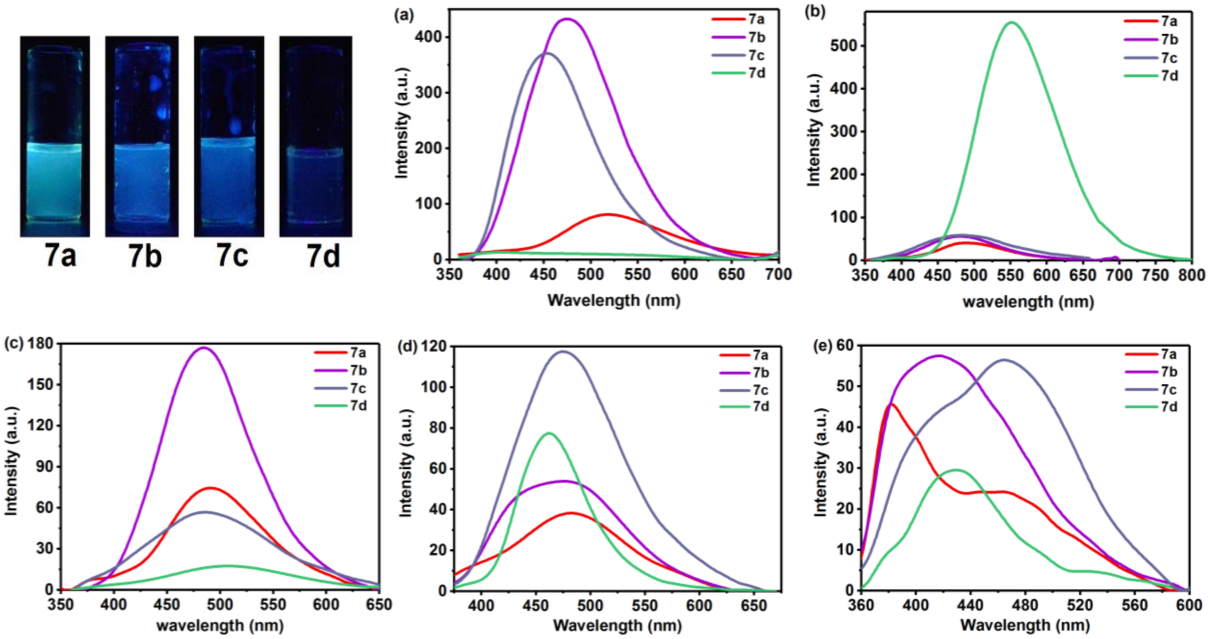

Fig. S4. Fluorescence spectra of **7a-d** (10 μM). (a) DMF, (b) ACN, (c) acetone, (d) DCM, and (e) toluene. Photograph under a hand-held UV lamp at long wavelength λ = 365 nm.

Table S1. Photophysical properties of compounds **7a-d**

Compound	Solvent[a]	λ_{ab} (nm)	ε (L mol^{-1} cm^{-1})	λ_{ex} (nm)	λ_{em} (nm)	Stokes shift (nm)	ϕ_F[b]
	DMSO	309	16970		519	210	0.030
	ACN	306	23633		490	184	0.011
**7a**	Acetona	334	14523	340	491	157	0.028
	DCM	307	25934		482	175	0.010
	Tolueno	310	22642		382	72	0.009
	DMSO	304	25793		475	171	0.133
	ACN	305	27142		482	177	0.013
**7b**	Acetona	336	16433	345	485	149	0.082
	DCM	305	40837		476	171	0.027
	Tolueno	305	25049		417	112	0.010
	DMSO	308	21140		453	145	0.114
	ACN	305	23535		486	181	0.015
**7c**	Acetona	335	17885	350	485	150	0.012
	DCM	307	49177		475	168	0.050
	Tolueno	308	23044		465	157	0.011
	DMSO	297	22544	350	406	109	0.002
	ACN	260	20342	345	550	290	0.181
**7d**	Acetona	339	22311		507	168	0.003
	DCM	294	42680	350	462	168	0.026
	Tolueno	291	23195		430	139	0.006

[a]50 μM. [b]Relative quantum yields were taken by using quinine sulfate as a reference (ϕ_F = 0.59 in 0.15 M $HClO_4$).

5. Computational calculations

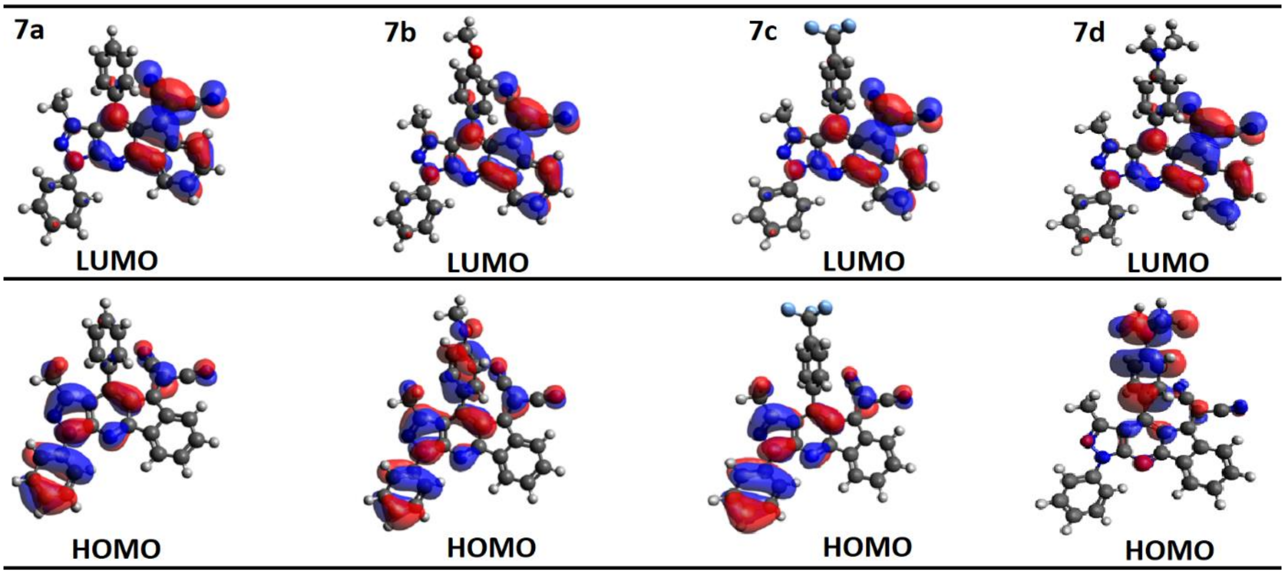

Fig. S5. Frontier Orbitals of compounds **7a-d**

Table S2. Theoretical data of **7a-d**.TD-DFT calculations at B3LYP/6-31G(*d*,*p*) theory level

Compound	Wavelength (nm)		Excitation energy (eV)	Oscillator strength (*f*)	Transitions
	Theoretical[a]	Experimental[b]			
**7a**	477	434	2.6922	0.6964	HOMO-1 → LUMO+1 (100%)
	368	------	3,648	0.0628	HOMO-4 → LUMO (22%)<br>HOMO→ LUMO+1 (30%)
	344	344	3.6019	0.2363	HOMO-5 → LUMO (33%)<br>HOMO-2 → LUMO (17%)
	295	310	4.1959	0.5933	HOMO-1 → LUMO+1 (43%)<br>HOMO-6→ LUMO (31%)
	291	285	4.2543	0.2476	HOMO-8 → LUMO (30%)<br>HOMO-4→ LUMO+1 (24%)<br>HOMO-2 → LUMO+1 (24%)
**7b**	498	--------	2.4875	0.0902	HOMO-1 → LUMO (35%)<br>HOMO→ LUMO (65%)
	464	--------	2.6707	0.0886	HOMO-2 → LUMO (36%)<br>HOMO-1→ LUMO (44%)<br>HOMO→ LUMO (20%)
	457	445	2.7156	0.0507	HOMO-2 → LUMO (49%)<br>HOMO-1→ LUMO (31%)<br>HOMO-→ LUMO (20%)
	362	--------	3.4225	0.0835	HOMO-5 → LUMO (39%)<br>HOMO-3→ LUMO (43%)<br>HOMO-→ LUMO+1 (18%)
	339	347	3.6538	0.2224	HOMO-5 → LUMO (18%)<br>HOMO-3→ LUMO (16%)<br>HOMO-1→ LUMO+1 (38%)
	300	305	4.1259	0.1098	HOMO-8 → LUMO (43%)<br>HOMO-3→ LUMO+1 (46%)
	294	300	4.2079	0.6188	HOMO-6 → LUMO (34%)<br>HOMO-2→ LUMO+1 (52%)
**7c**	480	436	2.5802	0.1984	HOMO → LUMO (100%)
	351	355	3.5284	0.1925	HOMO-4 → LUMO (44%)<br>HOMO-2→ LUMO (25%)
	312	307	3.9615	0.1059	HOMO-8 → LUMO (14%)<br>HOMO-7→ LUMO (22%)<br>HOMO-6 → LUMO (17%)<br>HOMO-1→ LUMO+1 (23%)
	298	285	4.1572	0.4470	HOMO-6 → LUMO (28%)<br>HOMO-1→ LUMO+1 (32%)
**7d**	644	--------	1.9259	0.0333	HOMO→ LUMO (100%)
	471	550	2.6337	0.1850	HOMO-2 → LUMO (24%)<br>HOMO-1→ LUMO (76%)
	416	400	2.9792	0.1479	HOMO→ LUMO+1 (100%)
	343	343	3.6133	0.2563	HOMO-5 → LUMO (31%)<br>HOMO-3→ LUMO (28%)
	297	290	4.1821	0.5251	HOMO-9 → LUMO (16%)<br>HOMO-6→ LUMO (28%)<br>HOMO-2 → LUMO+1 (56%)

[a] Data calculated on gas phase. [b] Experimental data in toluene

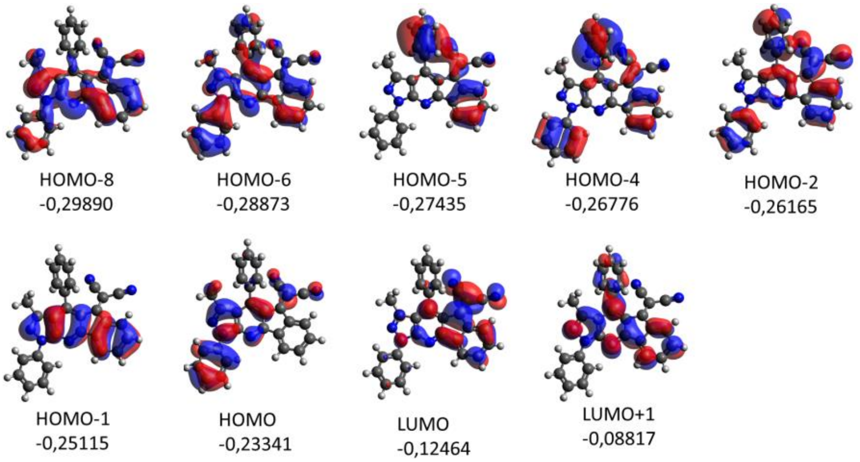

Fig. S6. Frontier molecular orbitals of compound **7a**

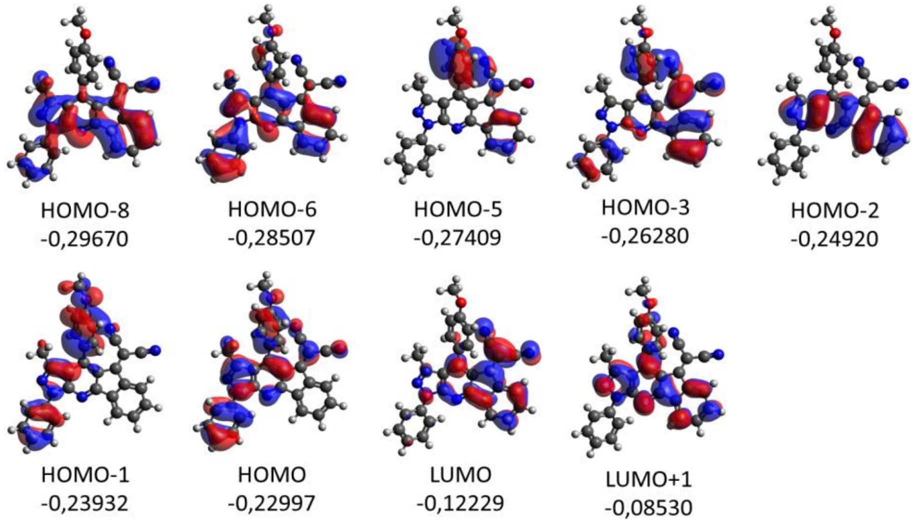

Fig. S7. Frontier molecular orbitals of compound **7b**

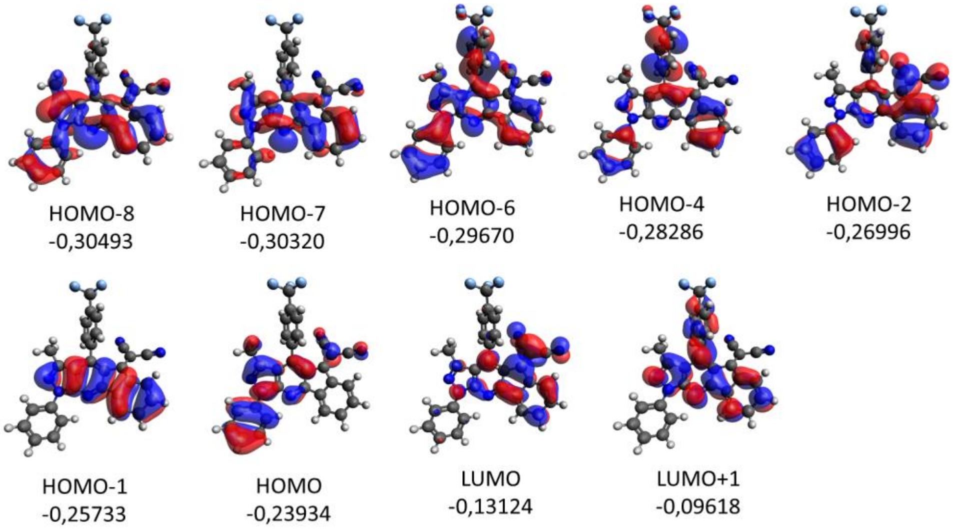

Fig. S8. Frontier molecular orbitals of compound **7c**

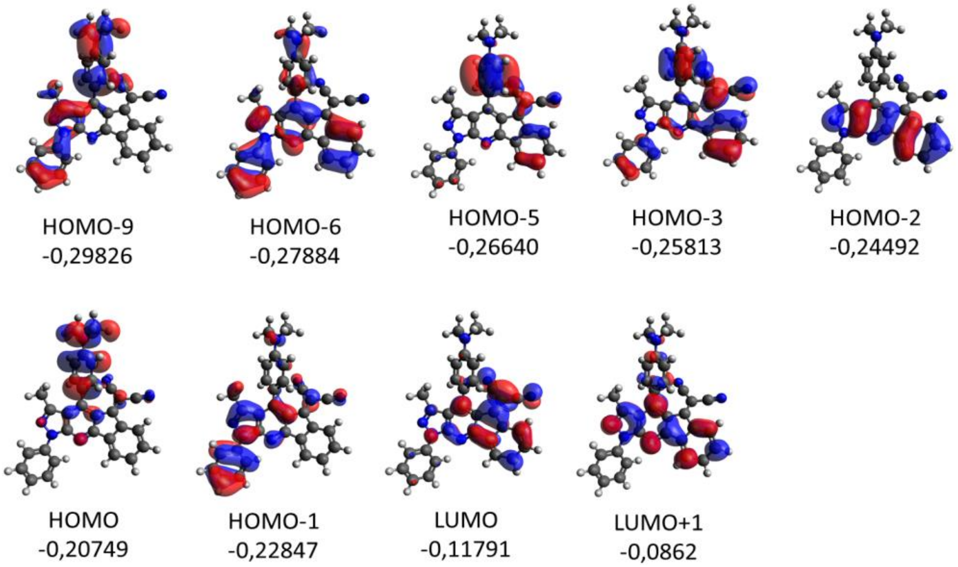

Fig. S9. Frontier molecular orbitals of compound **7d**

3.1 Coordinates of the optimized structure for **7a** calculated at the B3LYP/6-31+G(d, p) level theory

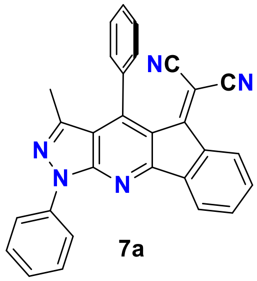

7a

SCF Energy: -1389.11581695 a. u.
Num. Imaginary Frequencies: 0
HOMO energy: -0.23473 eV
LUMO energy: -0.12464 eV

Atom	X	Y	Z	Atom	X	Y	Z
N	1.45067	1.05228	-0.06115	C	-1.83445	-3.10994	-0.64568
N	2.93430	-0.85397	-0.10439	H	-1.25158	-3.14857	-1.56033
N	2.81572	-2.21531	0.02142	C	0.28379	3.88625	0.38859
C	0.73524	-1.31264	-0.03590	H	1.36762	3.92419	0.35233
C	1.69947	-0.26744	-0.11266	C	1.13823	-3.94931	0.24177
C	-0.92496	0.39808	-0.07878	H	0.70276	-4.36325	-0.67265
C	0.16340	1.33793	-0.01690	H	0.40005	-4.07391	1.03851
C	-0.64523	-0.97957	-0.00566	H	2.03183	-4.52724	0.48791
C	-0.38103	2.68195	0.19209	C	-3.69689	-0.37531	-1.30600
C	4.22996	-0.26664	-0.14104	C	-0.48059	5.03090	0.64312
C	1.52897	-2.51266	0.07449	H	0.01230	5.98450	0.80679
C	-2.46838	-1.96681	1.38915	C	-3.55541	-4.04107	0.77777
H	-2.34449	-1.13953	2.08207	C	5.71659	1.53469	-0.75255
C	-1.67088	-2.03054	0.23438	H	5.86840	2.50928	-1.20706
C	-3.41449	0.82459	-0.58075	C	-1.87670	4.95288	0.69750
C	-1.79060	2.59313	0.22454	C	-2.54655	3.74129	0.48285
C	-2.17148	1.19402	-0.11203	H	-3.62665	3.71669	0.53297
C	-3.40051	-2.96977	1.66134	C	6.79950	0.83572	-0.21409
H	-4.00432	-2.91244	2.56215	H	7.79666	1.26431	-0.24273
C	5.31001	-0.97708	0.40023	C	6.58855	-0.42226	0.35767
H	5.13823	-1.95245	0.83787	H	7.42197	-0.97726	0.77809
C	4.43129	0.99071	-0.72558	N	-5.50273	2.36420	-0.47466
H	3.59583	1.53485	-1.14522	N	-4.00121	-1.26535	-1.99246
C	-4.54912	1.69716	-0.52359	H	-2.45743	5.84527	0.90946
C	-2.77383	-4.10471	-0.37847	H	-4.28696	-4.81645	0.98454
H	-2.90490	-4.92216	-1.08085				

3.2. Coordinates of the optimized structure for **7b** calculated at the B3LYP/6-31+G(d, p) level theory

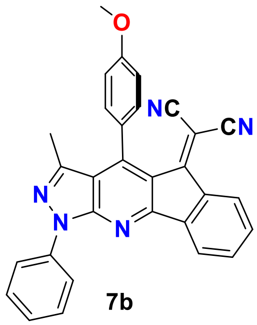

7b

SCF Energy: -1503.64623840 a. u.
Num. Imaginary Frequencies: 0
HOMO energy: -0.22997 eV
LUMO energy: -0.12229 eV

Atom	X	Y	Z	Atom	X	Y	Z
O	-5.56390	-2.83286	0.93302	H	-1.80263	-2.31211	-1.62545
N	2.14749	0.73038	-0.03815	C	2.00578	3.78487	0.47080
N	2.89209	-1.56622	-0.11815	H	3.03832	3.45157	0.45527
N	2.31501	-2.80779	-0.02855	C	0.14417	-3.86980	0.13214
C	0.66709	-1.24873	-0.08563	H	-0.37496	-4.10445	-0.80222
C	1.93086	-0.59364	-0.12463	H	-0.61812	-3.74332	0.90533
C	-0.30730	0.92656	-0.09768	H	0.78102	-4.71862	0.39127
C	1.03415	1.43721	0.00106	C	-3.14347	1.16161	-1.39847
C	-0.51895	-0.46546	-0.05627	C	1.67085	5.11730	0.73903
C	0.97573	2.88294	0.23336	H	2.45484	5.84260	0.93510
C	4.31058	-1.45516	-0.12639	C	-4.34638	-2.31007	0.62152
C	1.00307	-2.64890	0.00347	C	6.33313	-0.25502	-0.67293
C	-2.61700	-0.75081	1.27820	H	6.81597	0.62026	-1.09745
H	-2.23947	-0.01518	1.98252	C	0.33089	5.51959	0.76557
C	-1.84638	-1.09571	0.15038	C	-0.70603	4.61290	0.50889
C	-2.48602	2.18466	-0.64517	H	-1.73091	4.95734	0.53787
C	-0.38007	3.28026	0.23719	C	7.10334	-1.29471	-0.14666
C	-1.20609	2.09976	-0.13822	H	8.18722	-1.23167	-0.15461
C	-3.84405	-1.35345	1.51877	C	6.46591	-2.41868	0.38653
H	-4.43405	-1.09661	2.39233	H	7.05250	-3.23494	0.79769
C	5.07426	-2.50461	0.40264	C	-6.15981	-3.76465	0.03417
H	4.57246	-3.37310	0.81043	H	-6.31386	-3.31782	-0.95508
C	4.93909	-0.32800	-0.67204	H	-7.12477	-4.02088	0.47225
H	4.34683	0.47910	-1.08189	H	-5.55064	-4.67193	-0.06075
C	-3.25228	3.39404	-0.60163	N	-3.91899	4.34858	-0.56659
C	-3.59594	-2.66011	-0.50877	N	-3.72040	0.43991	-2.10736
H	-3.96967	-3.37569	-1.23072	H	0.08377	6.55287	0.98880
C	-2.35729	-2.05728	-0.72814				

3.3. Coordinates of the optimized structure for **7c** calculated at the B3LYP/6-31+G(d, p) level theory

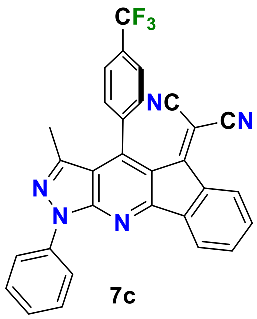

7c

SCF Energy: -1726.17280429a. u.
Num. Imaginary Frequencies: 0
HOMO energy: -0.23934 eV
LUMO energy: -0.13124 eV

Atom	X	Y	Z	Atom	X	Y	Z
N	2.63874	0.49576	-0.01517	H	3.95812	3.00640	0.60623
N	2.98752	-1.89033	-0.15076	C	-0.11895	-3.69977	-0.11752
N	2.20502	-3.01669	-0.13722	H	-0.64619	-3.78399	-1.07254
C	0.84973	-1.19928	-0.19616	H	-0.86788	-3.49651	0.65282
C	2.20726	-0.76868	-0.15778	H	0.35745	-4.65925	0.09508
C	0.25782	1.11204	-0.16780	C	-2.43973	1.87336	-1.54294
C	1.66198	1.38196	0.00316	C	2.88199	4.86931	0.91699
C	-0.18235	-0.22389	-0.18933	H	3.76903	5.44273	1.16867
C	1.83916	2.80675	0.29413	C	-4.25957	-1.47652	0.28883
C	4.40393	-2.02242	-0.09110	C	6.62353	-1.17327	-0.51365
C	0.93852	-2.63892	-0.15308	H	7.26784	-0.38608	-0.89381
C	-2.33419	-0.25222	1.07950	C	1.62920	5.49289	0.91824
H	-1.86228	0.36697	1.83619	C	0.46462	4.78685	0.59006
C	-1.60664	-0.63875	-0.05748	H	-0.48739	5.30011	0.60217
C	-1.65446	2.74365	-0.72427	C	7.17966	-2.33724	0.02206
C	0.57097	3.42903	0.27122	H	8.25765	-2.45888	0.06582
C	-0.42686	2.42391	-0.18410	C	6.33656	-3.34589	0.49712
C	-3.65148	-0.67184	1.25674	H	6.75618	-4.25638	0.91444
C	4.95124	-3.19494	0.44654	N	-2.70926	5.11218	-0.58609
H	4.29058	-3.97256	0.80898	N	-3.09064	1.26880	-2.29577
C	5.23886	-1.00914	-0.57948	H	1.55199	6.54413	1.17779
H	4.81231	-0.10725	-0.99777	H	-4.02988	-2.45924	-1.61669
C	-2.21081	4.06135	-0.64668	H	-4.20258	-0.37184	2.14141
C	-3.54975	-1.85730	-0.85327	C	-5.66492	-1.97575	0.50370
C	-2.23108	-1.44307	-1.02044	F	-6.31555	-2.20143	-0.66169
H	-1.69564	-1.71485	-1.92366	F	-6.41453	-1.10589	1.22323
C	2.99703	3.51008	0.60312	F	-5.67617	-3.15439	1.18869

3.4. Coordinates of the optimized structure for **7d** calculated at the B3LYP/6-31+G(d, p) level theory

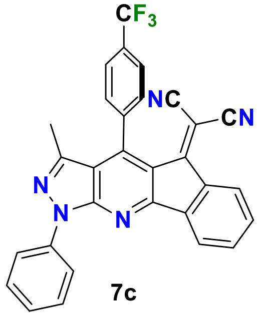

SCF Energy: -1523.09554001 a. u.
Num. Imaginary Frequencies: 0
HOMO energy: -0.20749 eV
LUMO energy: -0.11791 eV

Atom	X	Y	Z	Atom	X	Y	Z
N	2.42350	0.61107	-0.01552	C	0.06385	-3.82151	0.00543
N	2.98590	-1.73488	-0.12002	H	-0.43821	-4.00705	-0.94904
N	2.31170	-2.92852	-0.07156	H	-0.71408	-3.64737	0.75324
C	0.79189	-1.24355	-0.15041	H	0.62687	-4.71819	0.27444
C	2.10356	-0.68968	-0.13753	C	-2.76231	1.48088	-1.53558
C	-0.00441	1.00354	-0.13853	C	2.27363	5.01067	0.82449
C	1.36900	1.40340	0.00794	H	3.10619	5.66809	1.05661
C	-0.33121	-0.36875	-0.13230	C	-4.35424	-1.89426	0.41893
C	1.41934	2.84608	0.26191	C	6.53601	-0.68557	-0.52432
C	4.40764	-1.73496	-0.07689	H	7.10022	0.15793	-0.91129
C	1.01560	-2.66710	-0.07676	C	0.96986	5.51857	0.81572
C	-2.50453	-0.45211	1.10261	C	-0.12711	4.70151	0.51118
H	-2.10515	0.27010	1.80925	H	-1.12170	5.12666	0.51204
C	-1.70818	-0.88368	0.02791	C	7.20402	-1.79260	0.00430
C	-2.05287	2.43957	-0.74533	H	8.28914	-1.81411	0.03578
C	0.10027	3.35092	0.22871	C	6.46237	-2.87402	0.48851
C	-0.80379	2.24450	-0.19231	H	6.96861	-3.74188	0.90092
C	-3.78844	-0.94304	1.30456	N	-3.31040	4.71023	-0.68466
H	-4.35079	-0.58046	2.15571	N	-3.37238	0.82029	-2.27582
C	5.06858	-2.85141	0.45378	H	0.79868	6.56527	1.04764
H	4.48654	-3.68622	0.82352	N	-5.62777	-2.39295	0.61388
C	5.14143	-0.64951	-0.57391	C	-6.48526	-1.80384	1.63232
H	4.62853	0.20963	-0.98476	H	-6.69844	-0.74155	1.43956
C	-2.72027	3.70603	-0.71176	H	-7.43222	-2.34421	1.65638
C	-3.55505	-2.31277	-0.67410	H	-6.03176	-1.88819	2.62655
H	-3.94747	-3.00383	-1.40905	C	-6.23972	-3.22897	-0.40970
C	-2.26858	-1.82462	-0.85006	H	-6.35954	-2.70075	-1.36733
H	-1.70580	-2.14464	-1.72116	H	-5.64511	-4.13231	-0.58739
C	2.50970	3.65929	0.54540	H	-7.22537	-3.54600	-0.06717
H	3.51233	3.24440	0.55627				

6. Fluorescent chemosensors 7a-d for detection of cyanide

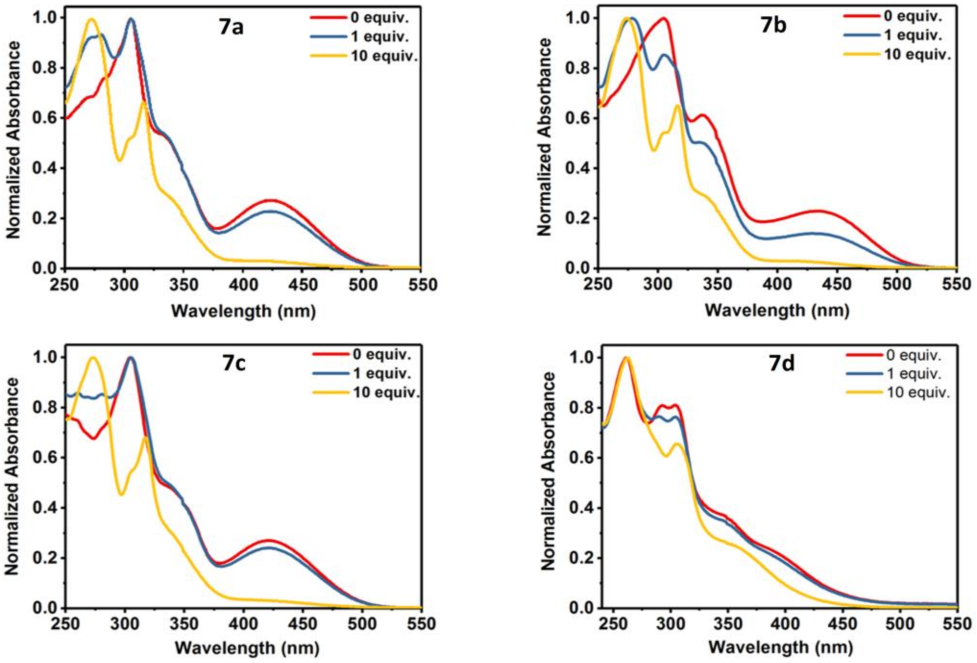

Fig. S10. Normalized UV-vis spectra of 50 μM acetonitrile solutions of **7a-d** with different equiv of CN^-

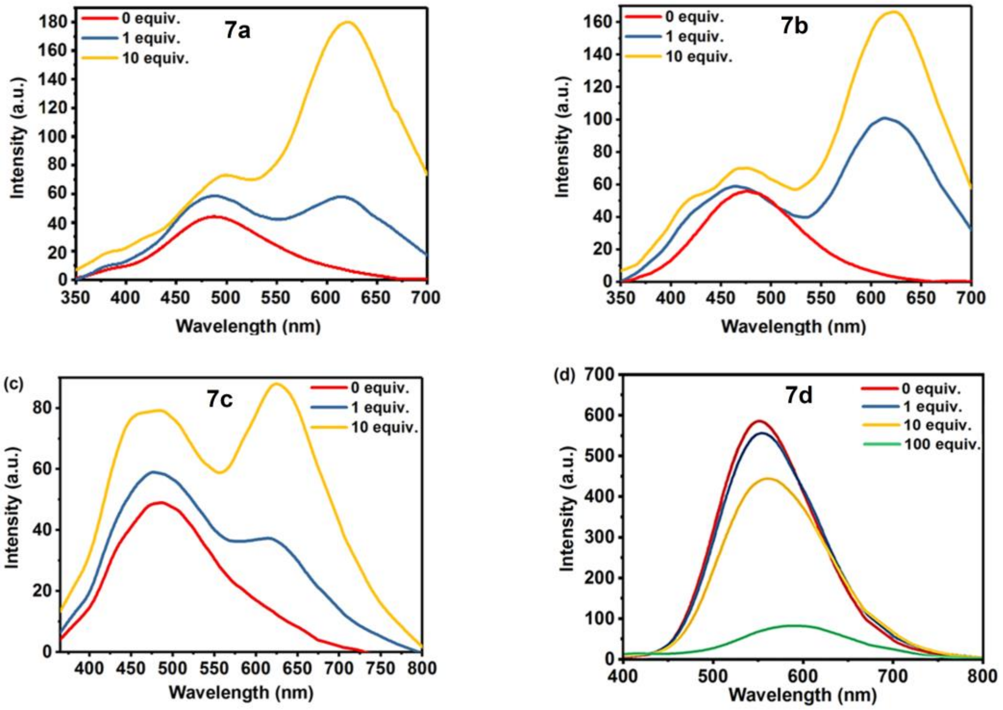

Fig. S11. Fluorescence spectra of 50 μM acetonitrile solutions of **7a-d** with different equiv of CN^-

Table S3. Photophysical properties of compounds **7a-d** with different equivalents of CN^-

**Compound**[a]	**Equiv CN^-**	**λ_{ab} (nm)**	**λ_{ex} (nm)**	**λ_{em} (nm)**	**Stokes shift (nm)**	**ϕ_F**[b]
	0	306		488	182	0.011
**7a**	1	306	340	489	183	0.026
	10	272		621	349	0.073
	0	305		480	175	0.013
**7b**	1	278	340	613	335	0.041
	10	274		623	349	0.079
	0	305		486	181	0.015
**7c**	1	305	345	477	172	0.027
	10	273		625	352	0.063
	0	260		550	290	0.181
**7d**	1	260	345	555	295	0.166
	10	260		560	300	0.161
	100	260		585	325	0.023

[a]50 μM. [b]Relative quantum yields were taken by using quinine sulfate as a reference (ϕ_F = 0.59 in 0.15 M $HClO_4$).

7. Copies of NMR spectra

^{1}H and ^{13}C{^{1}H} NMR spectra of 3-methyl-1,4-diphenylindeno[1,2-*b*]pyrazolo[4,3-*e*]pyridin-5(1*H*)-one (**4a**)

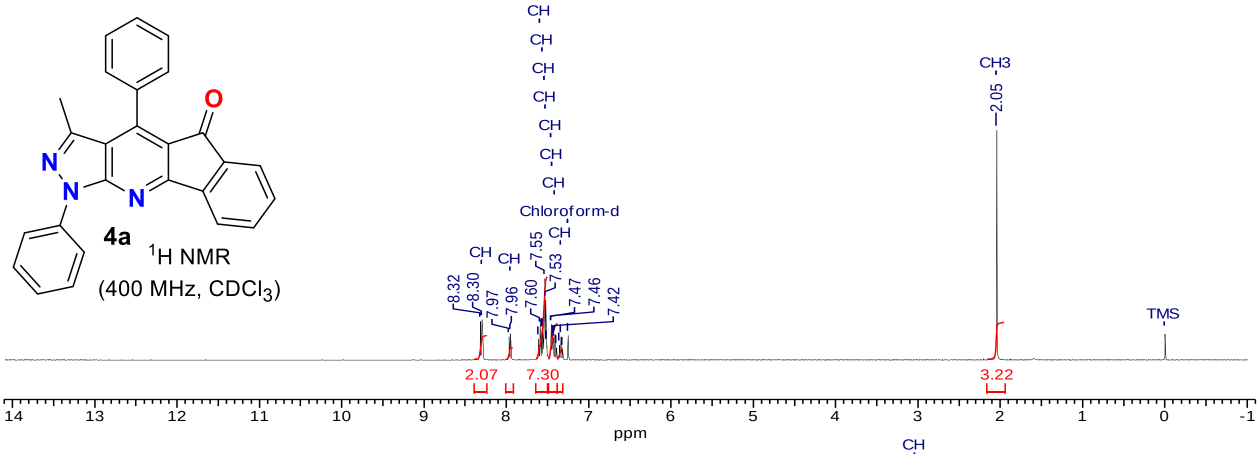

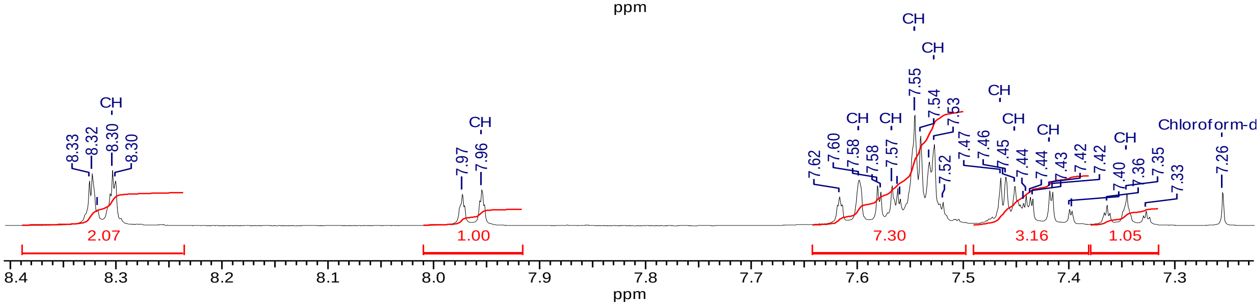

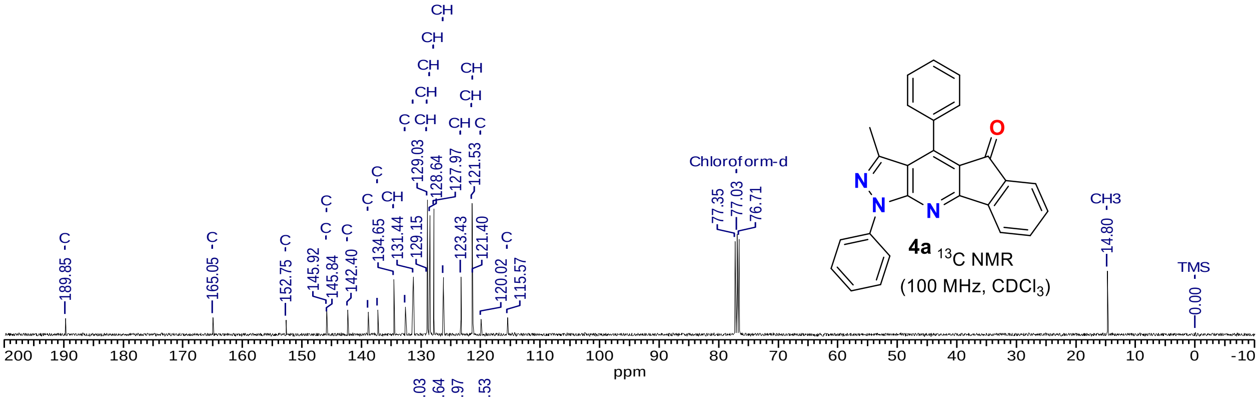

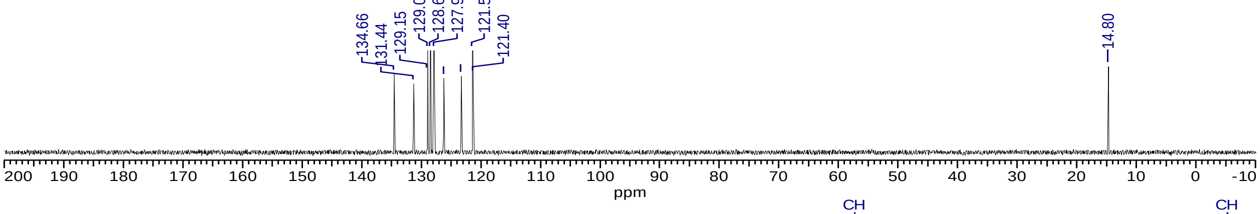

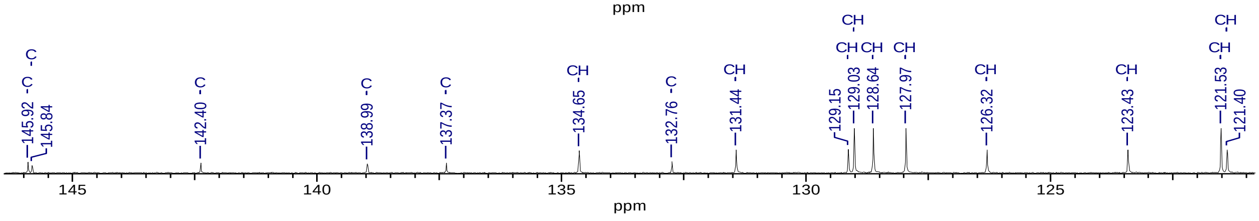

^{1}H and $^{13}C\{^{1}H\}$ NMR spectra of 4-(4-methoxyphenyl)-3-methyl-1-phenylindeno[1,2-*b*]pyrazolo[4,3-*e*]pyridin-5(1*H*)-one (**4b**)

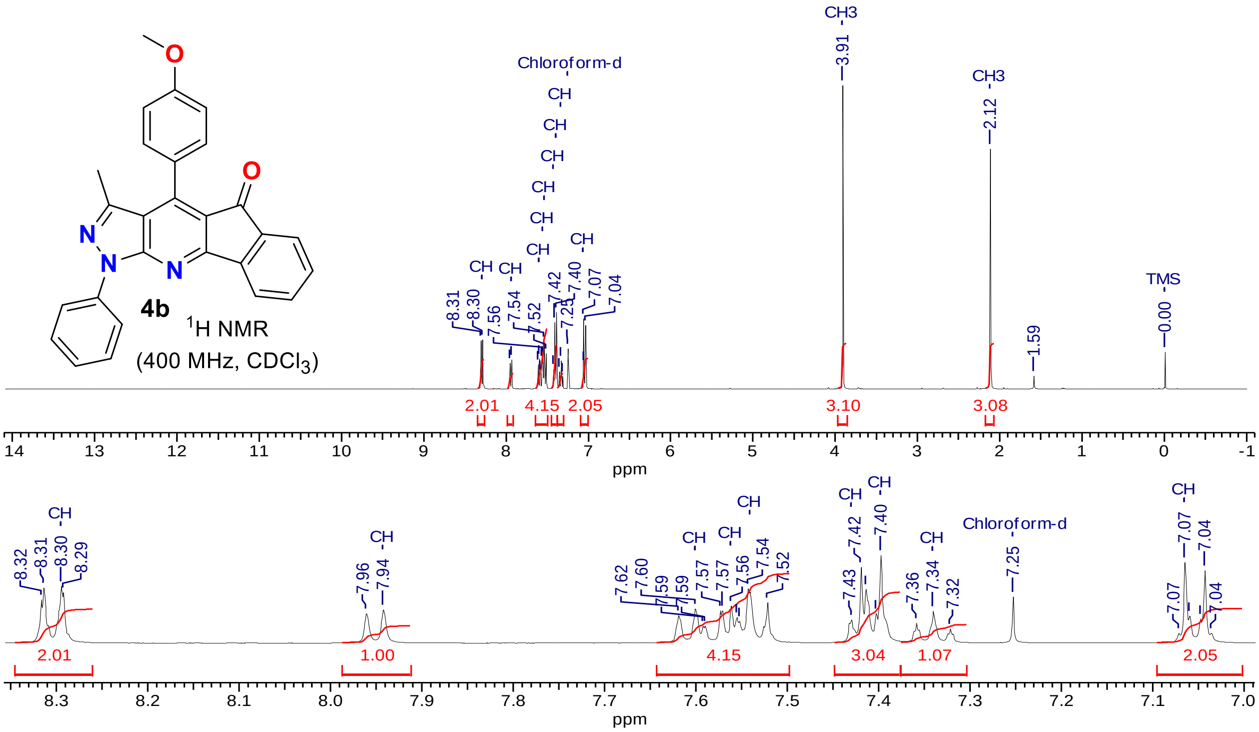

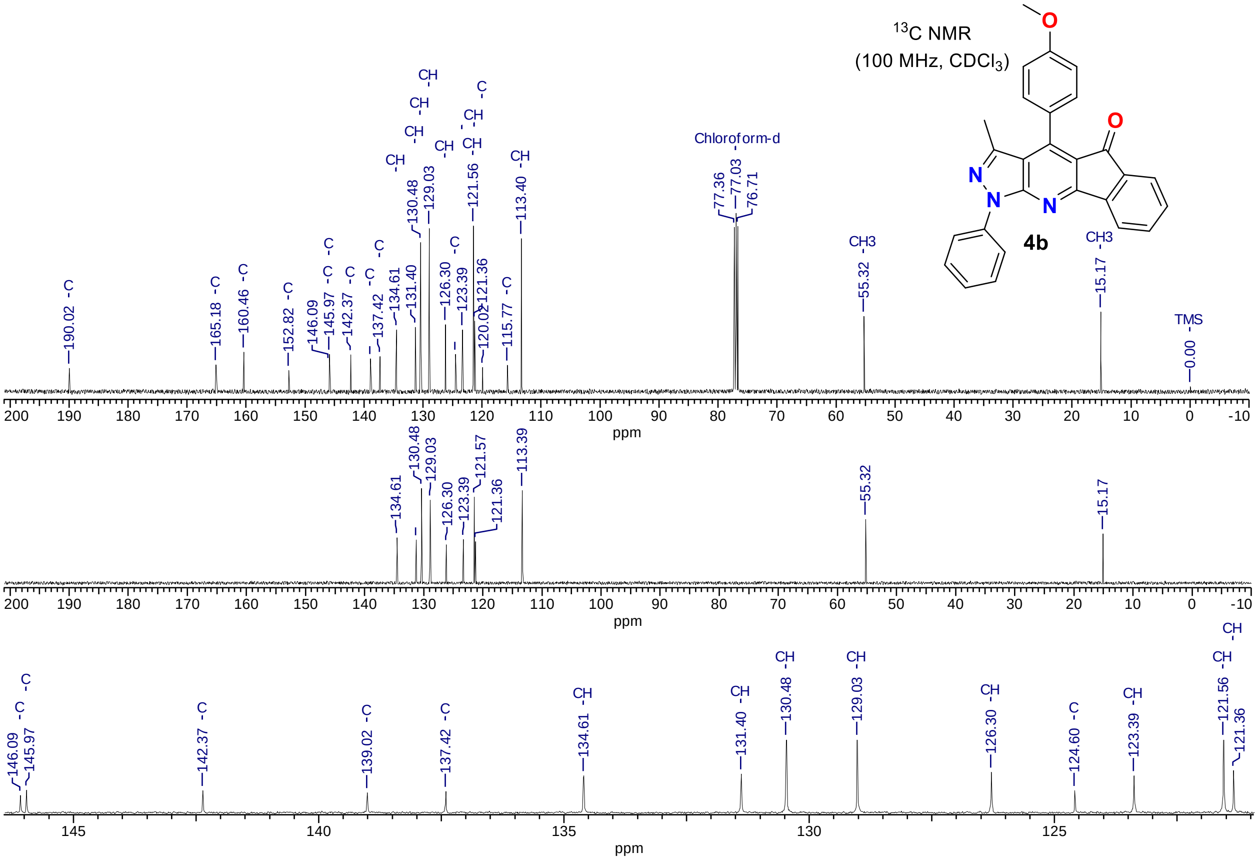

^{1}H and $^{13}C\{^{1}H\}$ NMR spectra of 3-methyl-1-phenyl-4-(4-(trifluoromethyl)phenyl)indeno[1,2-*b*]pyrazolo[4,3-*e*]pyridin-5(1*H*)-one (**4c**)

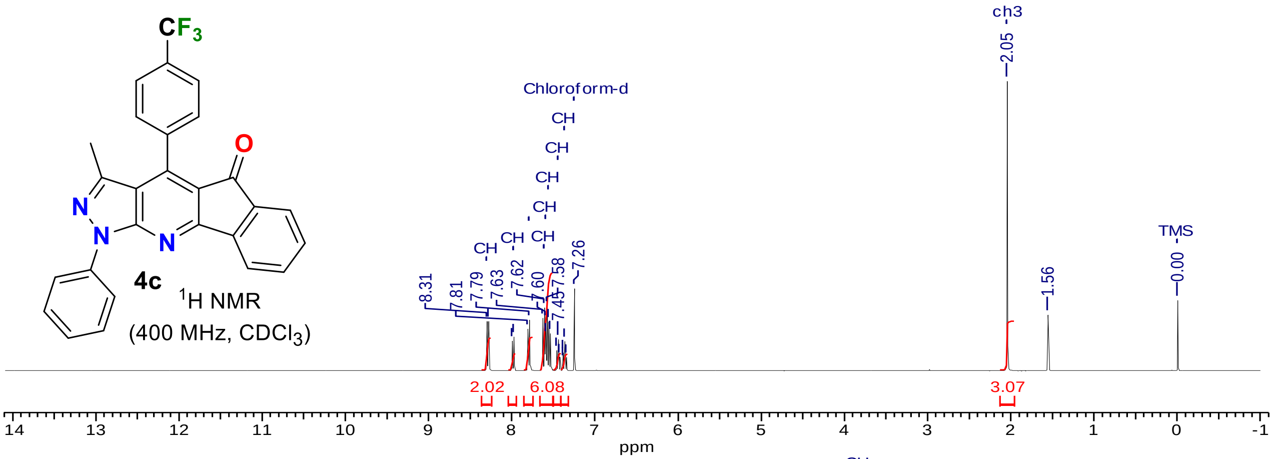

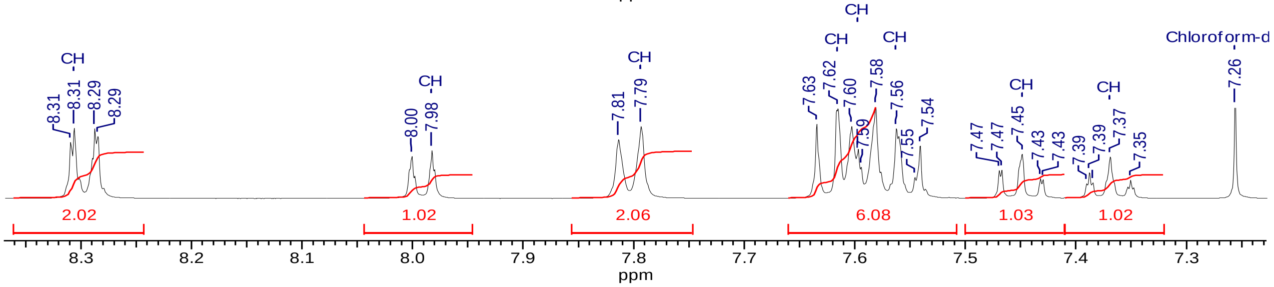

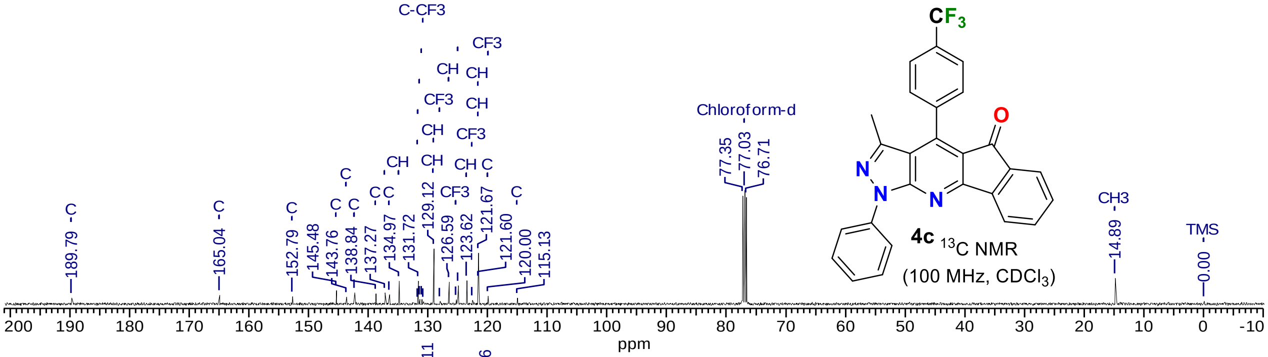

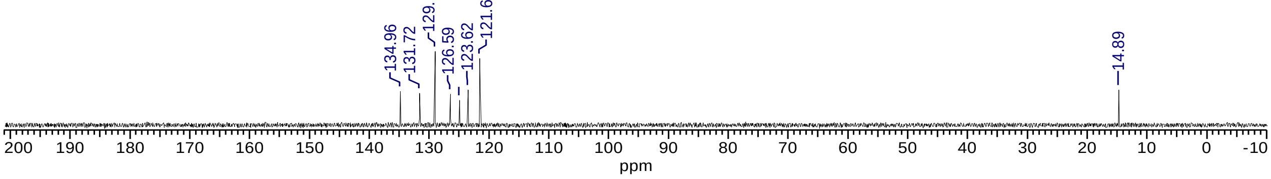

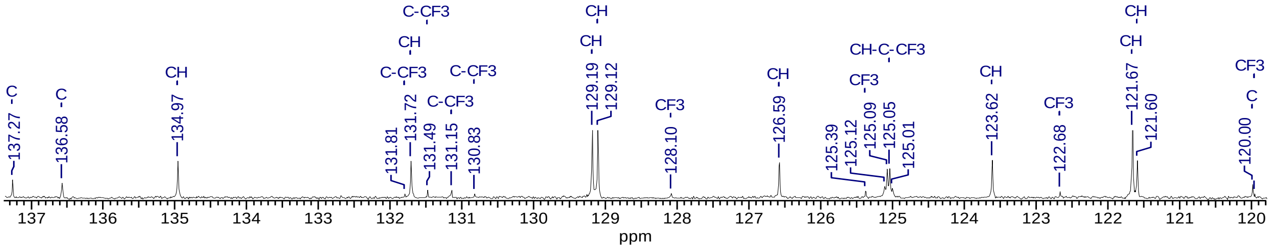

^{1}H and $^{13}C\{^{1}H\}$ NMR spectra of 4-(4-(dimethylamino)phenyl)-3-methyl-1-phenylindeno[1,2-*b*]pyrazolo[4,3-*e*]pyridin-5(1*H*)-one (**4d**)

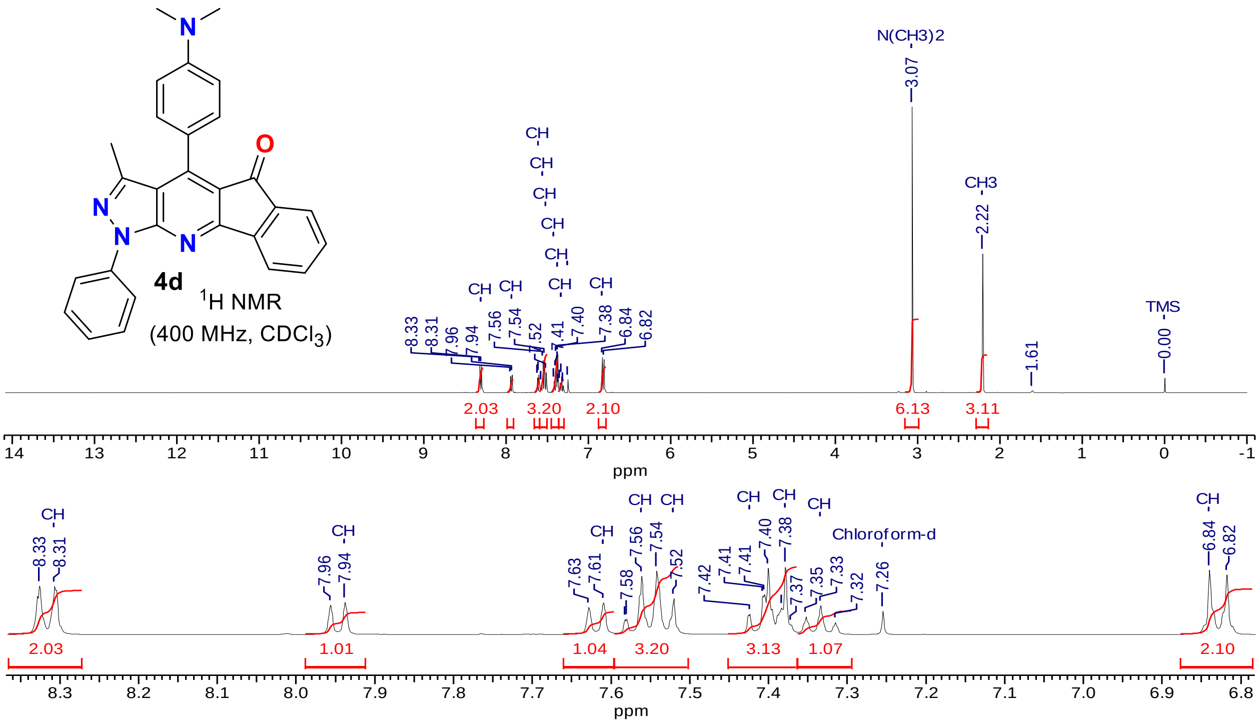

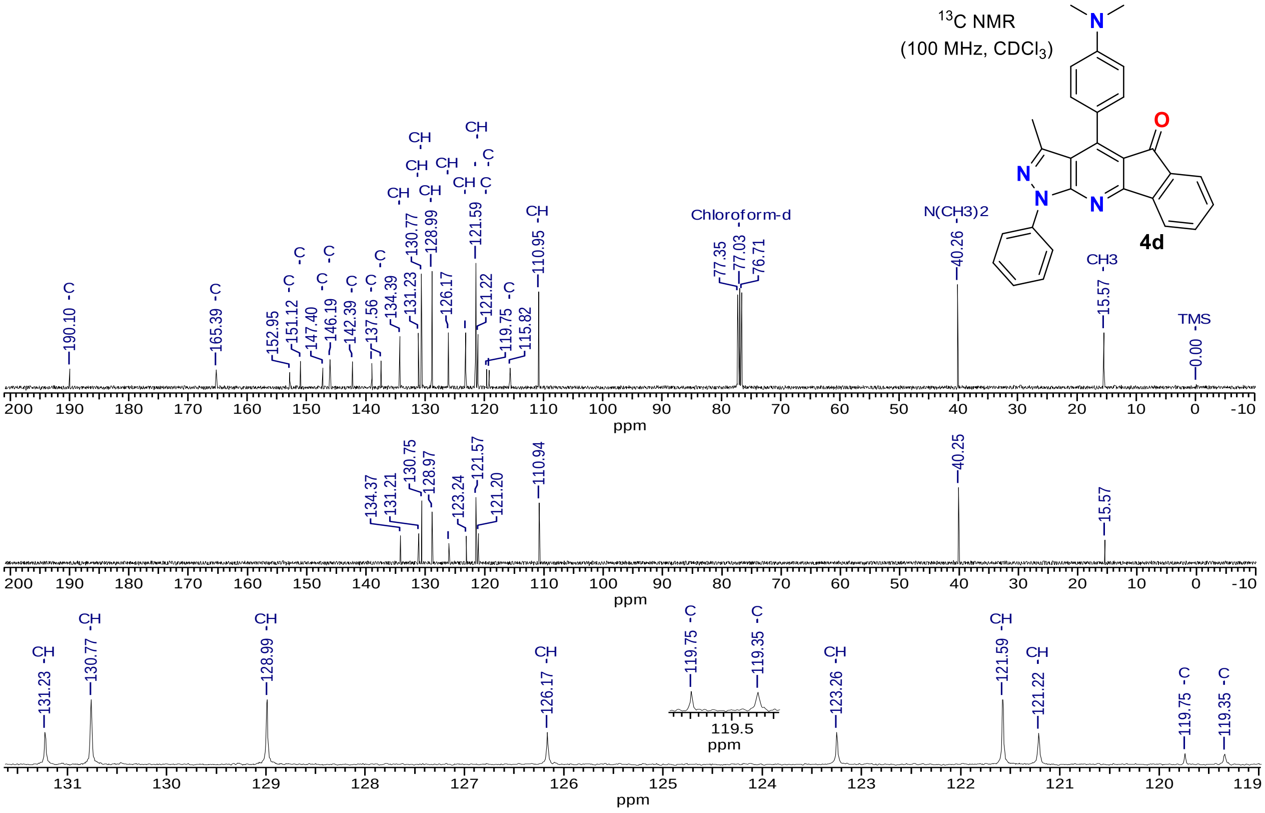

^{1}H and ^{13}C{^{1}H} NMR spectra of 3-methyl-1-phenyl-4-(*p*-tolyl)indeno[1,2-*b*]pyrazolo[4,3-*e*]pyridin-5(1*H*)-one (**4e**)

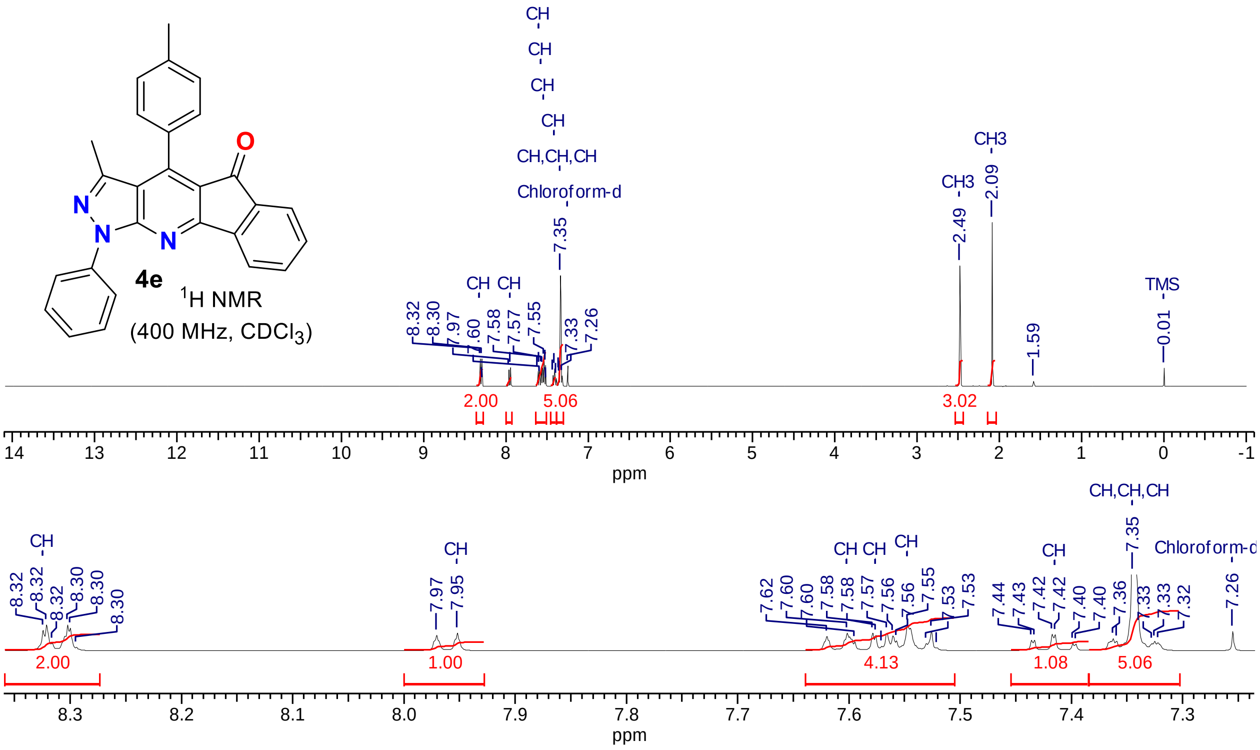

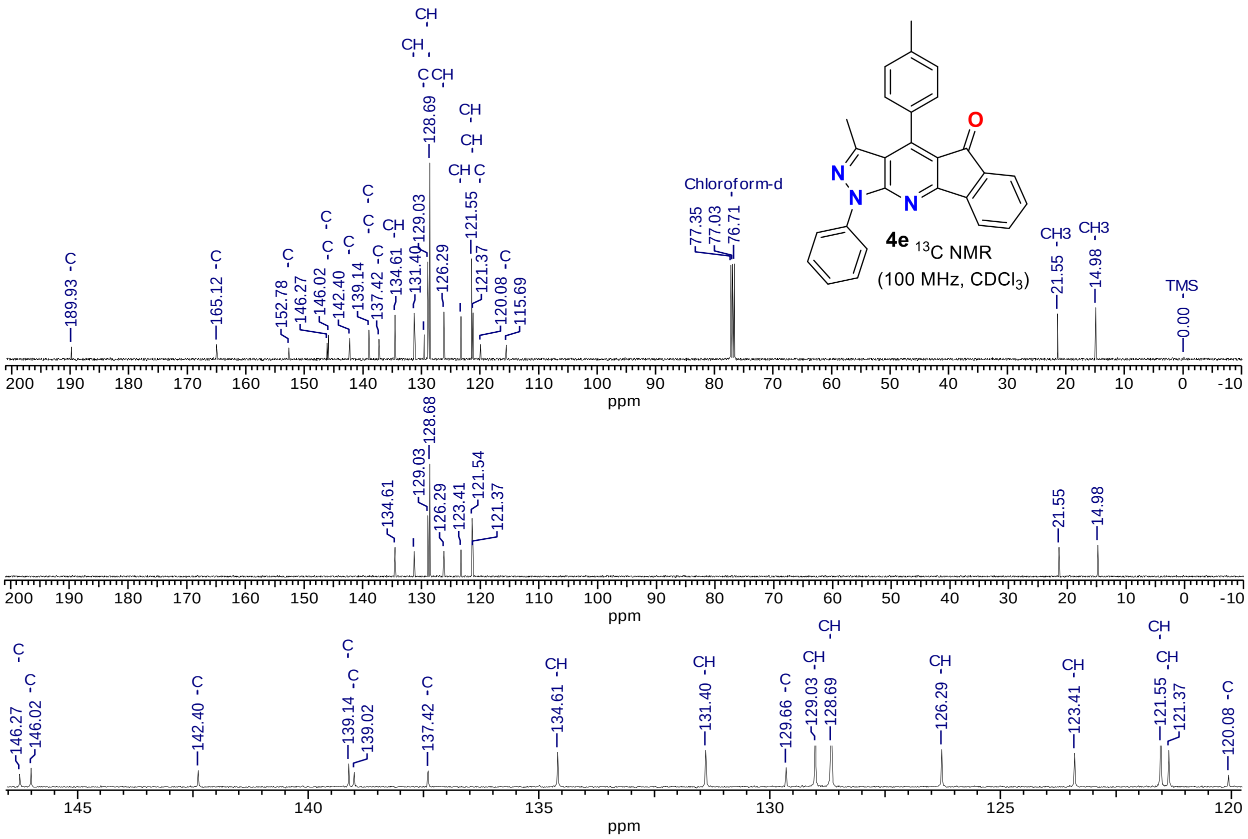

^{1}H and $^{13}C\{^{1}H\}$ NMR spectra of 4-(4-chlorophenyl)-3-methyl-1-phenylindeno[1,2-*b*]pyrazolo[4,3-*e*]pyridin-5(1*H*)-one (**4f**)

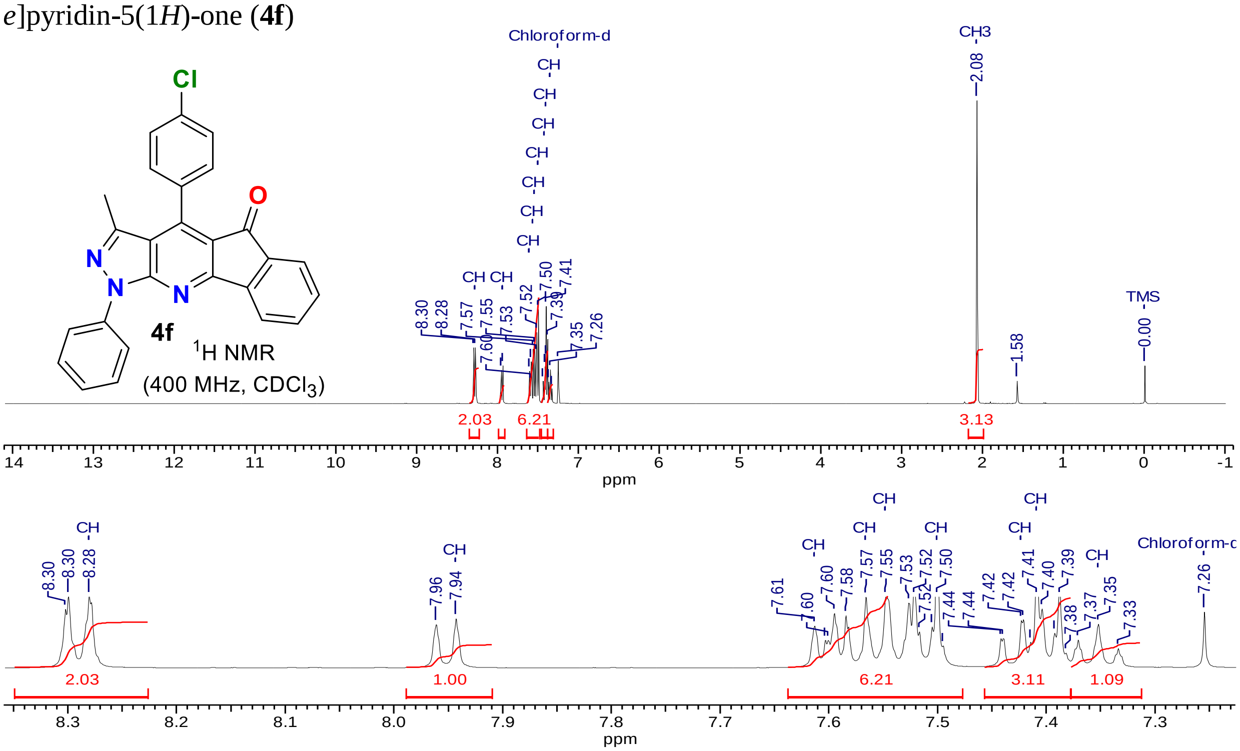

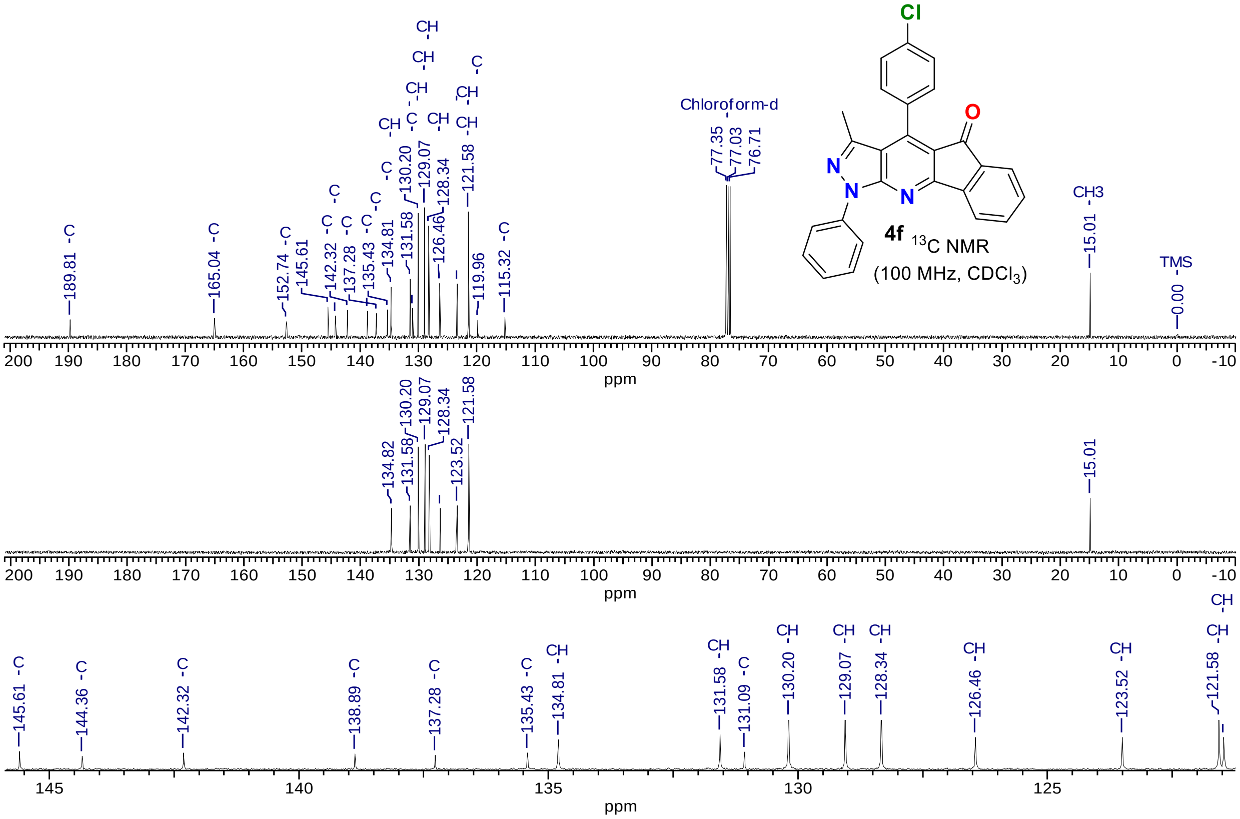

^{1}H and $^{13}C\{^{1}H\}$ NMR spectra of 4-(4-bromophenyl)-3-methyl-1-phenylindeno[1,2-*b*]pyrazolo[4,3-*e*]pyridin-5(1*H*)-one (**4g**)

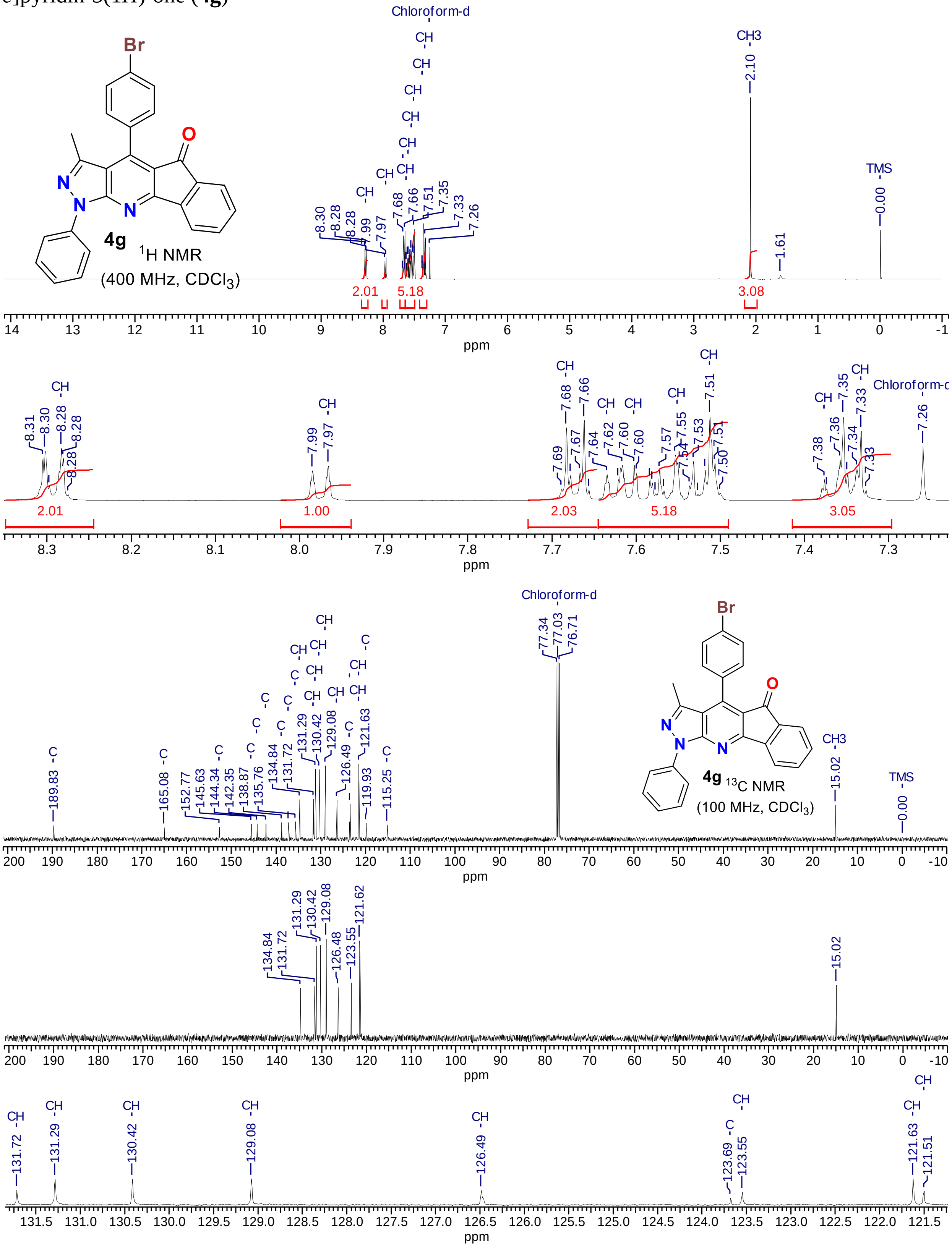

^{1}H and ^{13}C{^{1}H} NMR spectra of 3-methyl-4-(4-nitrophenyl)-1-phenylindeno[1,2-*b*]pyrazolo[4,3-*e*]pyridin-5(1*H*)-one (**4h**)

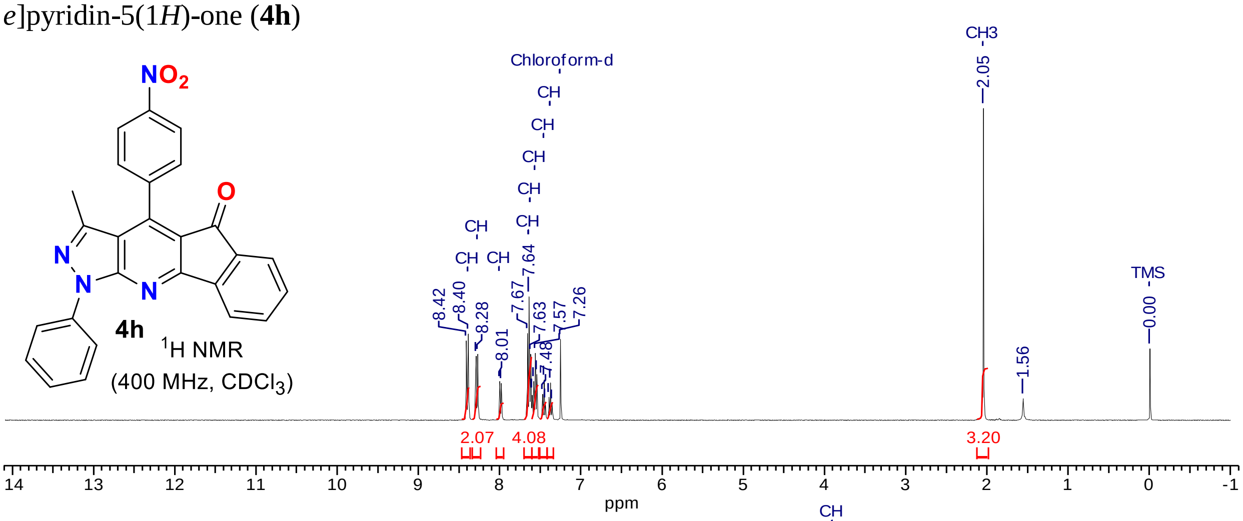

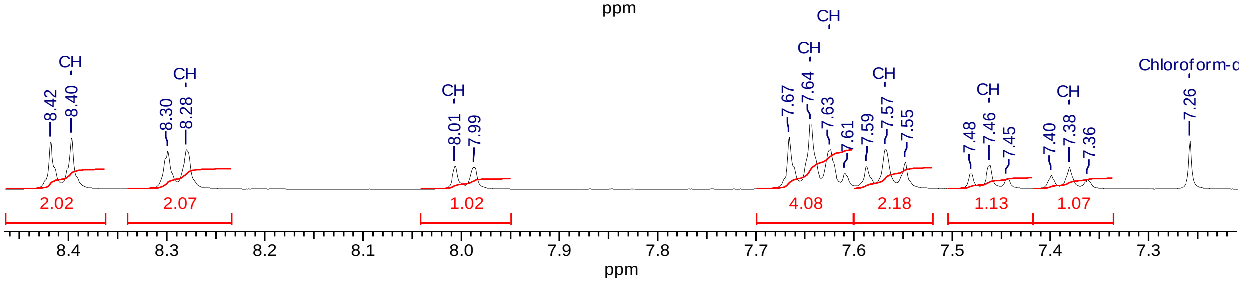

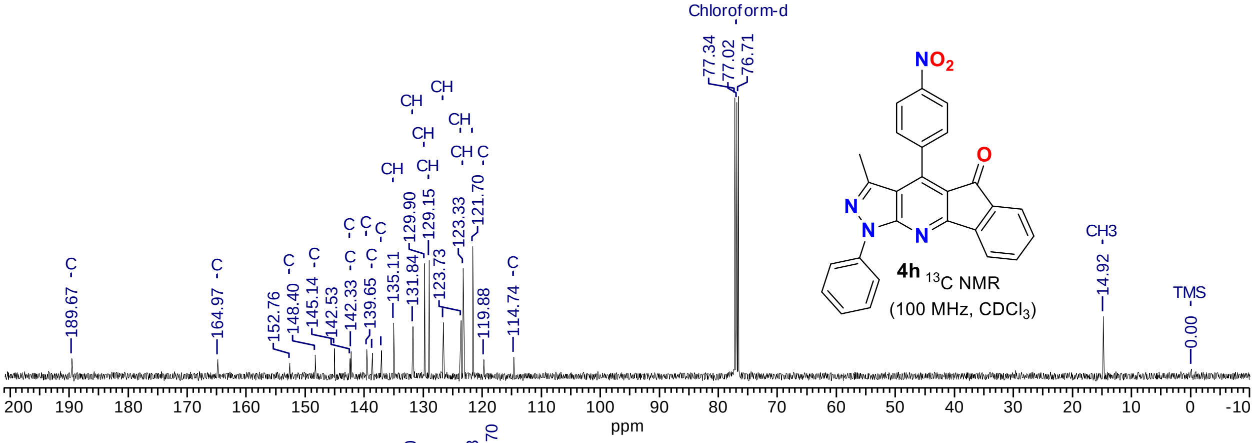

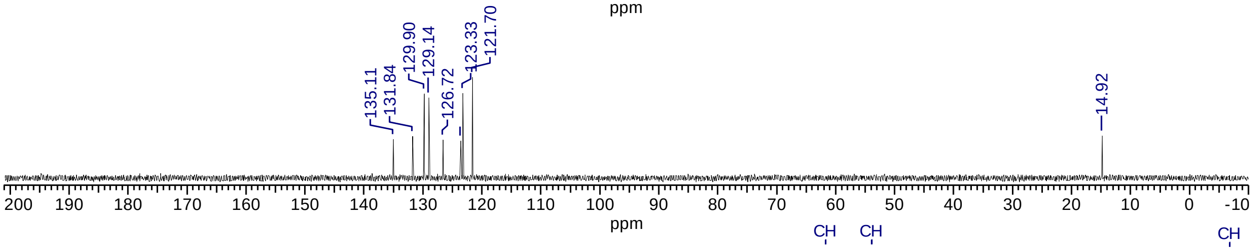

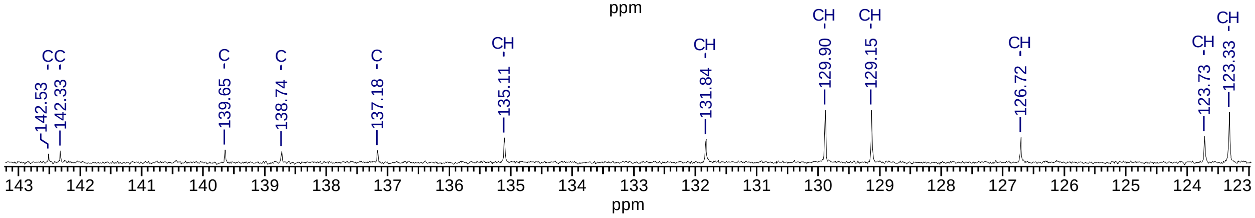

^{1}H and ^{13}C{^{1}H} NMR spectra of 4-(4-(dimethylamino)phenyl)-3-methyl-1-phenylindeno[1,2-*b*]pyrazolo[4,3-*e*]pyridin-5(1*H*)-one (**4i**)

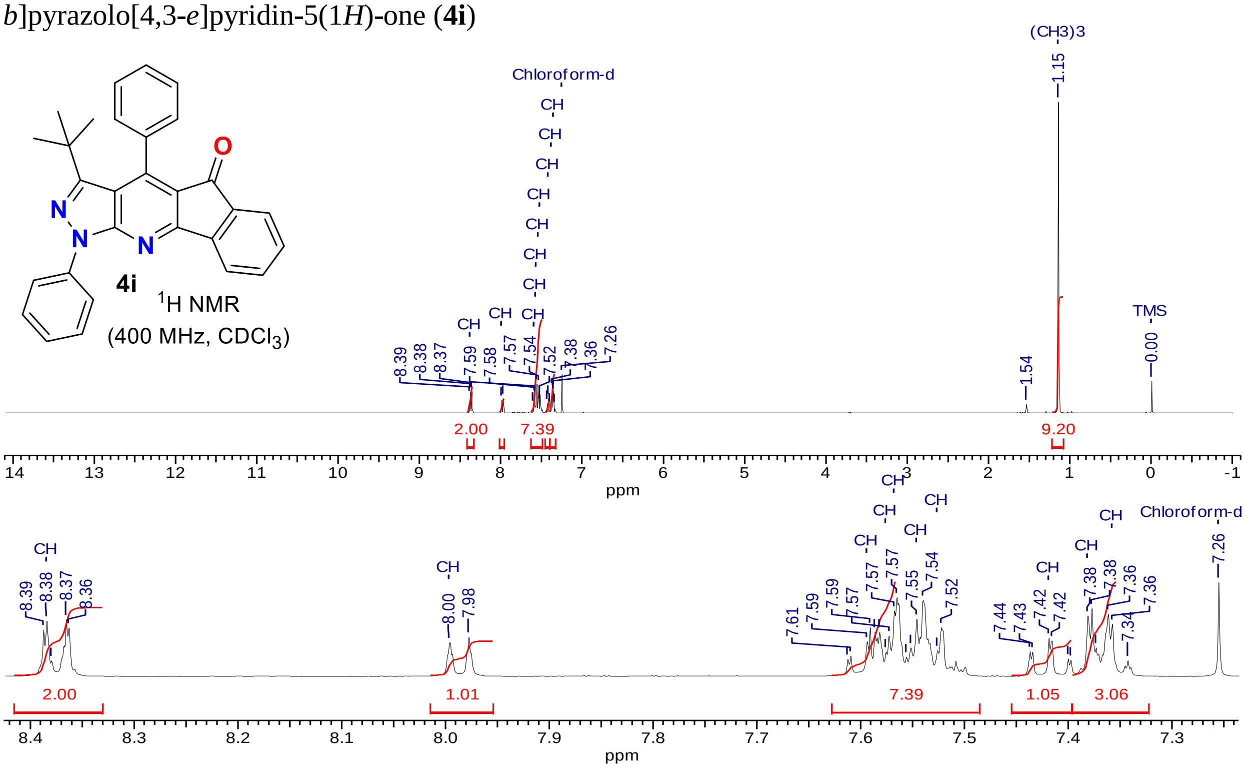

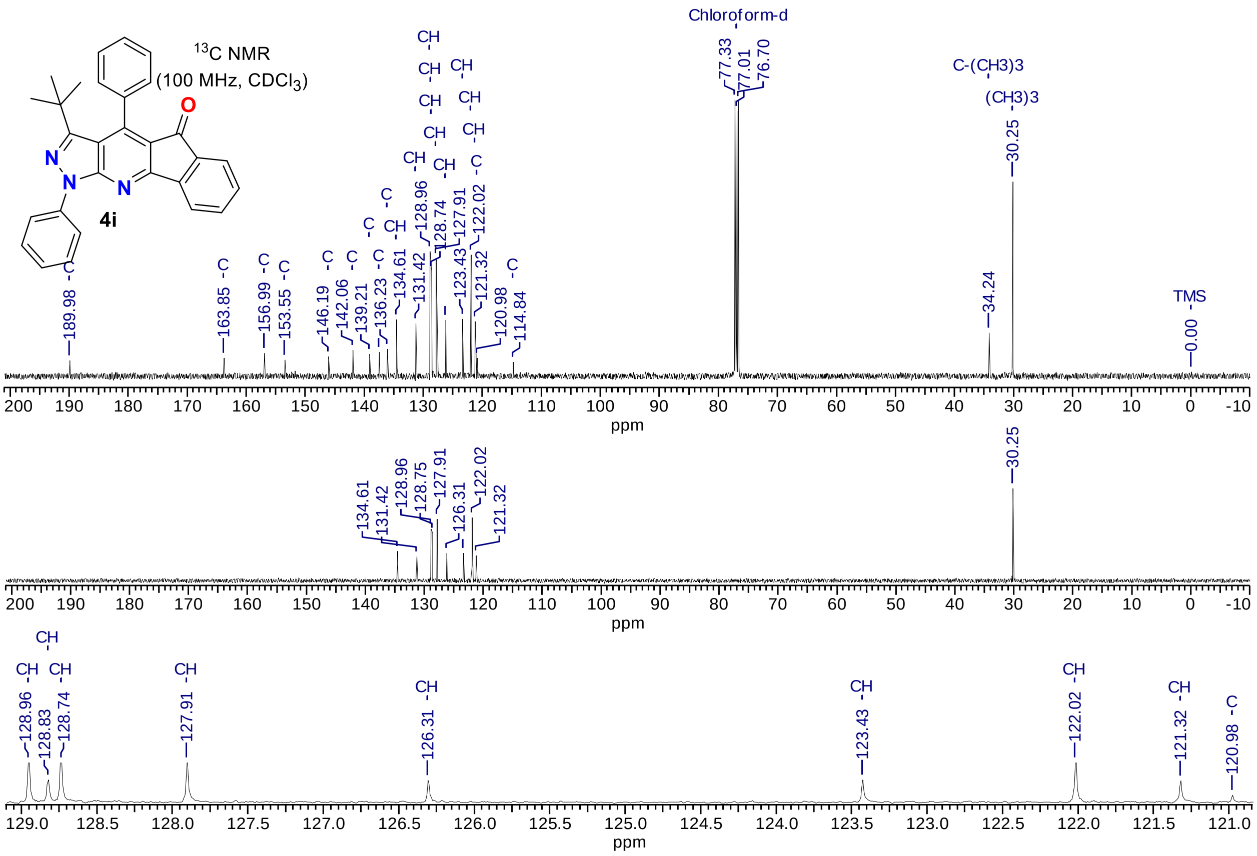

^{1}H and ^{13}C{^{1}H} NMR spectra of 3-(*tert*-butyl)-1-phenyl-4-(*p*-tolyl)indeno[1,2-*b*]pyrazolo[4,3-*e*]pyridin-5(1*H*)-one (**4j**)

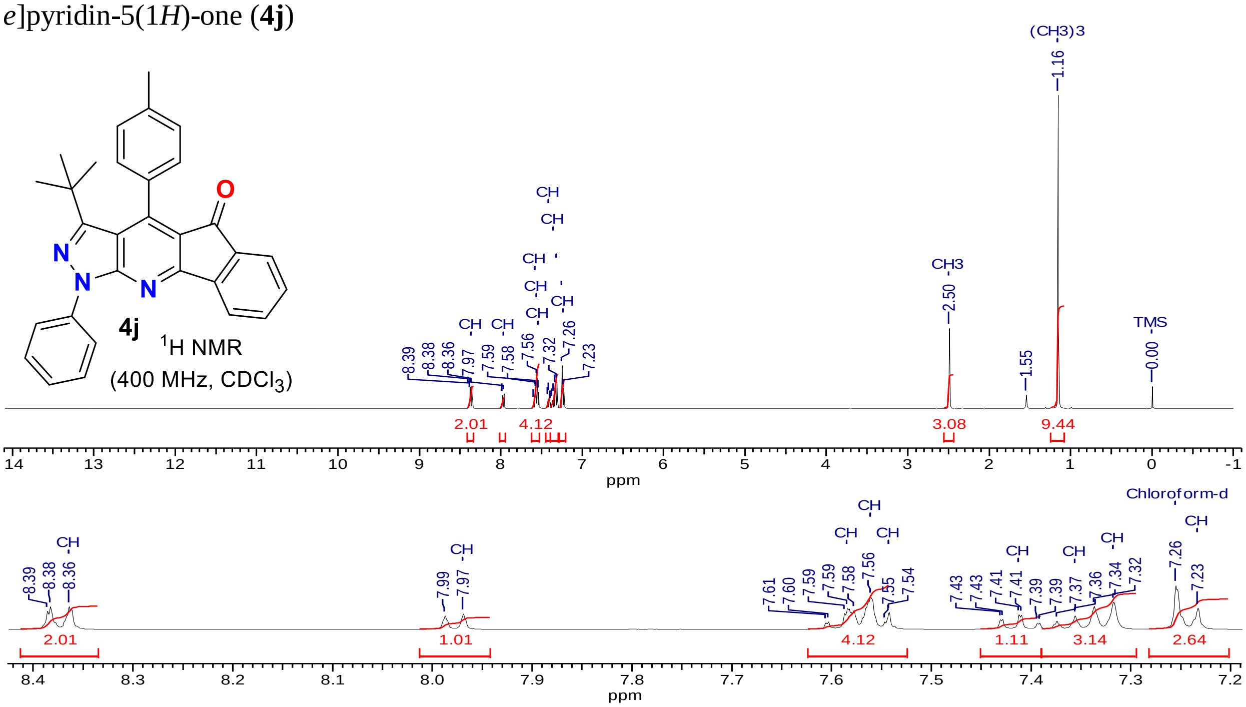

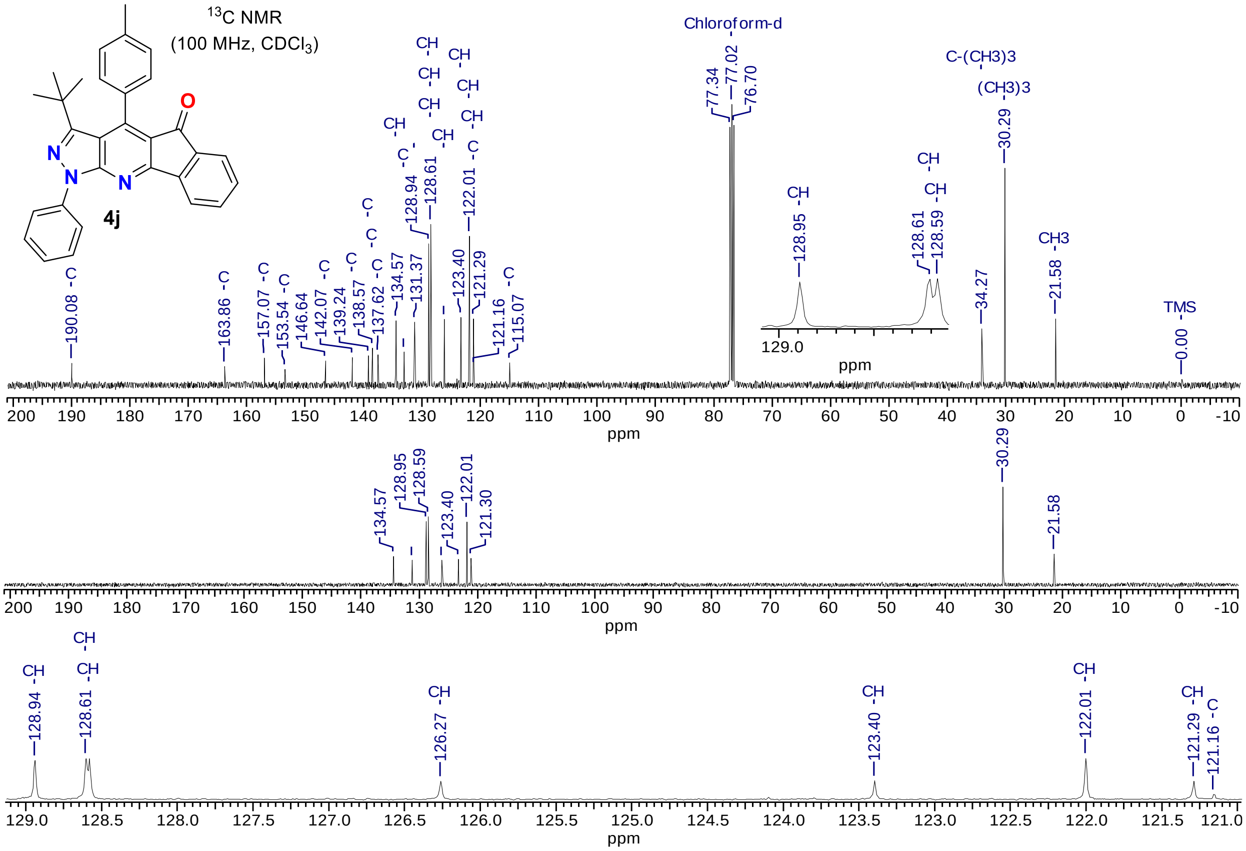

^{1}H and $^{13}C\{^{1}H\}$ NMR spectra of 3-(*tert*-butyl)-4-(4-chlorophenyl)-1-phenylindeno[1,2-*b*]pyrazolo[4,3-*e*]pyridin-5(1*H*)-one (**4k**)

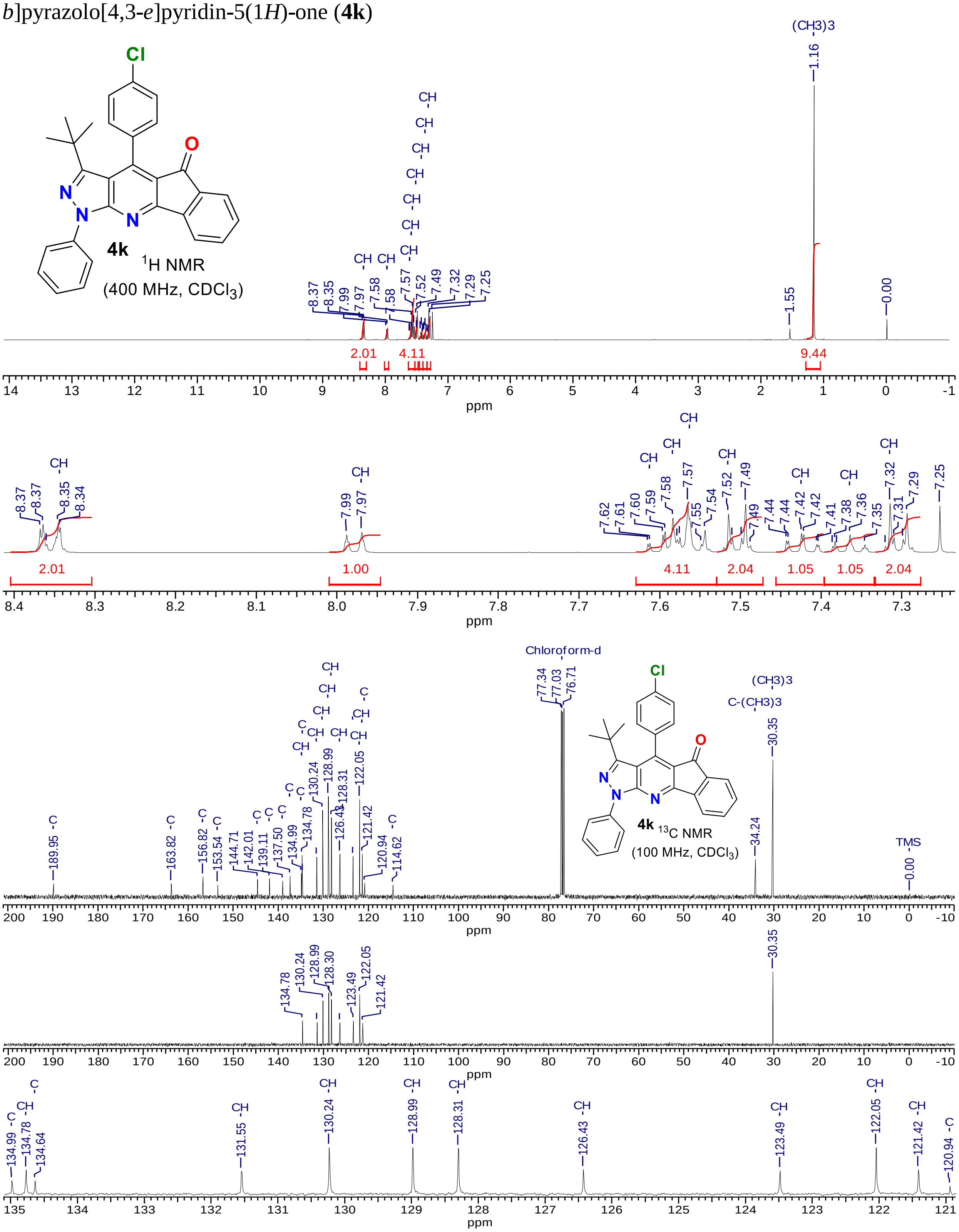

^{1}H and $^{13}C\{^{1}H\}$ NMR spectra of 4-(4-bromophenyl)-3-(*tert*-butyl)-1-phenylindeno[1,2-*b*]pyrazolo[4,3-*e*]pyridin-5(1*H*)-one (**4l**)

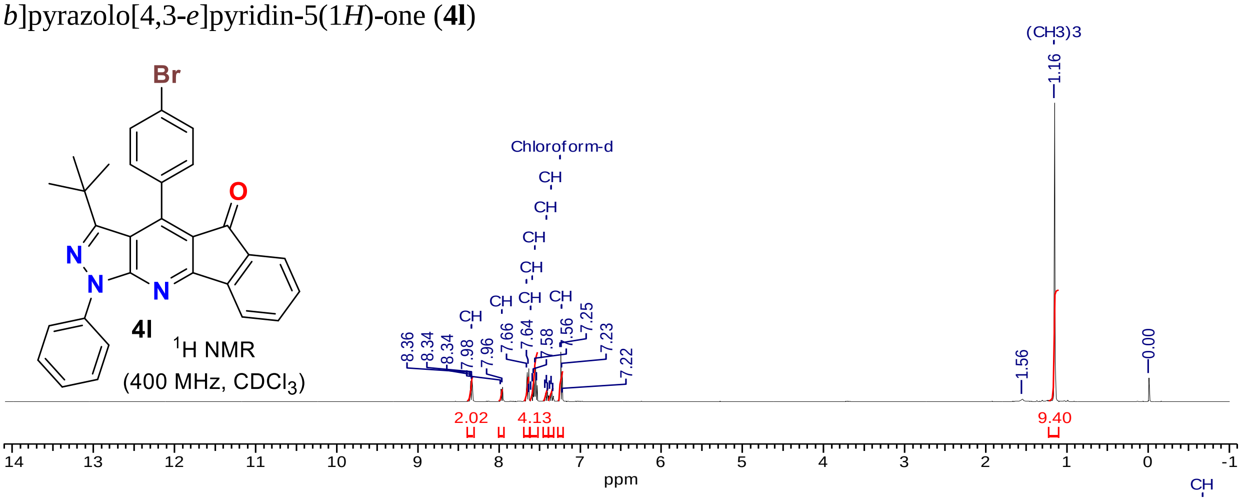

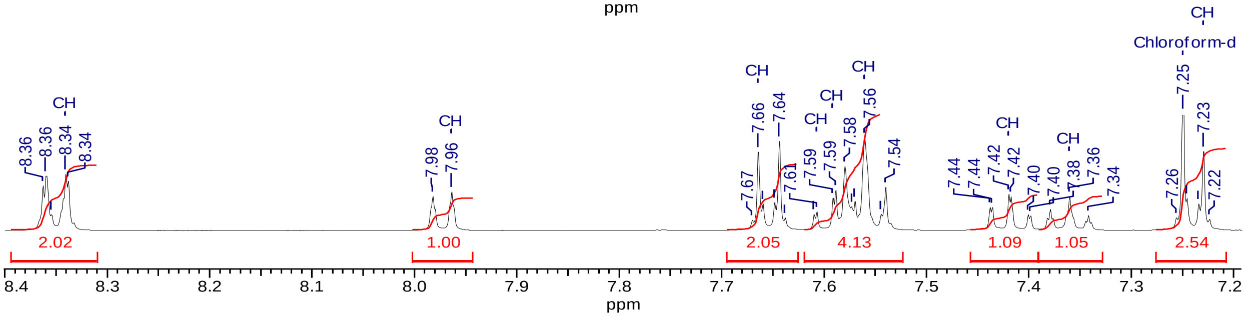

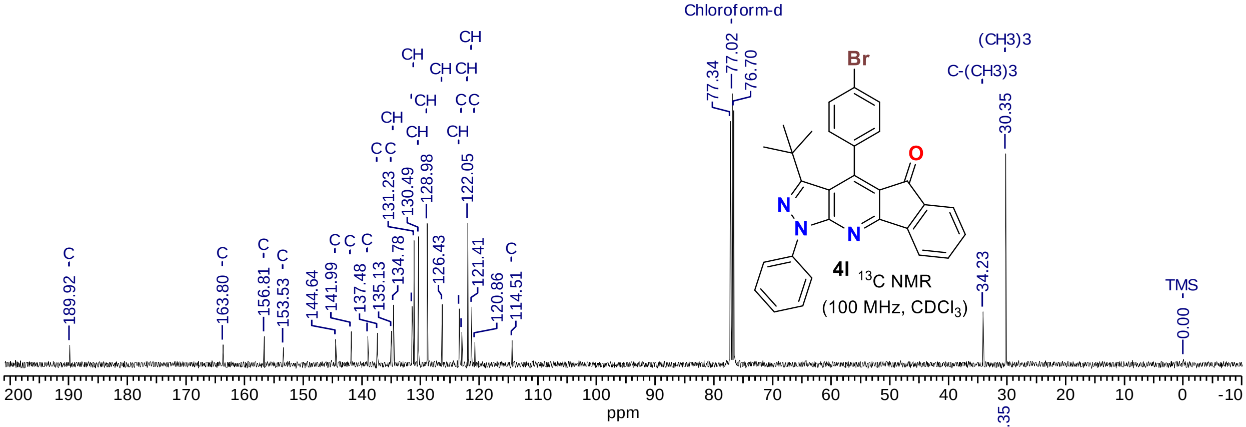

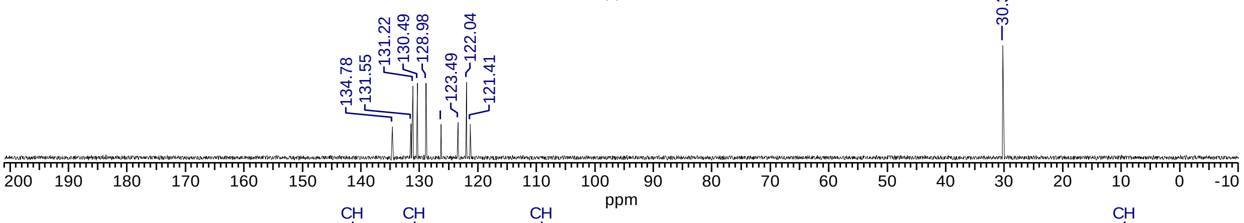

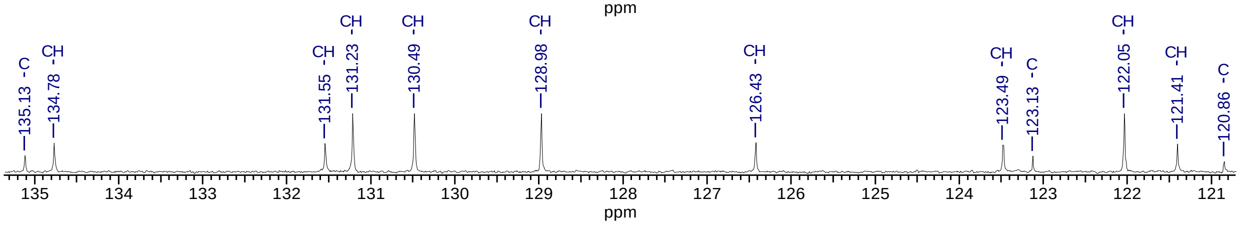

^{1}H and $^{13}C\{^{1}H\}$ NMR spectra of 3-(*tert*-butyl)-1-phenyl-4-(4-(trifluoromethyl)phenyl)indeno[1,2-*b*]pyrazolo[4,3-*e*]pyridin-5(1*H*)-one (**4m**)

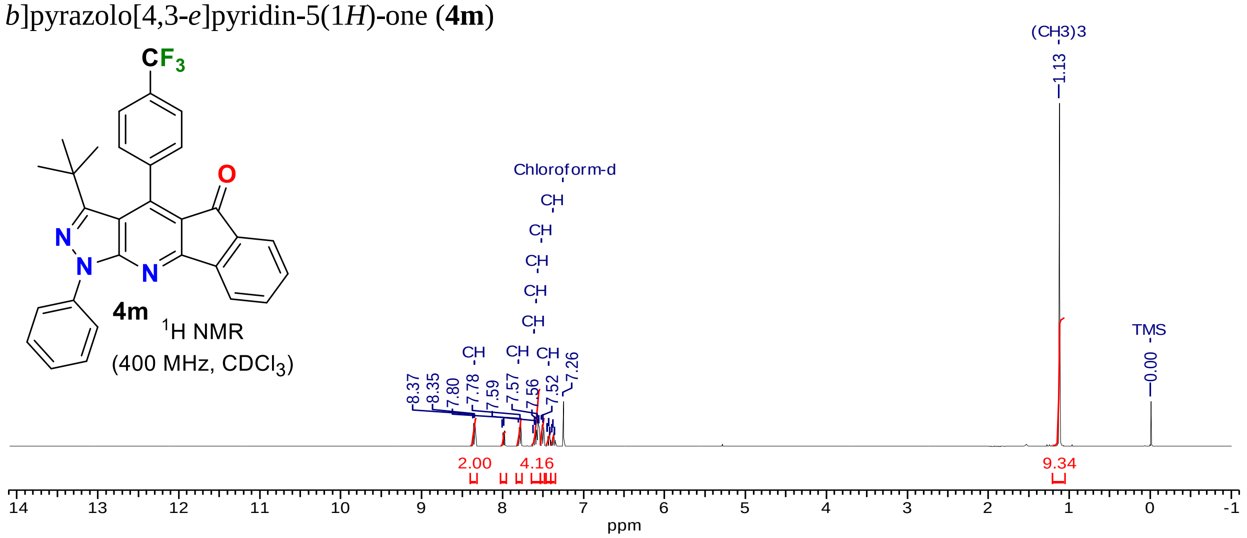

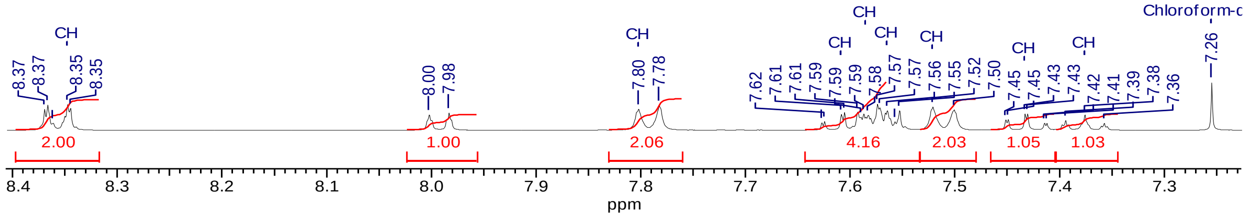

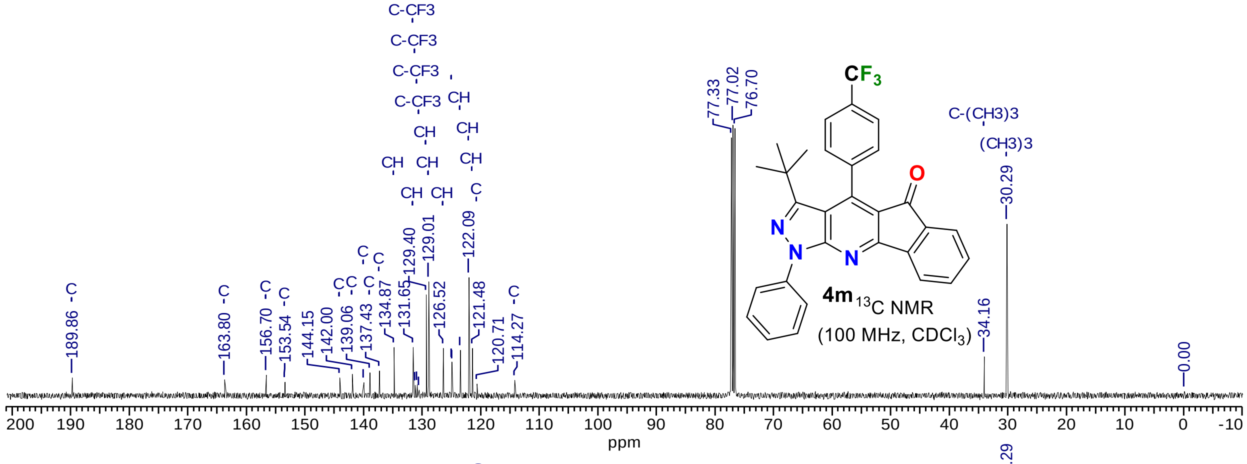

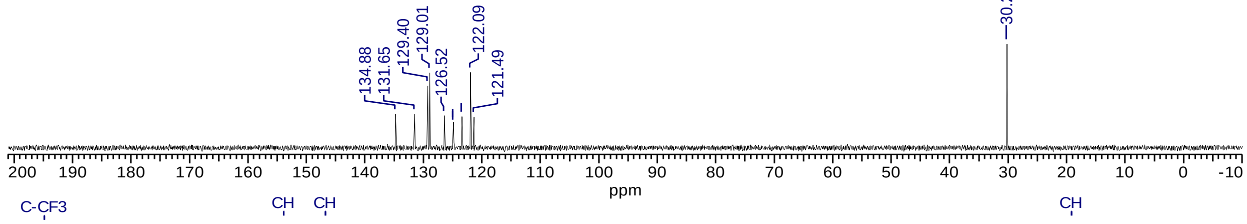

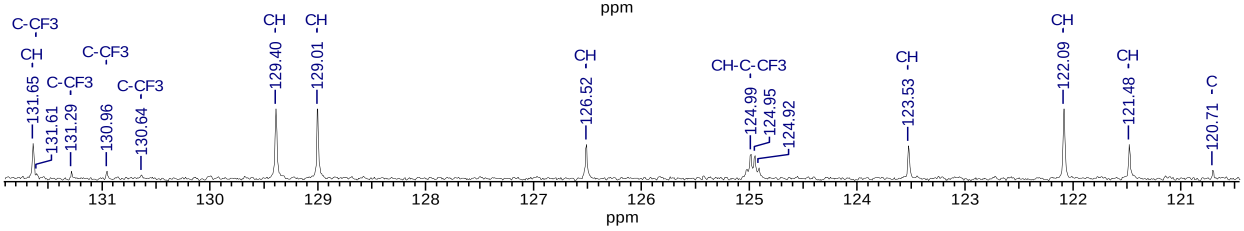

^{1}H and $^{13}C\{^{1}H\}$ NMR spectra of 3-(*tert*-butyl)-4-(4-methoxyphenyl)-1-phenylindeno[1,2-*b*]pyrazolo[4,3-*e*]pyridin-5(1*H*)-one (**4n**)

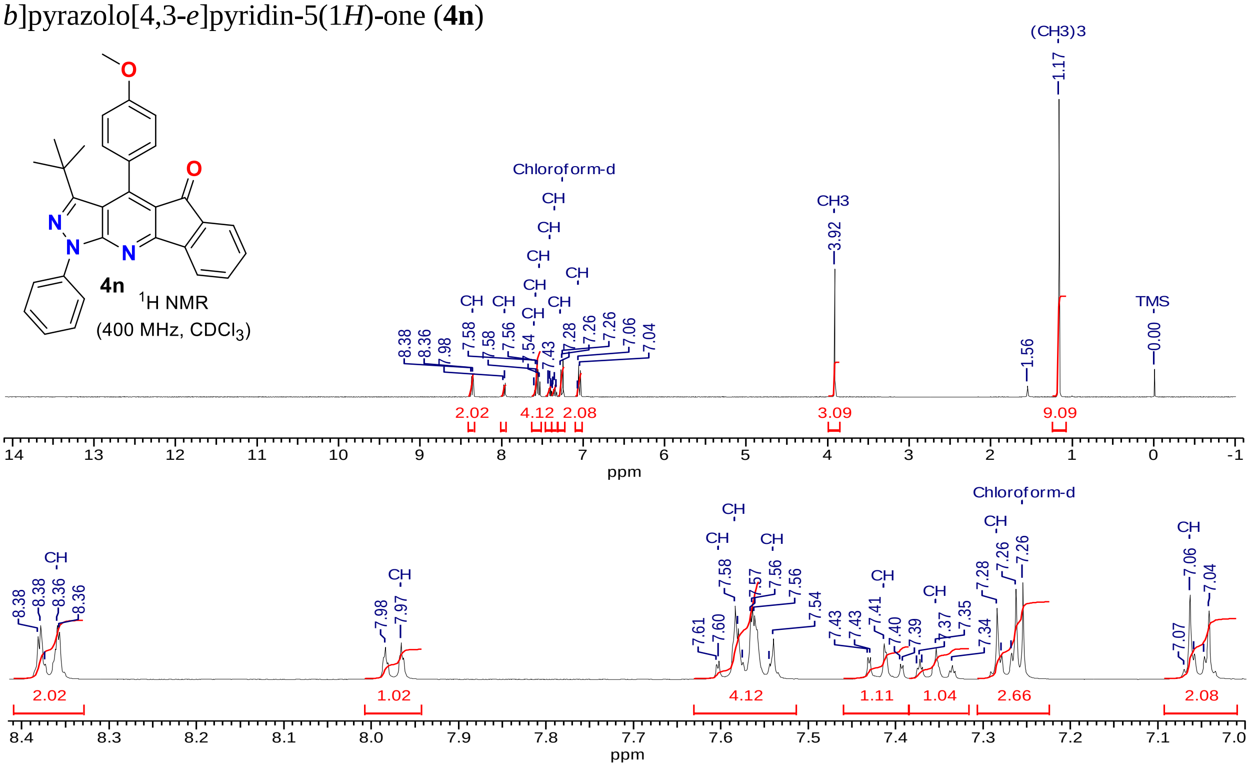

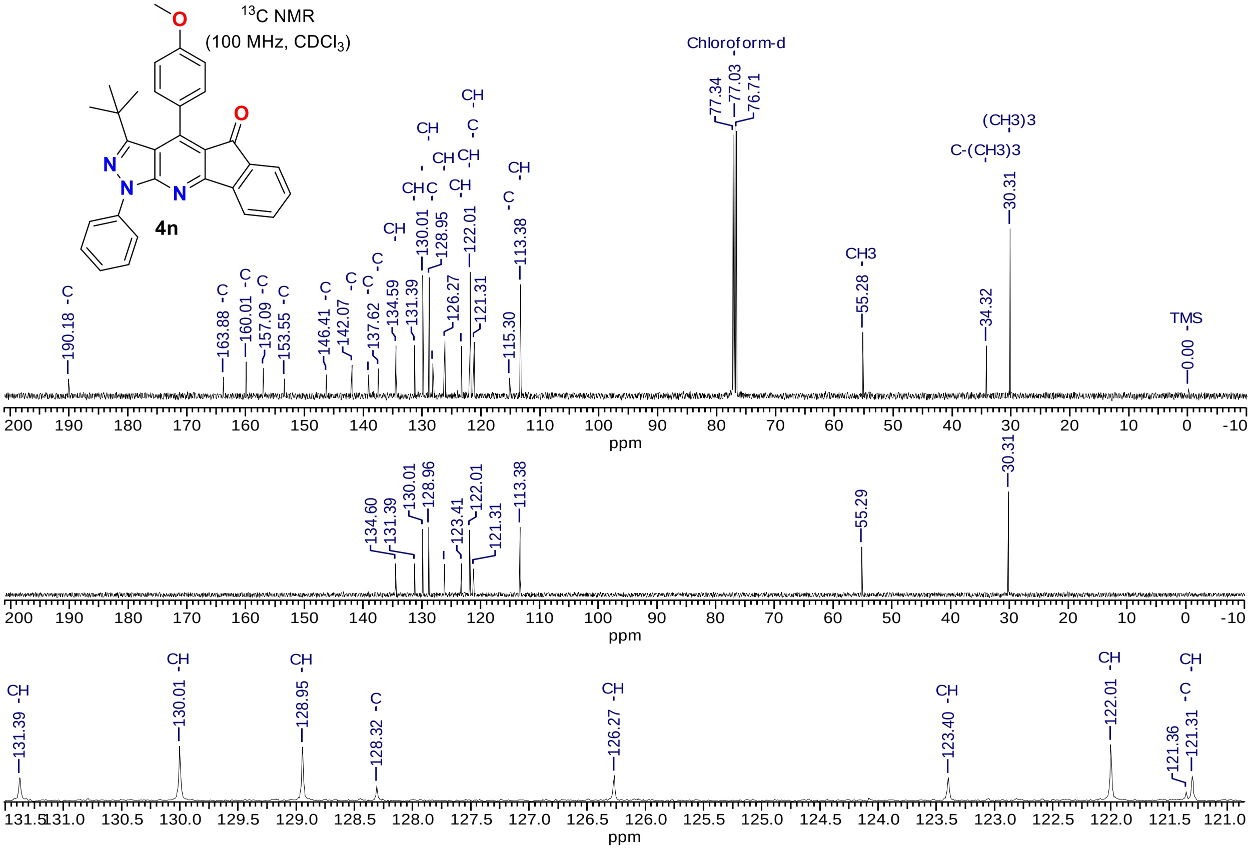

^{1}H and $^{13}C\{^{1}H\}$ NMR spectra of 3-(*tert*-butyl)-4-(4-nitrophenyl)-1-phenylindeno[1,2-*b*]pyrazolo[4,3-*e*]pyridin-5(1*H*)-one (**4o**)

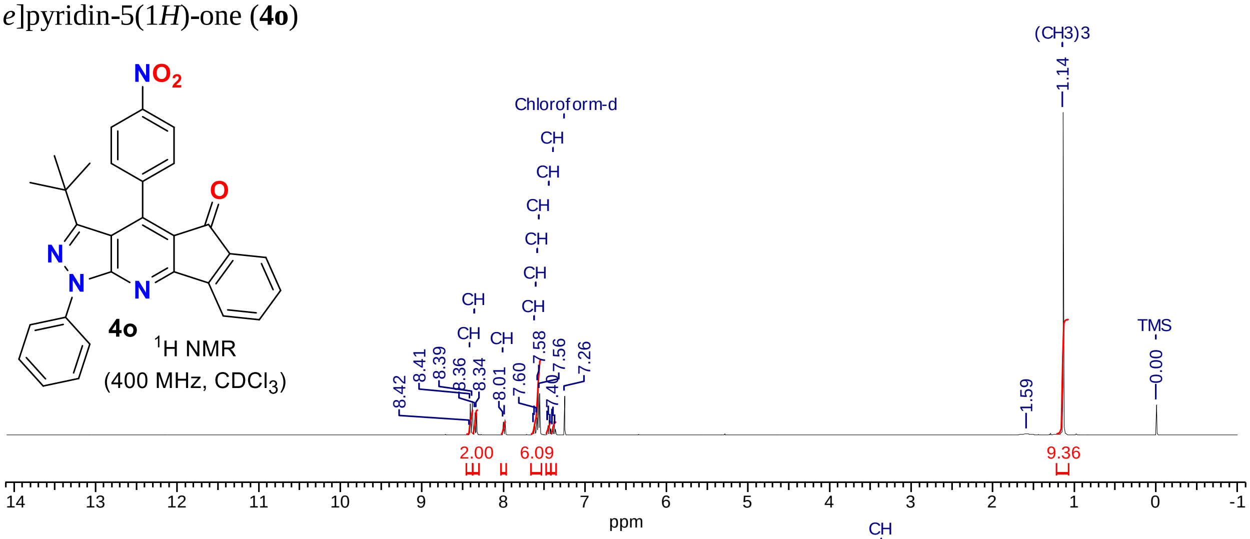

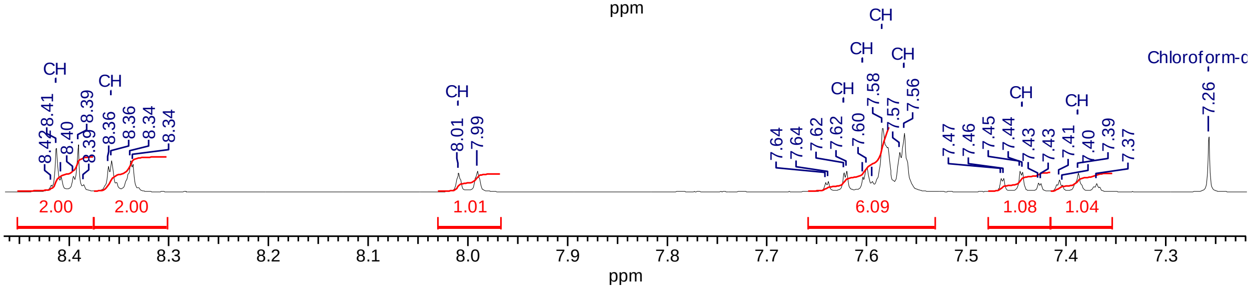

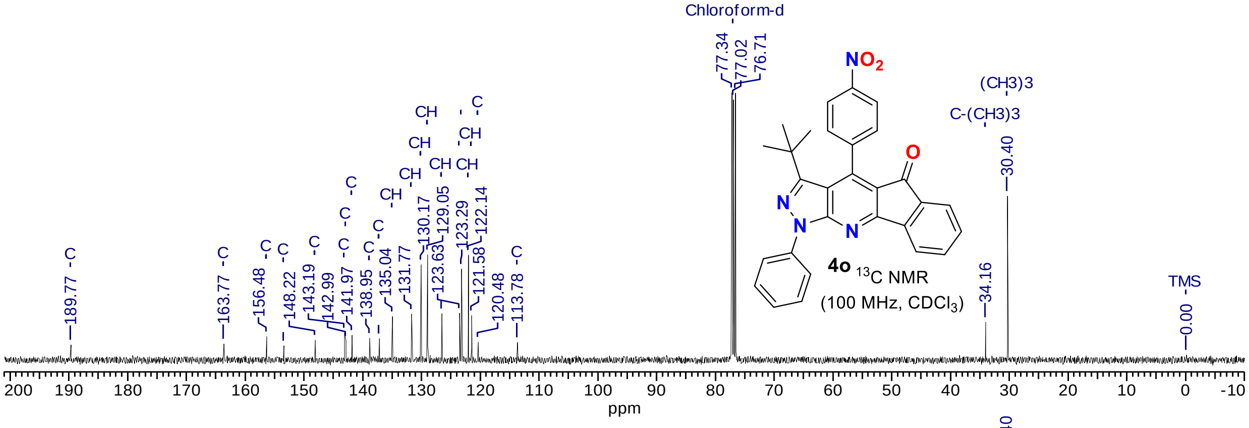

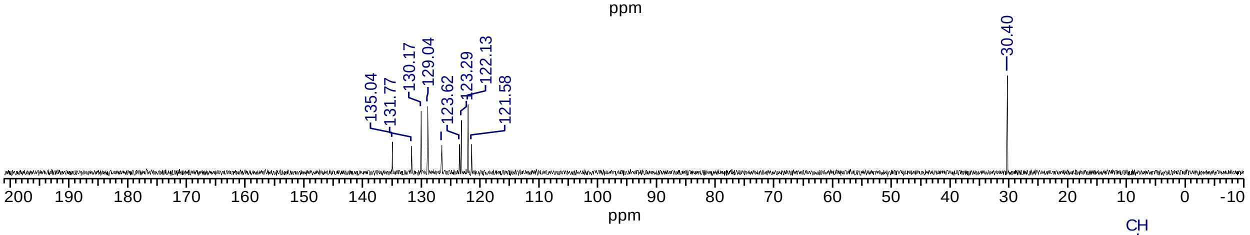

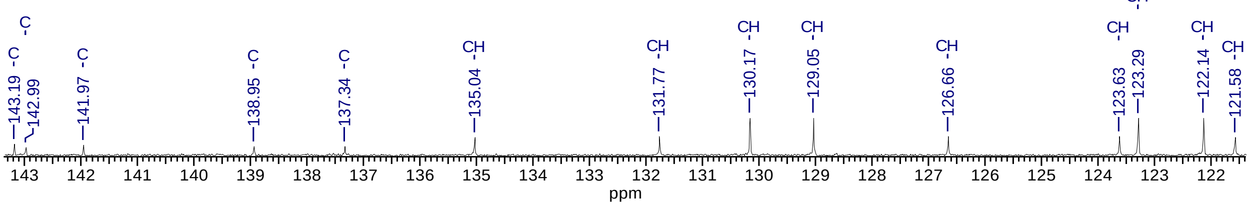

^{1}H and ^{13}C{^{1}H} NMR spectra of 3-(*tert*-Butyl)-1-(4-chlorophenyl)-4-phenylindeno[1,2-*b*]pyrazolo[4,3-*e*]pyridin-5(1*H*)-one (**4p**)

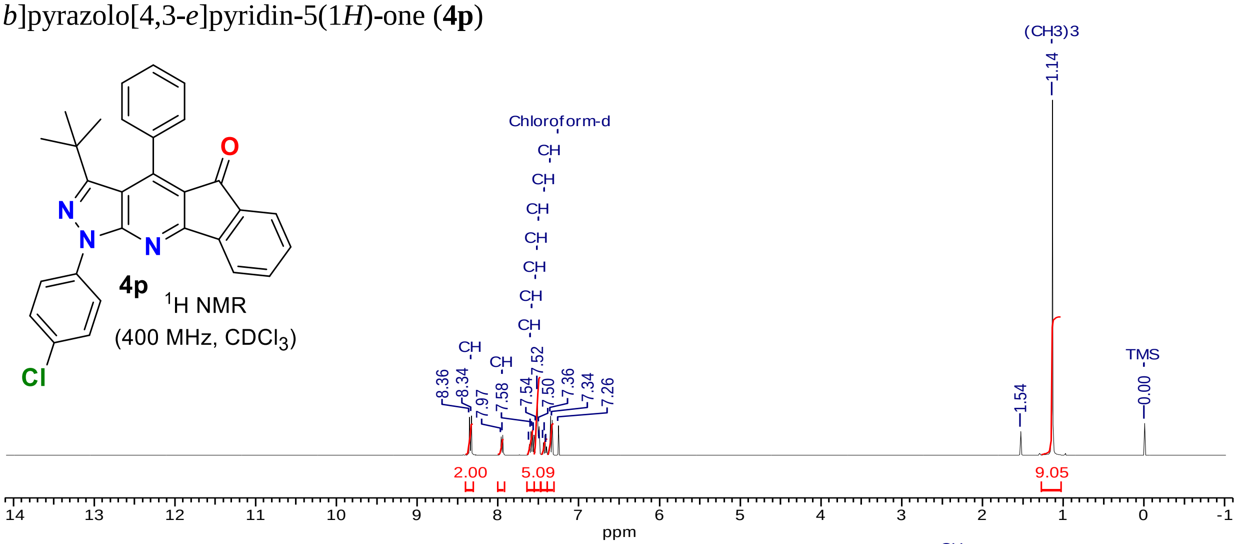

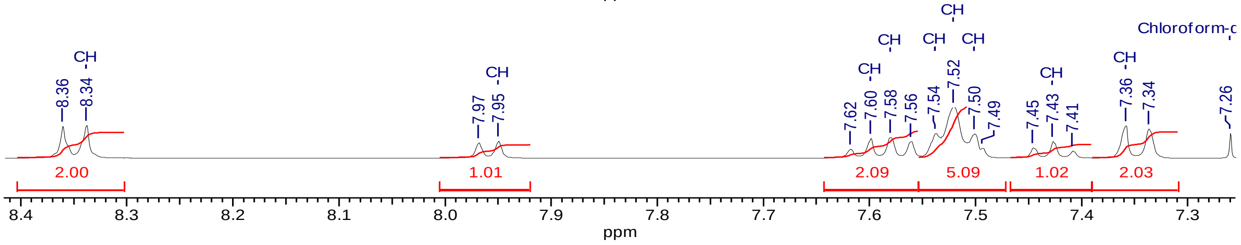

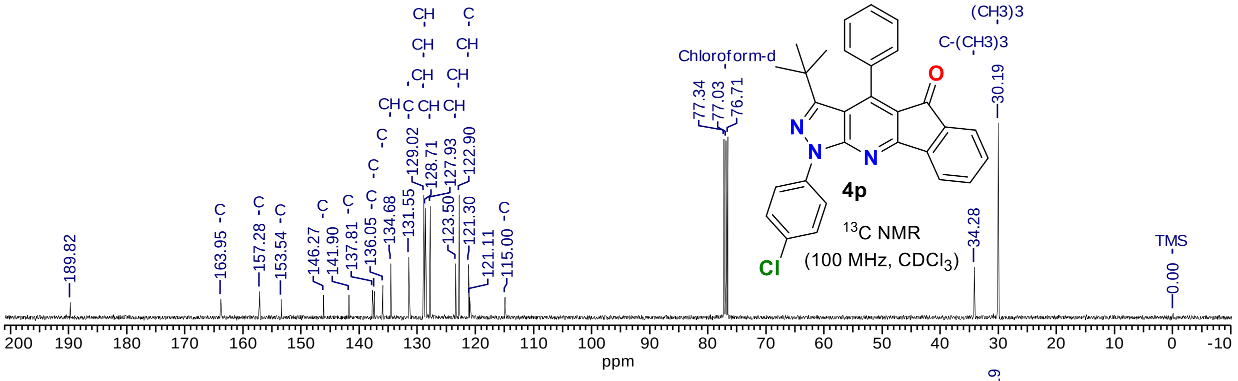

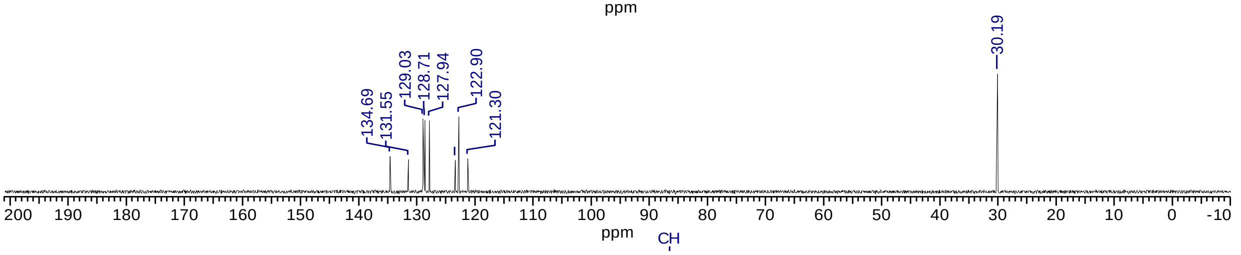

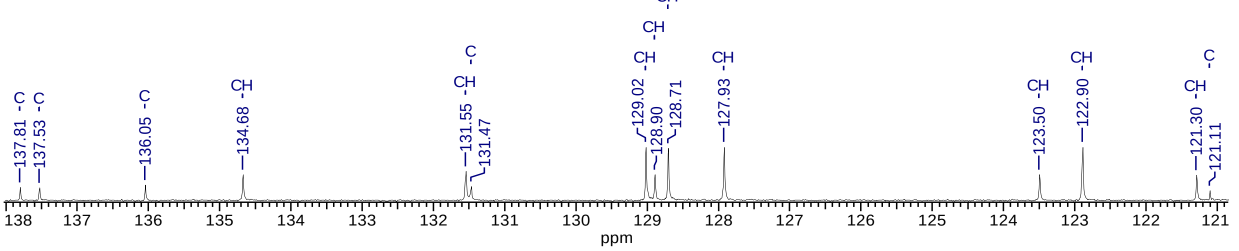

^{1}H and $^{13}C\{^{1}H\}$ NMR spectra of 3-(*tert*-butyl)-1-(4-chlorophenyl)-4-(*p*-tolyl)indeno[1,2-*b*]pyrazolo[4,3-*e*]pyridin-5(1*H*)-one (**4q**)

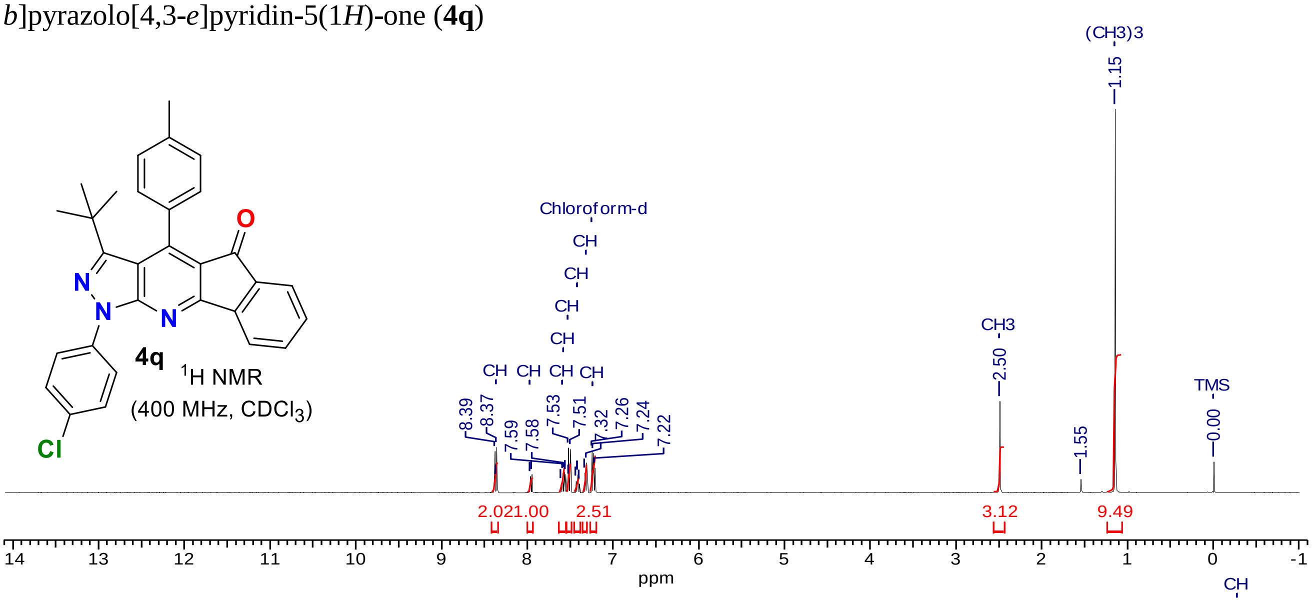

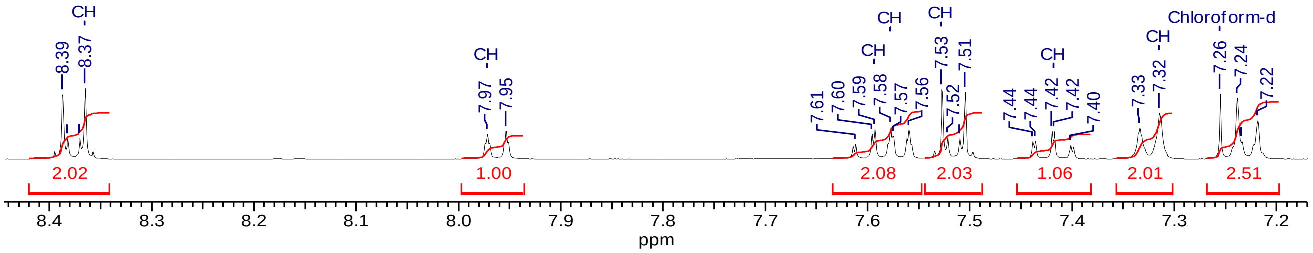

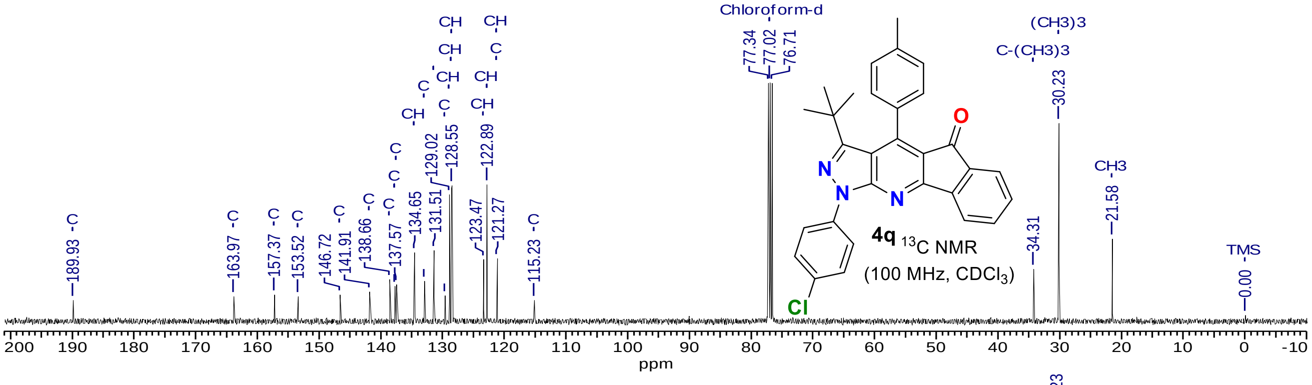

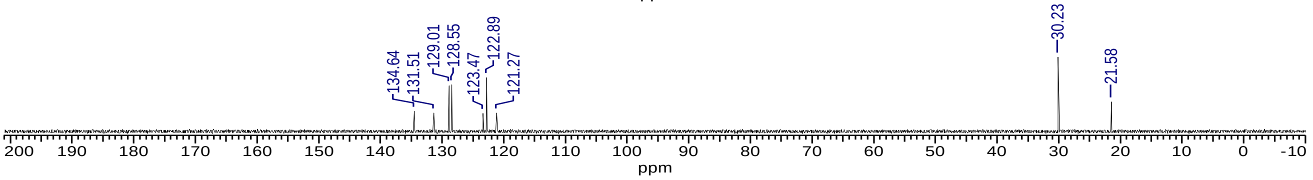

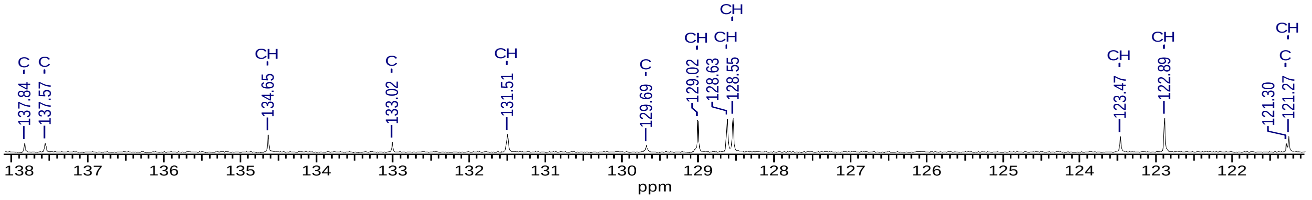

^1H and $^{13}C\{^1H\}$ NMR spectra of 3-(*tert*-butyl)-1,4-*bis*(4-chlorophenyl)indeno[1,2-*b*]pyrazolo[4,3-*e*]pyridin-5(1*H*)-one (**4r**)

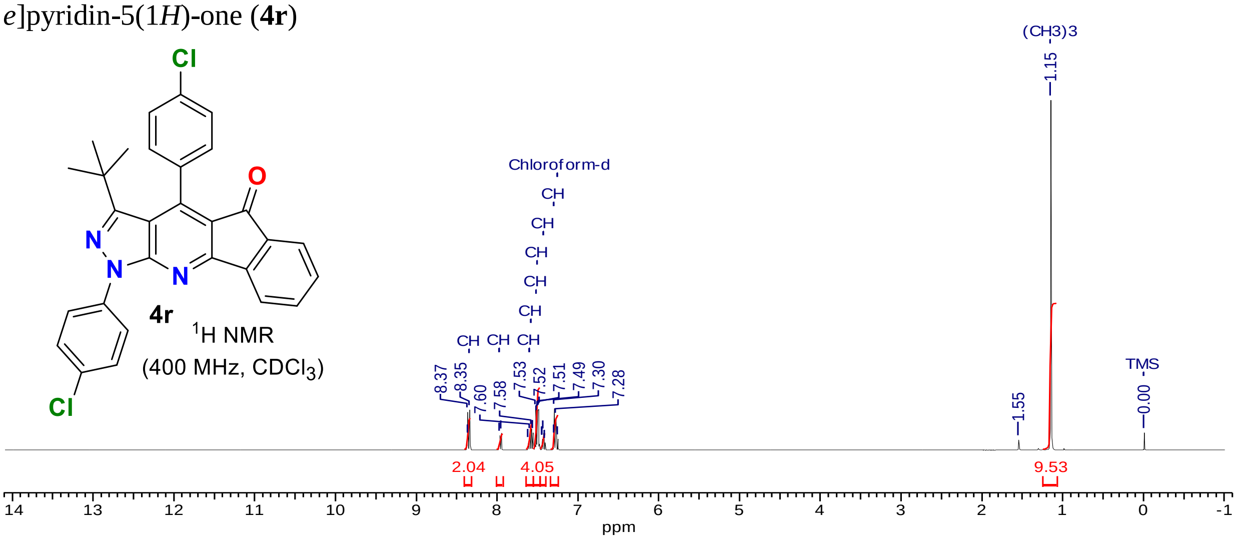

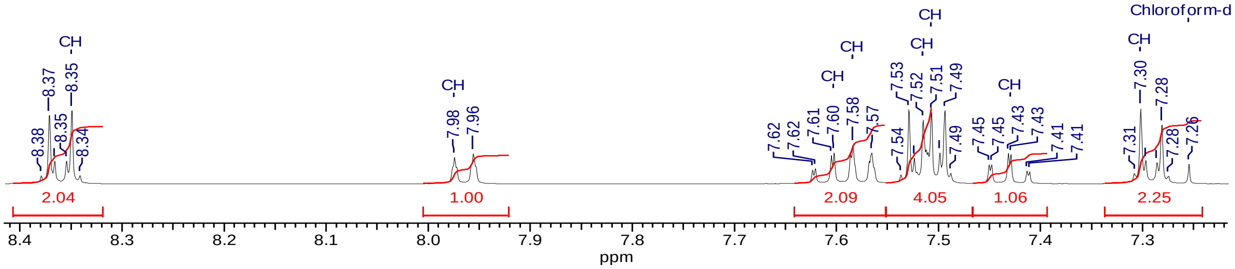

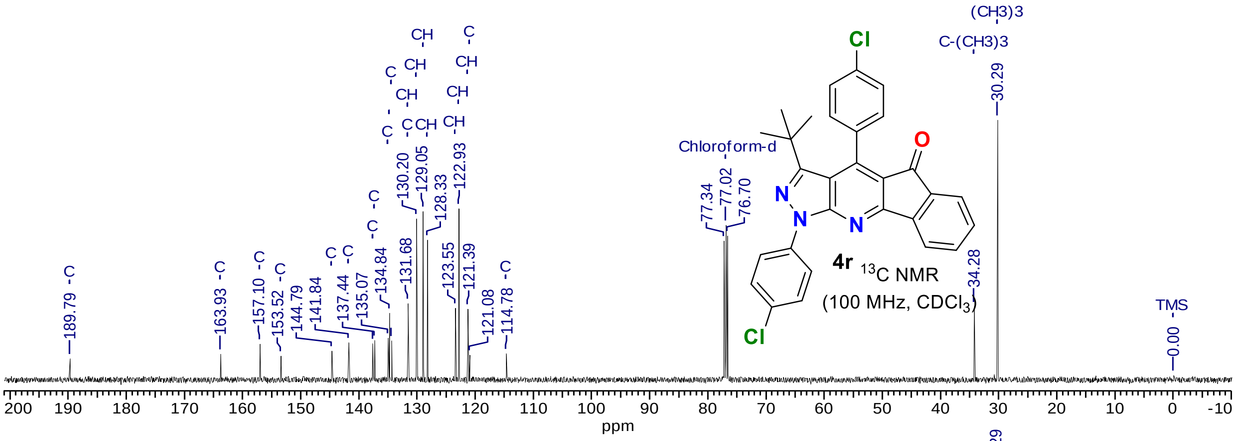

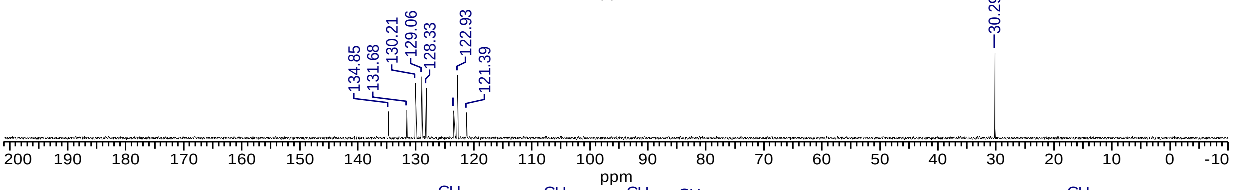

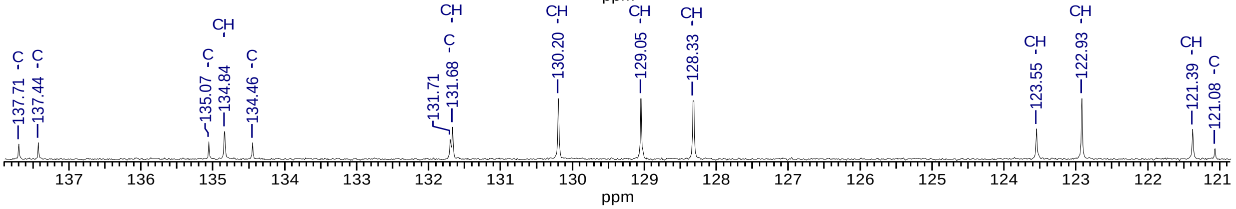

^{1}H and ^{13}C{^{1}H} NMR spectra of 4-(4-bromophenyl)-3-(*tert*-butyl)-1-(4-chlorophenyl)indeno[1,2-*b*]pyrazolo[4,3-*e*]pyridin-5(1*H*)-one (**4s**)

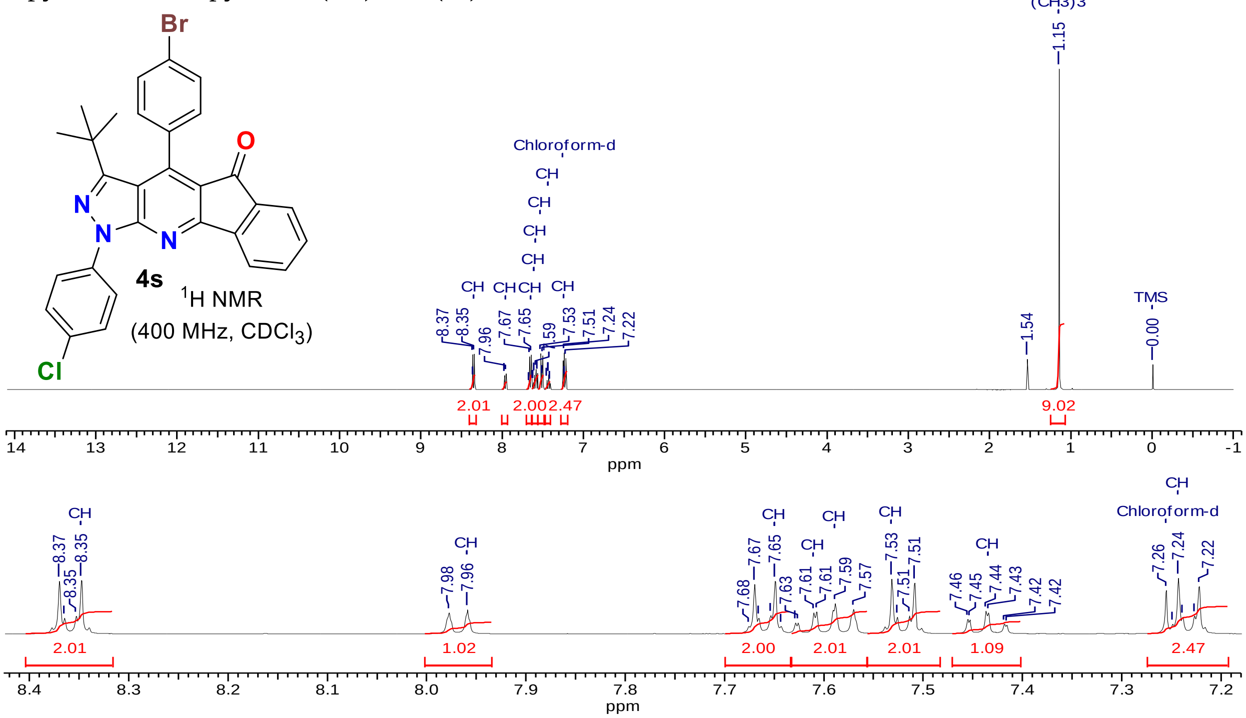

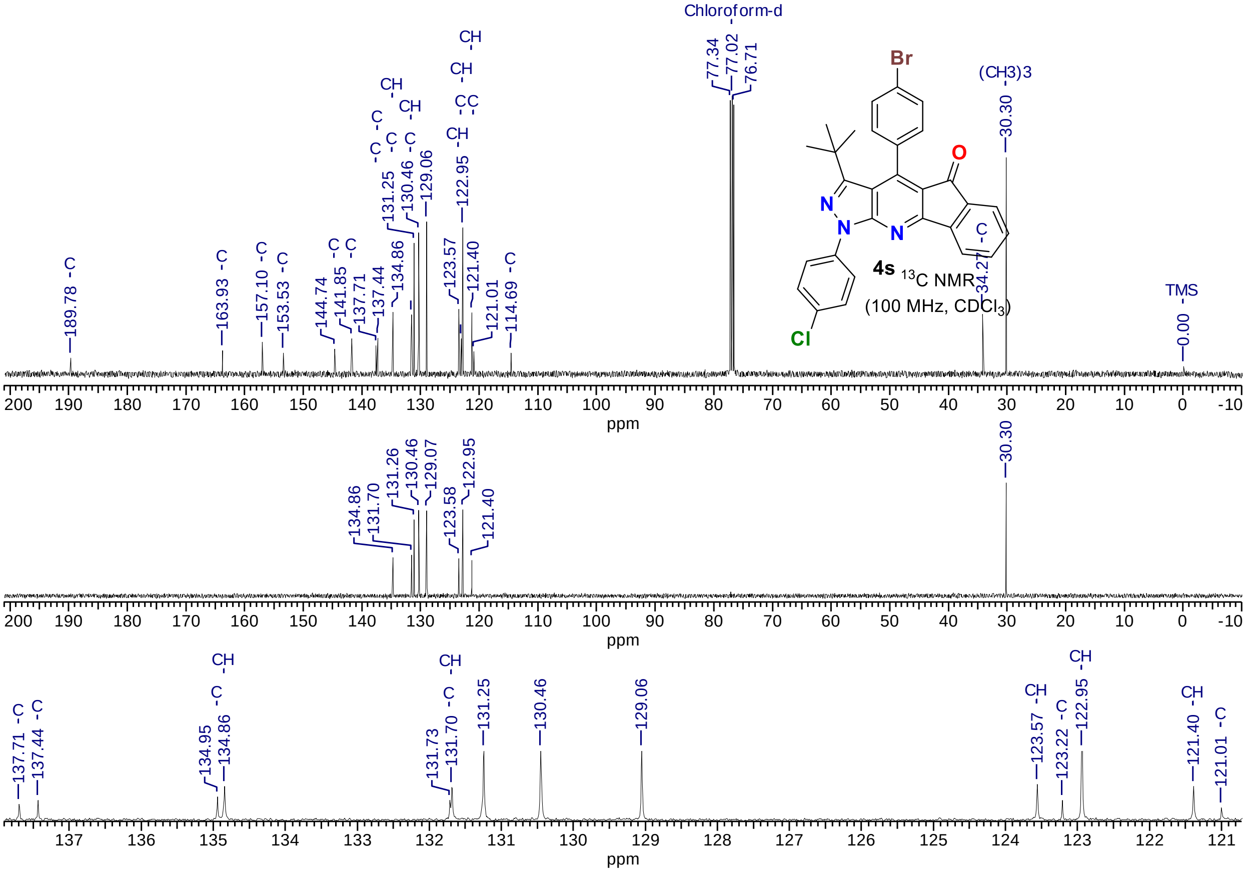

^{1}H and ^{13}C{^{1}H} NMR spectra of 3-(*tert*-butyl)-1-(4-chlorophenyl)-4-(4-(trifluoromethyl)phenyl)-indeno[1,2-*b*]pyrazolo[4,3-*e*]pyridin-5(1*H*)-one (**4t**)

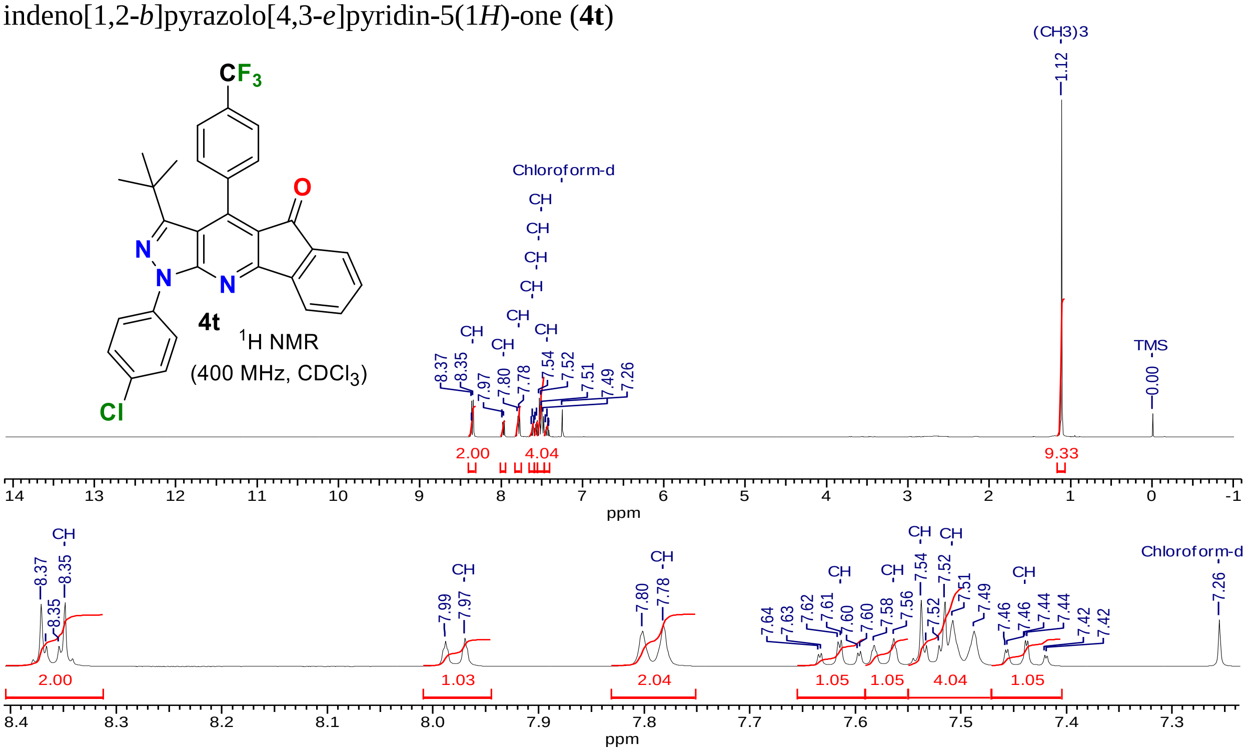

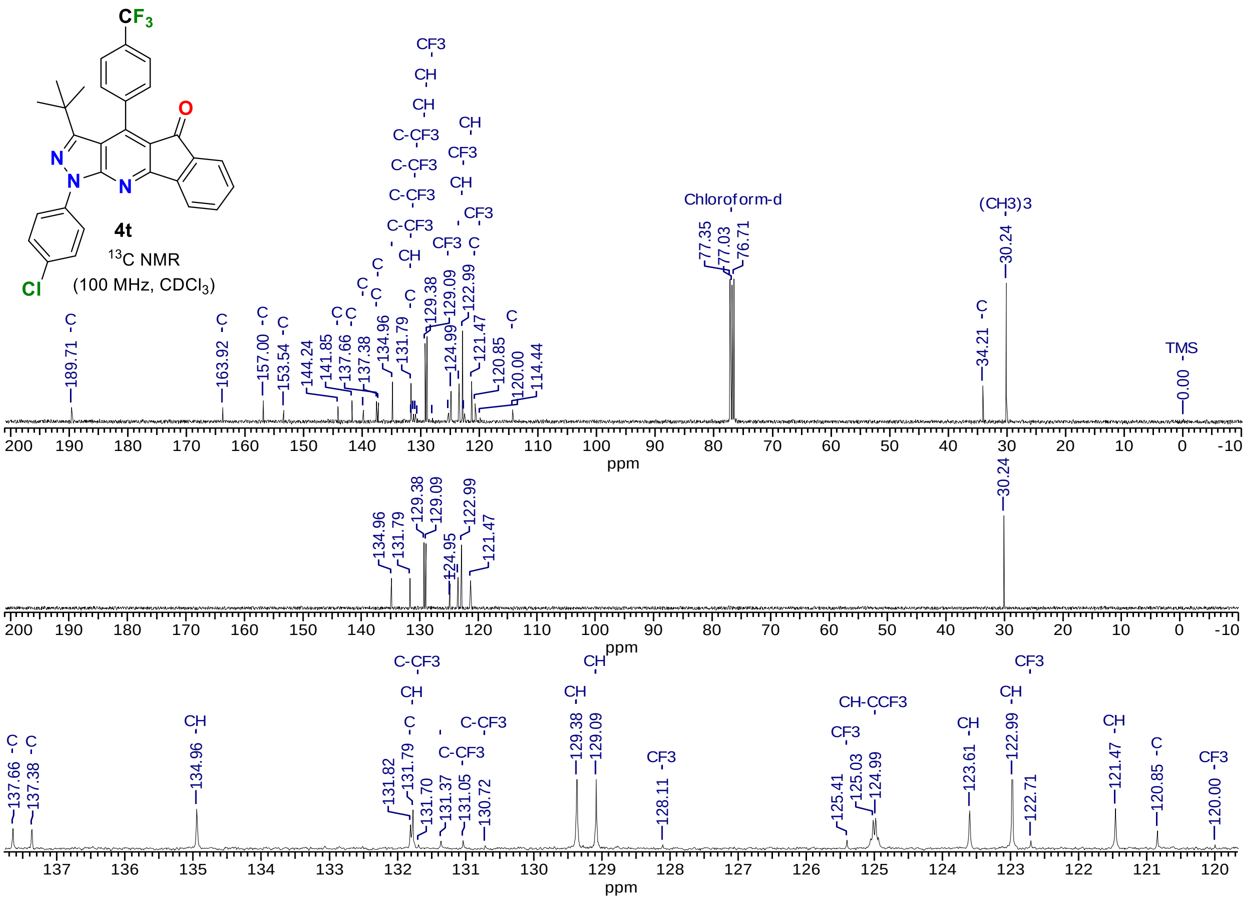

^{1}H and ^{13}C{^{1}H} NMR spectra of 3-(*tert*-butyl)-1-(4-chlorophenyl)-4-(4-methoxyphenyl)indeno[1,2-*b*]pyrazolo[4,3-*e*]pyridin-5(1*H*)-one (**4u**)

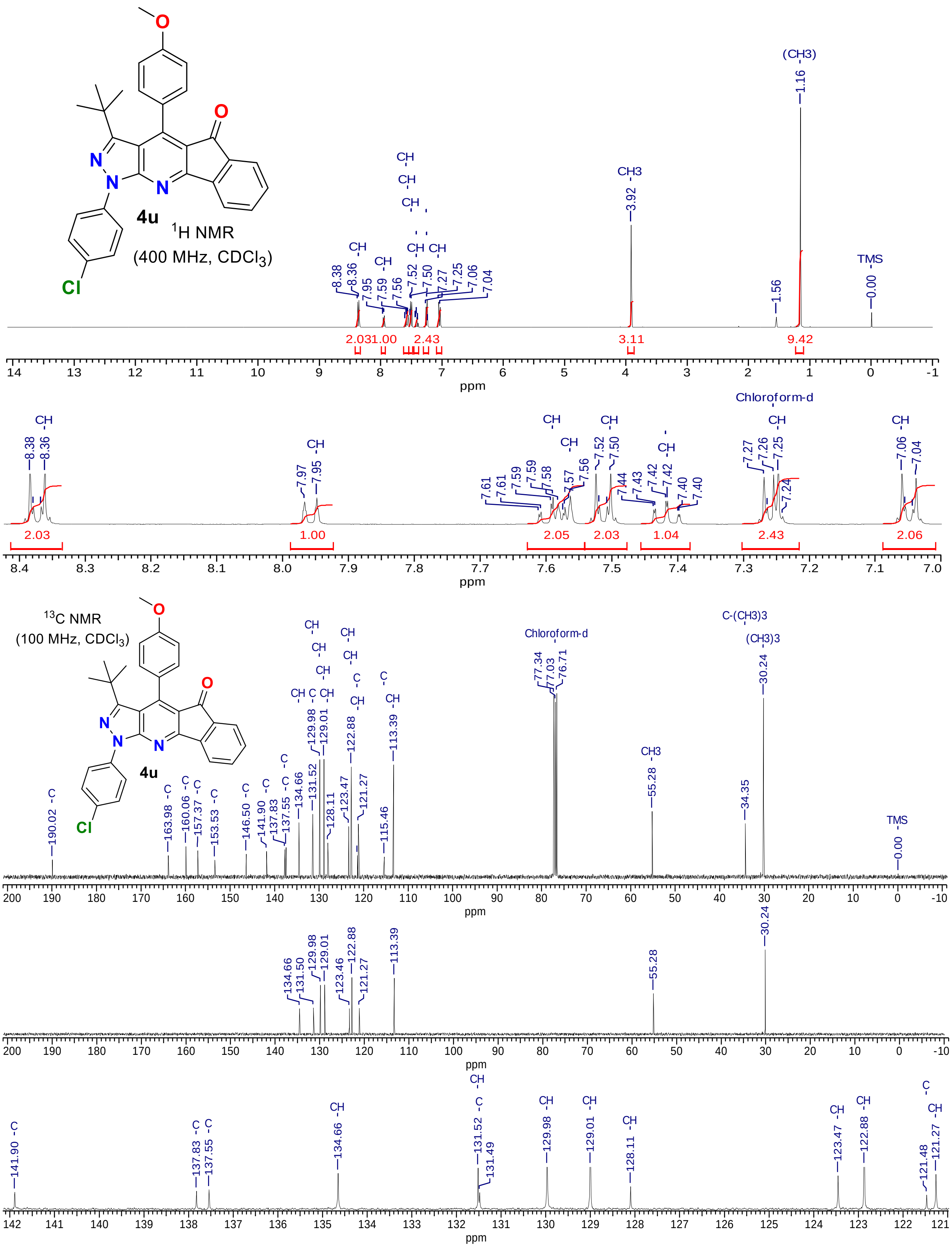

^{1}H and ^{13}C{^{1}H} NMR spectra of 3-(*tert*-butyl)-1-(4-chlorophenyl)-4-(4-nitrophenyl)indeno[1,2-*b*]pyrazolo[4,3-*e*]pyridin-5(1*H*)-one (**4v**)

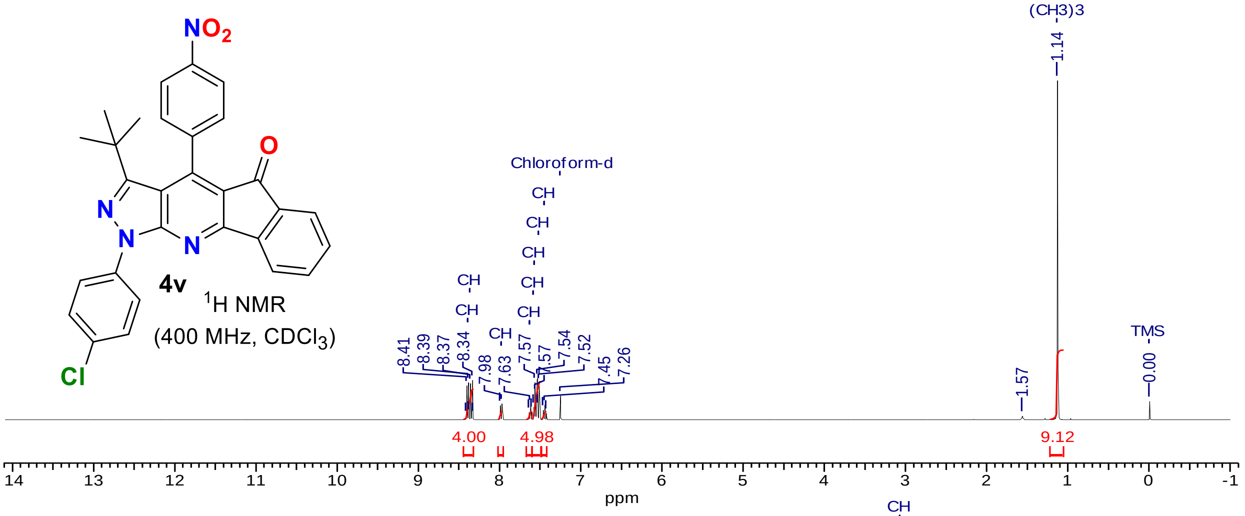

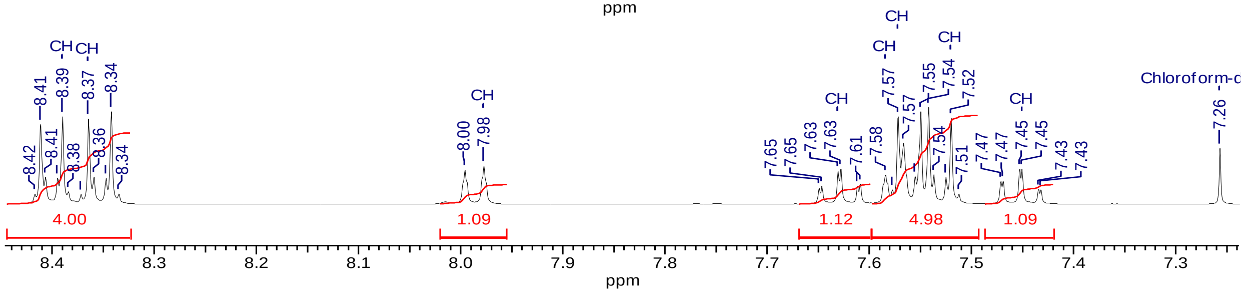

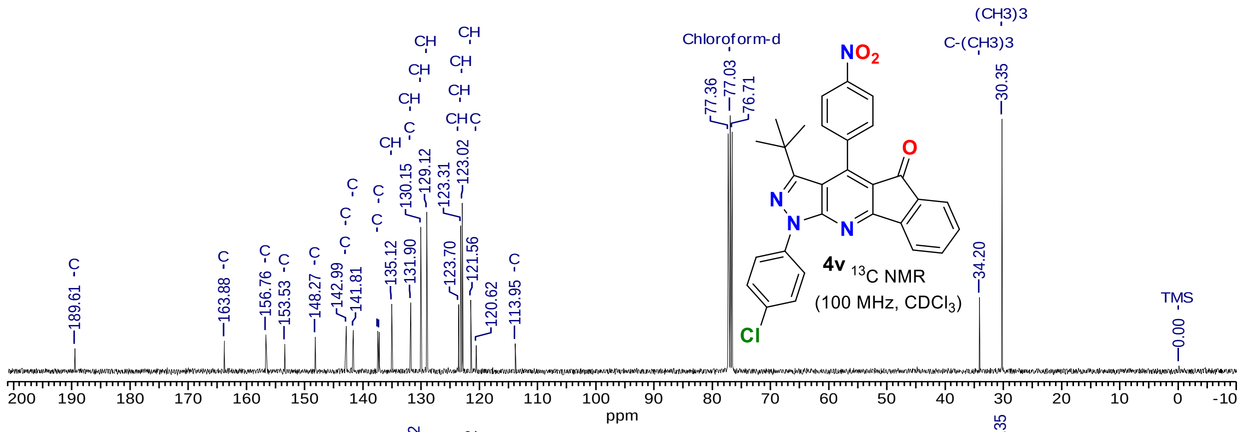

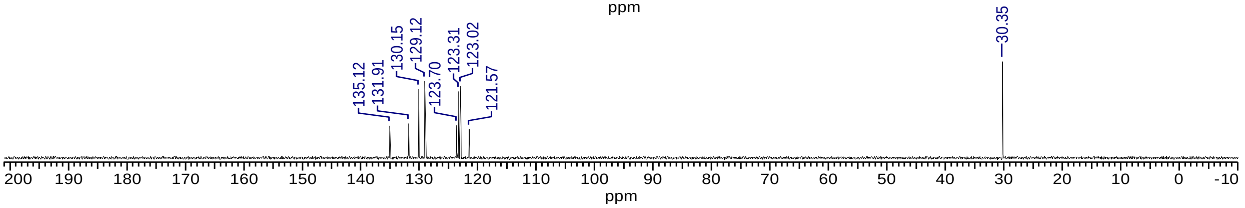

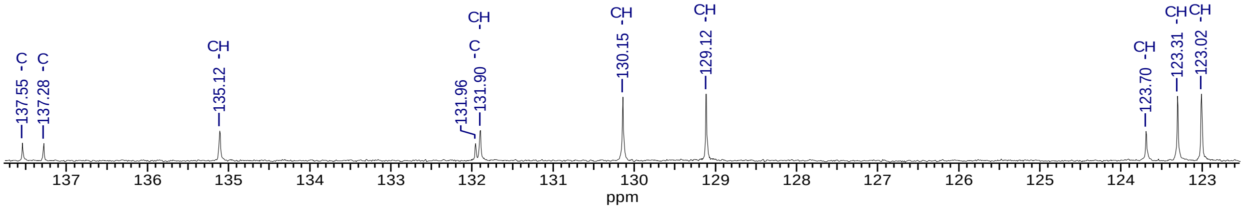

^{1}H and $^{13}C\{^{1}H\}$ NMR spectra of 1-(4-chlorophenyl)-3-methyl-4-phenylindeno[1,2-*b*]pyrazolo[4,3-*e*]pyridin-5(1*H*)-one (**4w**)

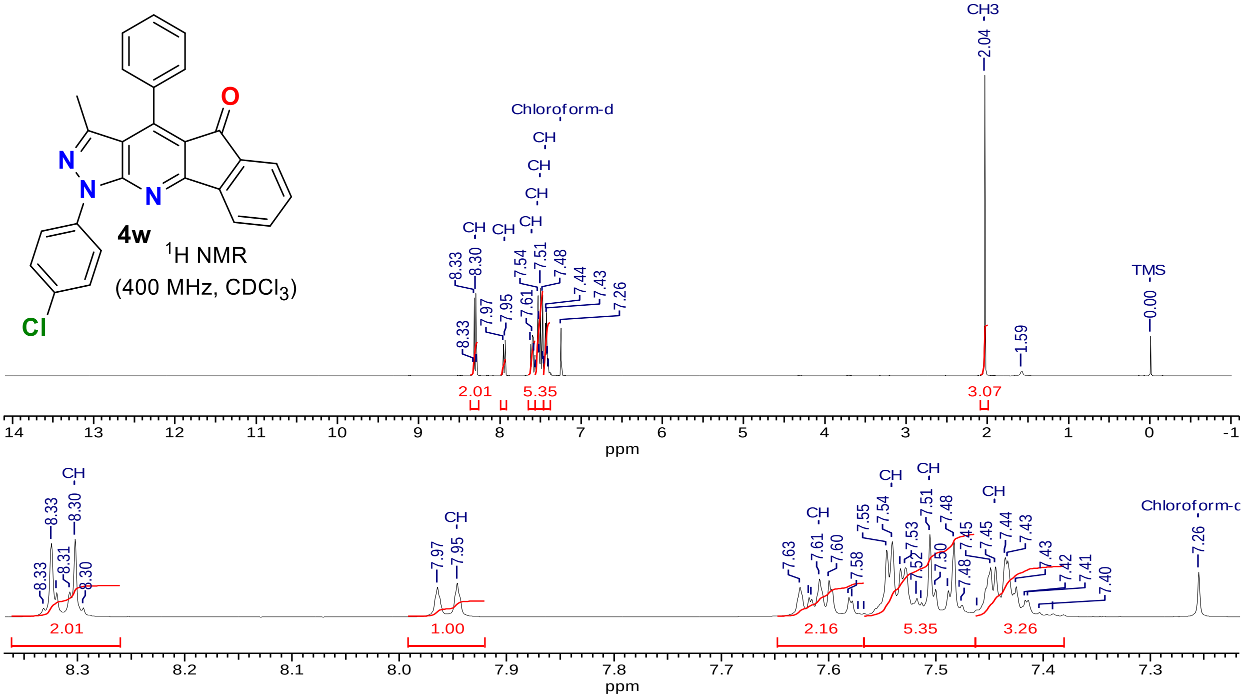

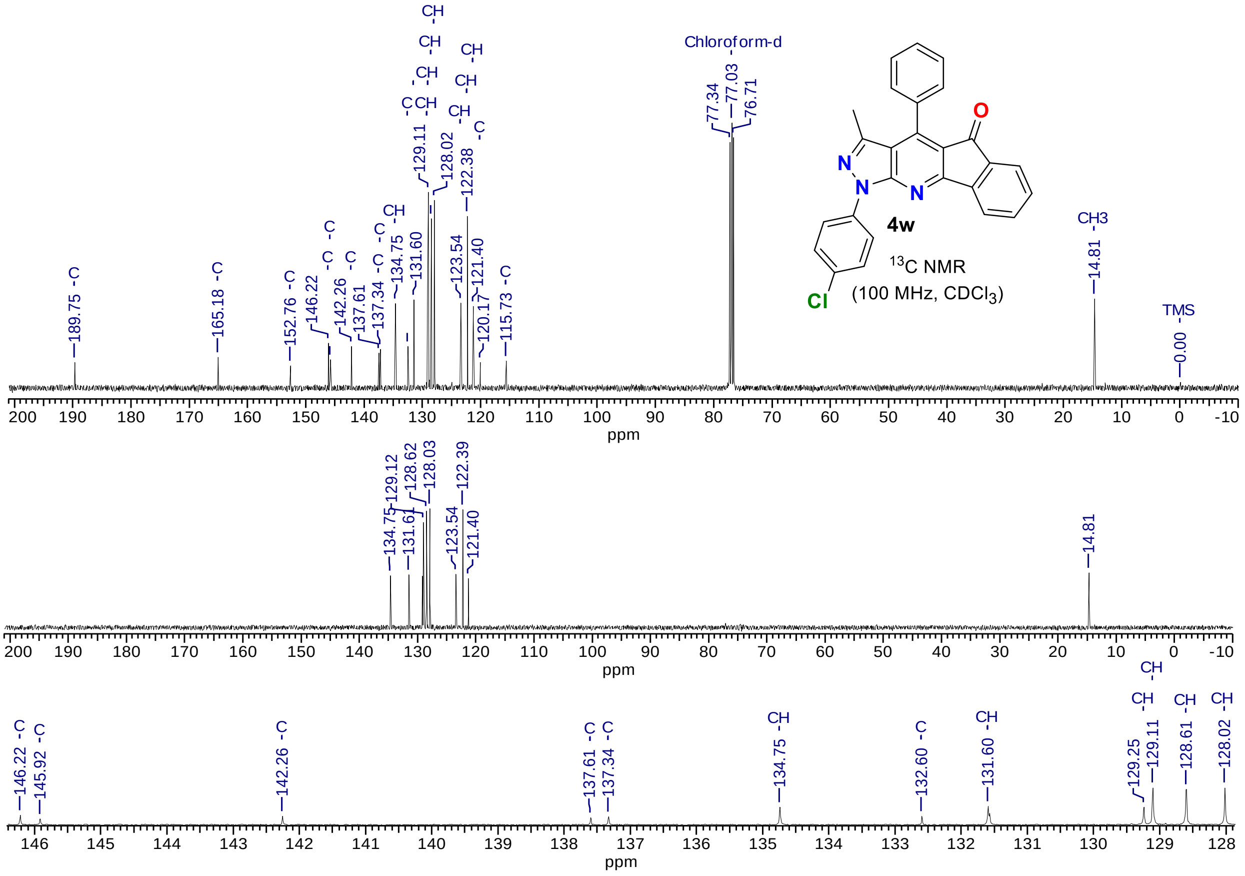

^{1}H and ^{13}C{^{1}H} NMR spectra of 3-(*tert*-Butyl)-1-(4-nitrophenyl)-4-phenylindeno[1,2-*b*]pyrazolo[4,3-*e*]pyridin-5(1*H*)-one (**4x**)

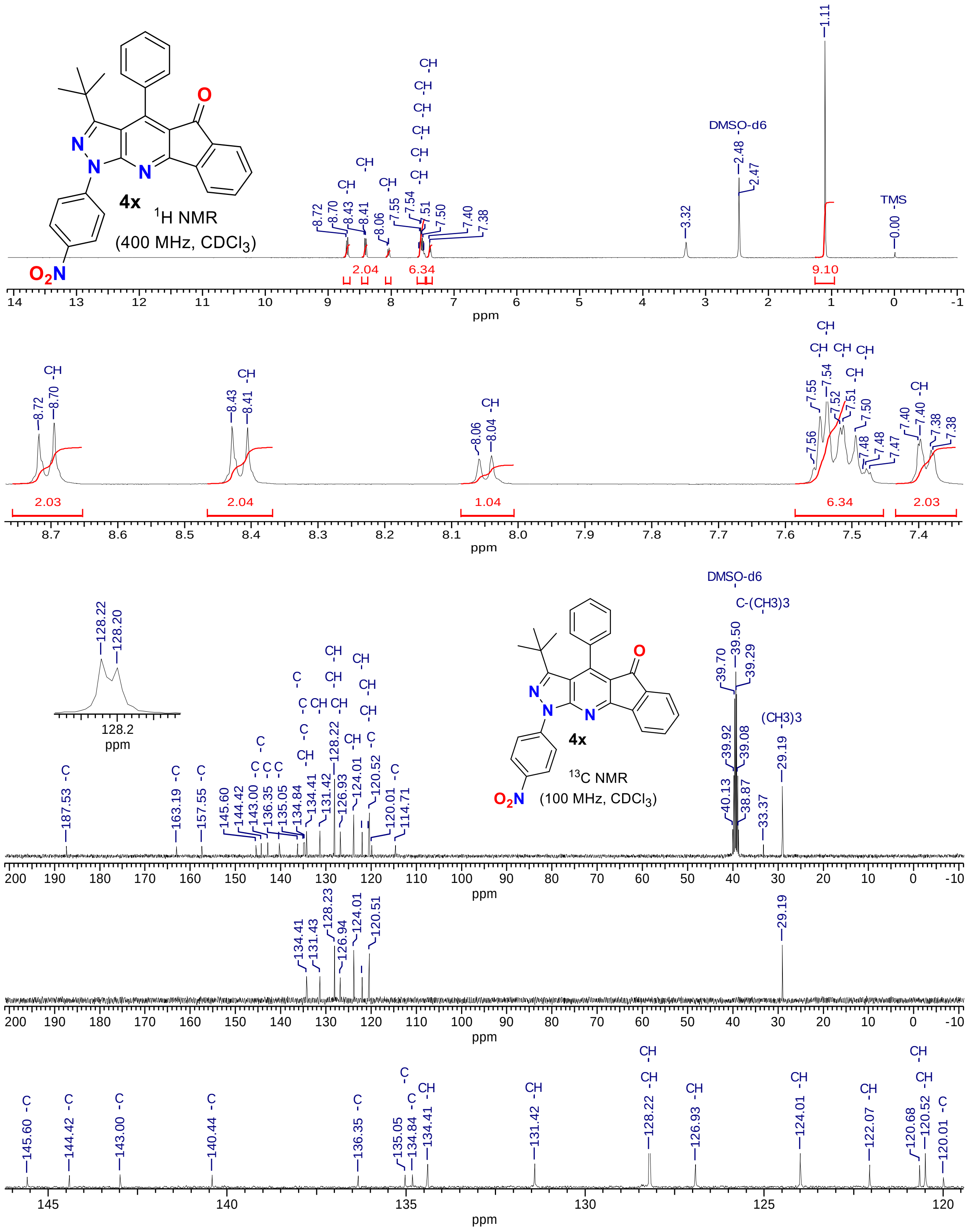

^{1}H and $^{13}C\{^{1}H\}$ NMR spectra of 2-(4-(Dimethylamino)benzylidene)-1*H*-indene-1,3(2*H*)-dione (**6h**)

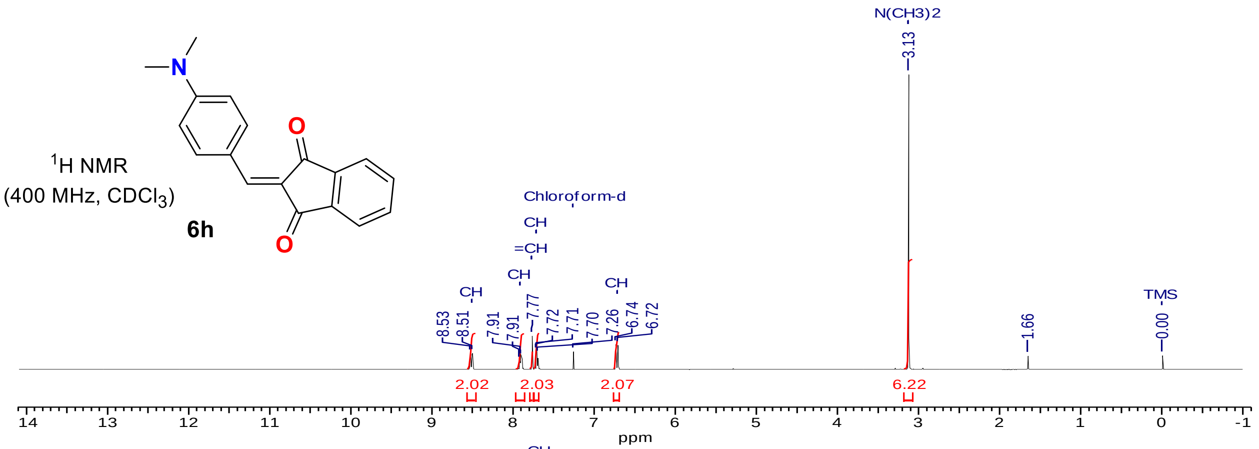

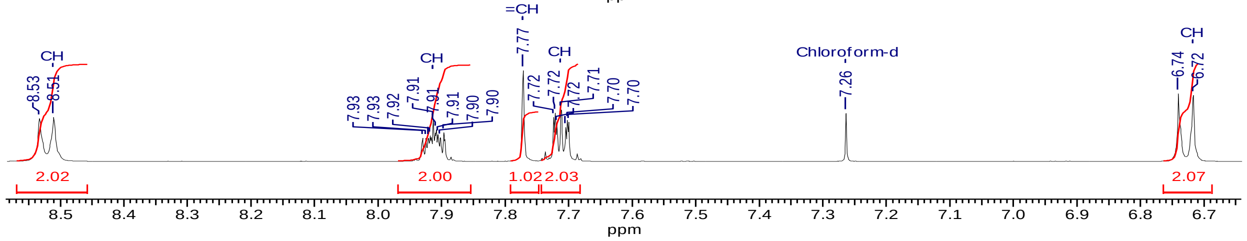

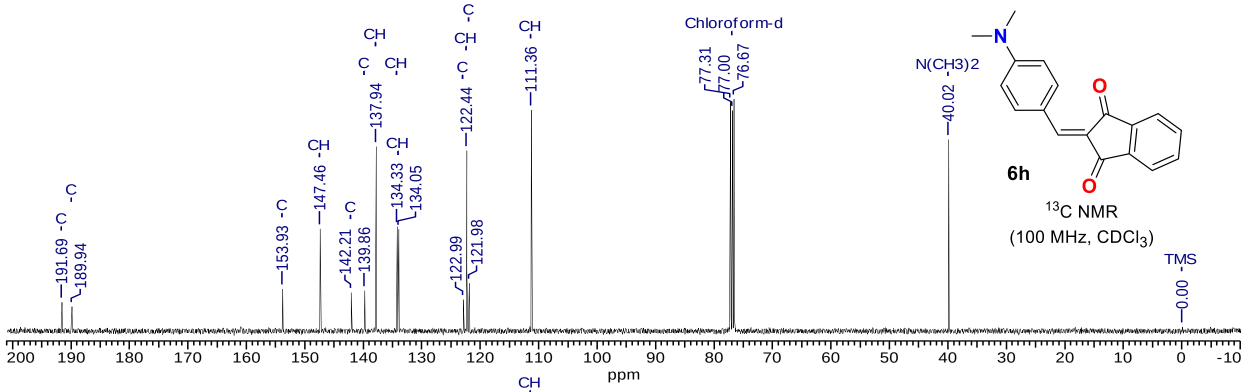

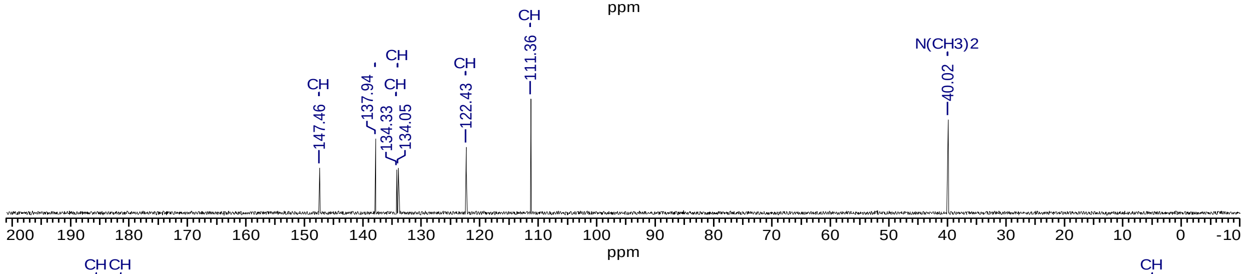

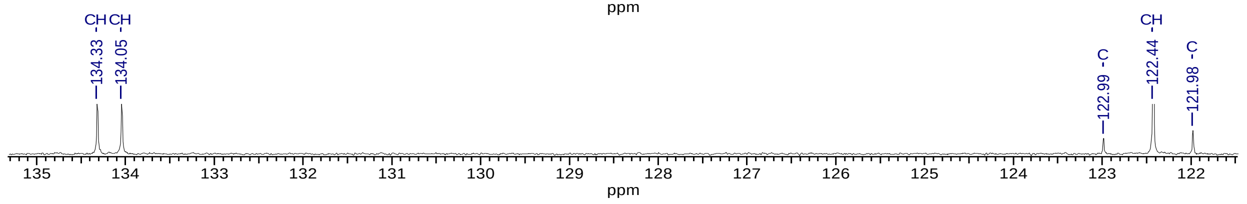

^{1}H and $^{13}C\{^{1}H\}$ NMR spectra of 2-(3-Methyl-1,4-diphenylindeno[1,2-*b*]pyrazolo[4,3-*e*]pyridin-5(1*H*)-ylidene)malononitrile (**7a**)

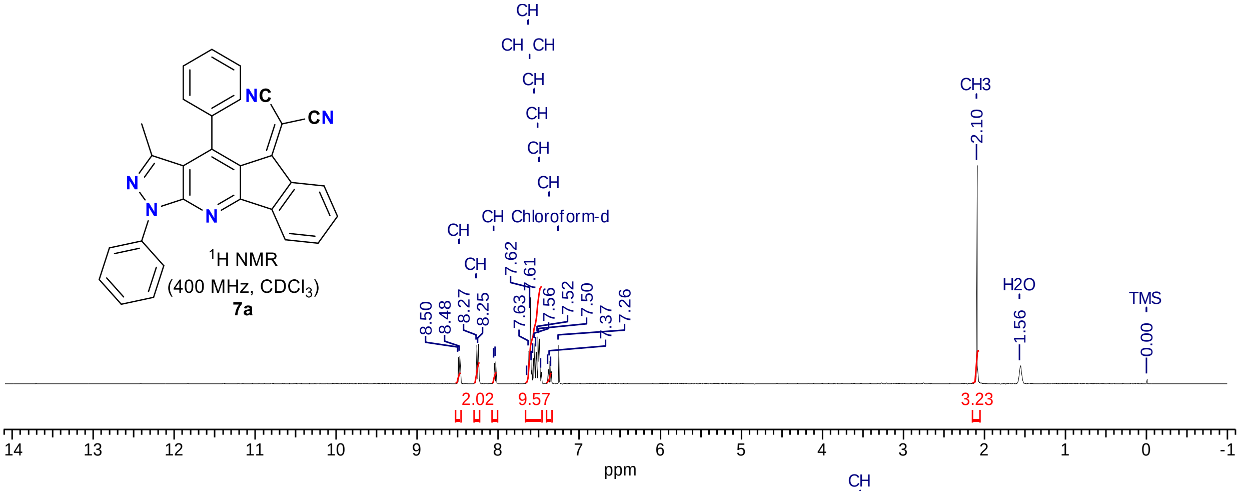

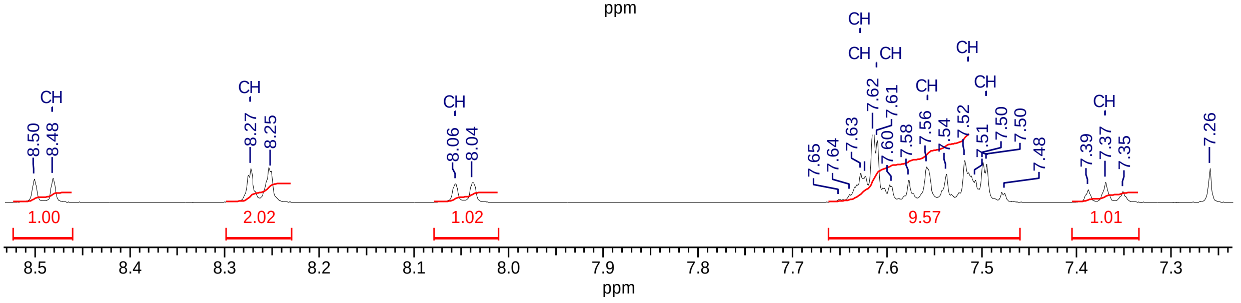

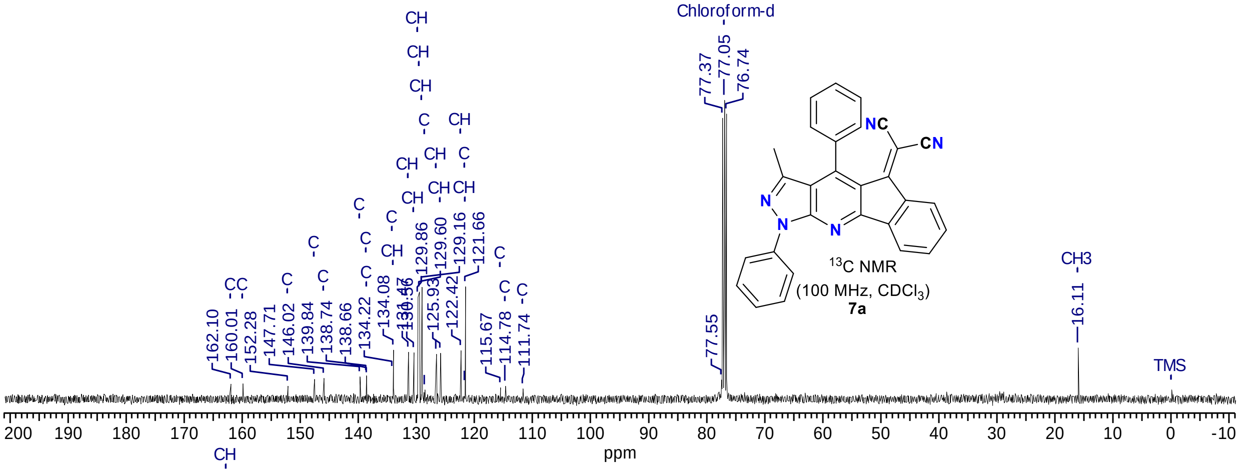

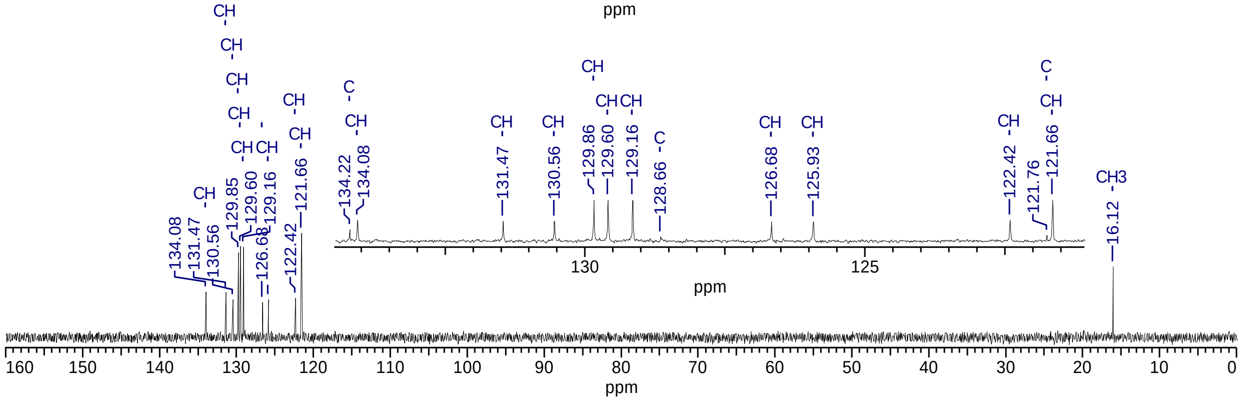

^{1}H and ^{13}C{^{1}H} NMR spectra of 2-(4-(4-methoxyphenyl)-3-methyl-1-phenylindeno[1,2-*b*]pyrazolo[4,3-*e*]pyridin-5(1*H*)-ylidene)malononitrile (**7b**)

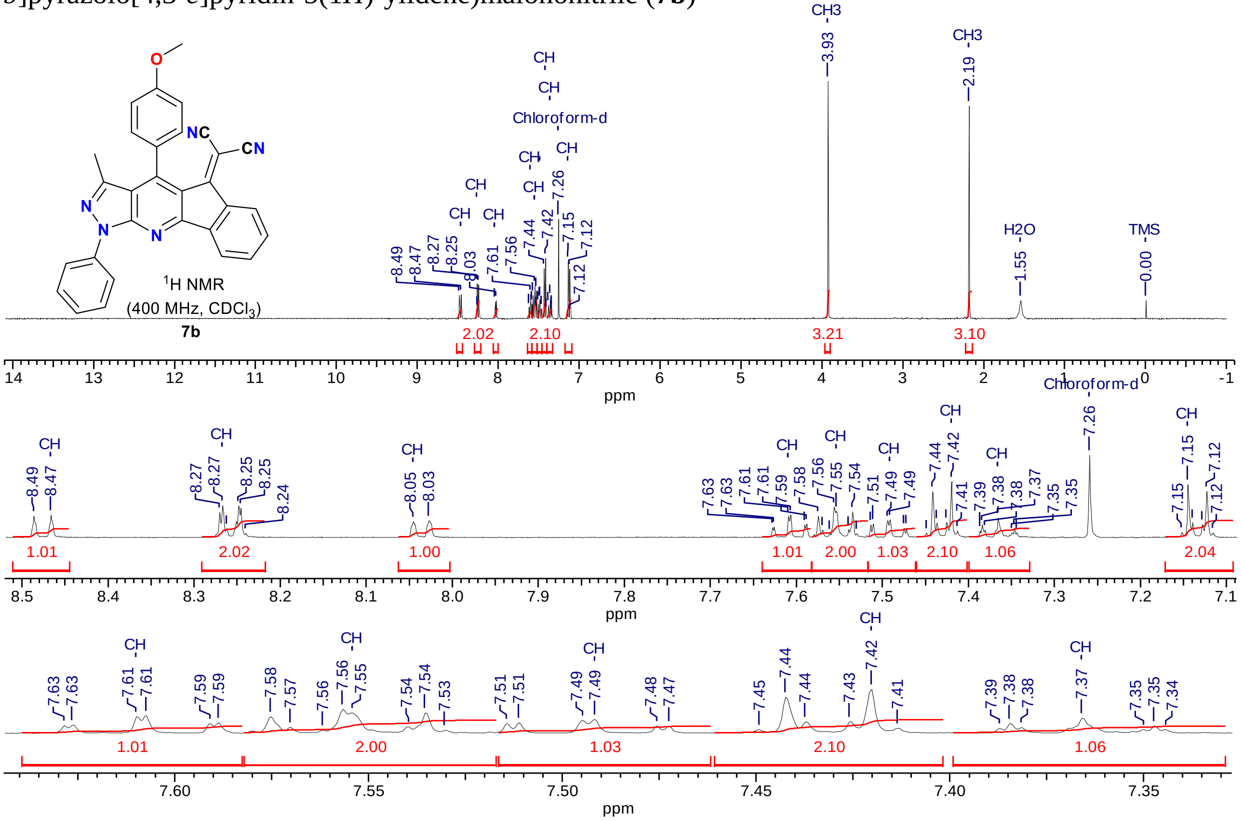

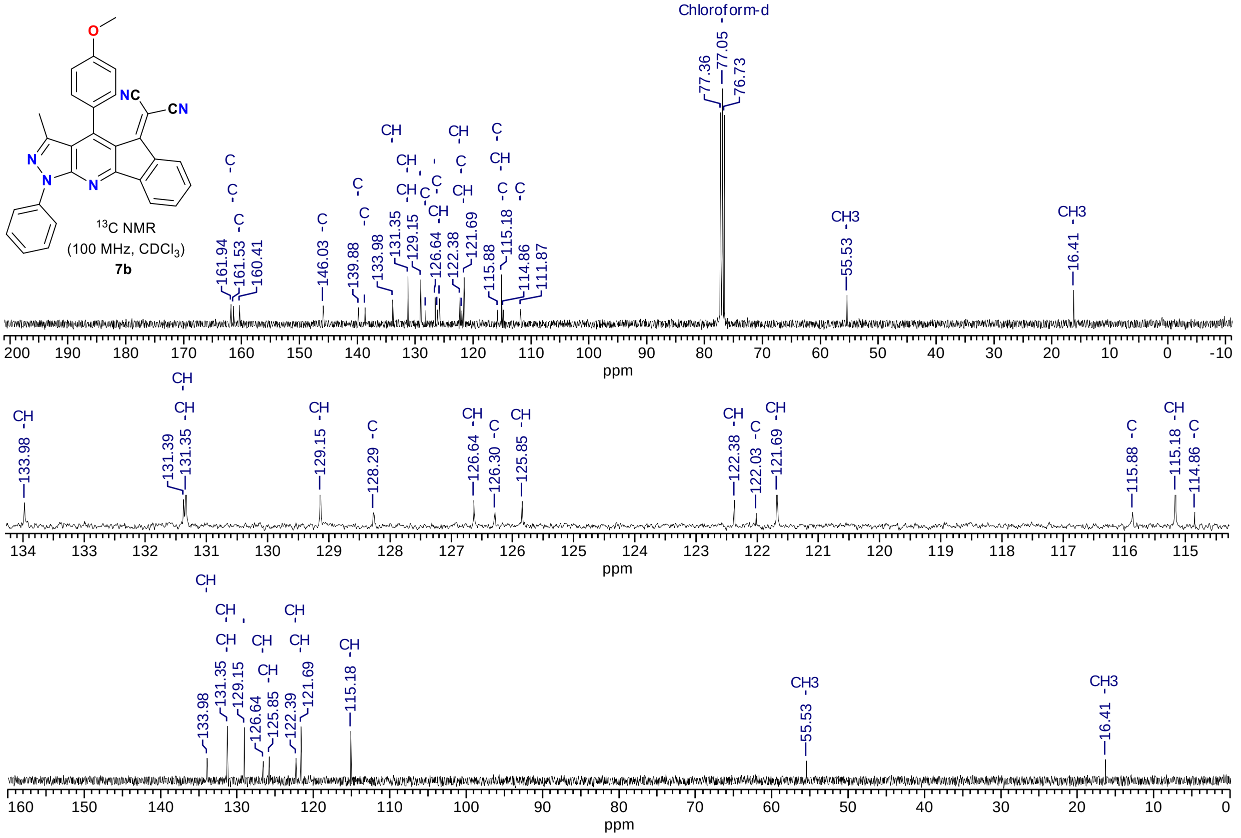

^{1}H and ^{13}C{^{1}H} NMR spectra of 2-(3-Methyl-1-phenyl-4-(4-(trifluoromethyl)phenyl)indeno[1,2-*b*]pyrazolo[4,3-*e*]pyridin-5(1*H*)-ylidene)malononitrile (**7c**)

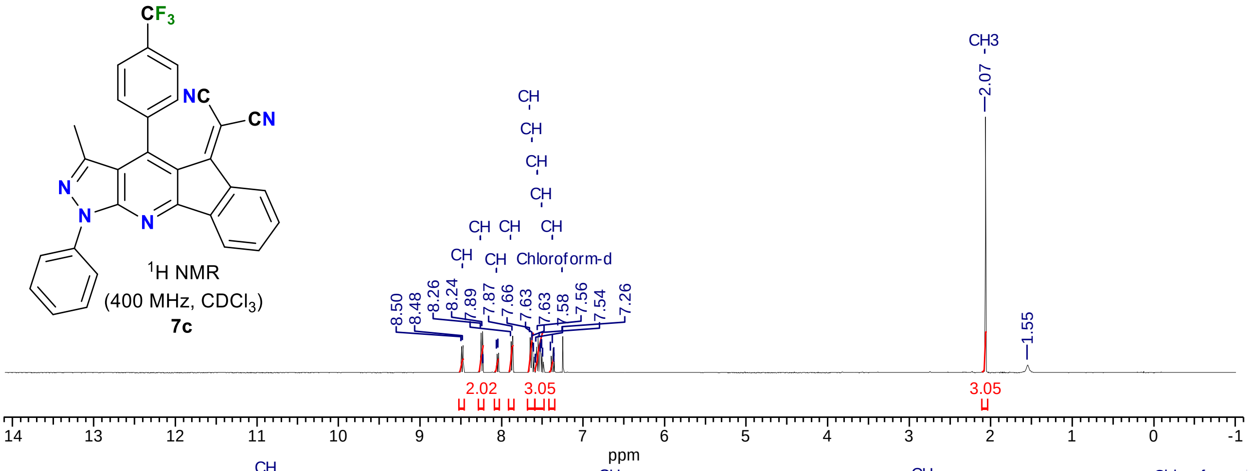

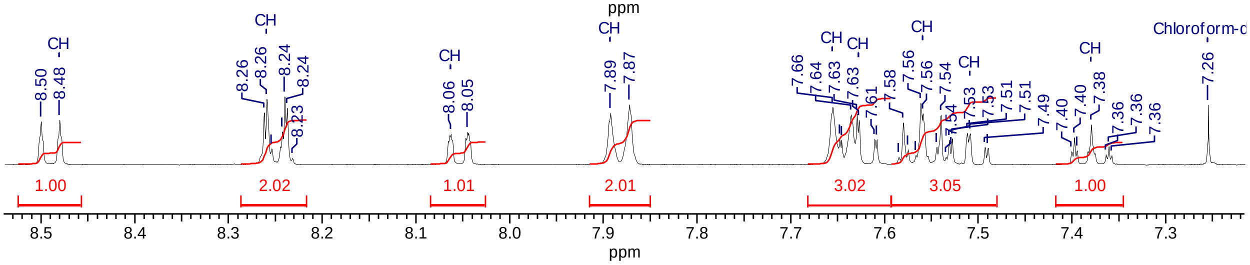

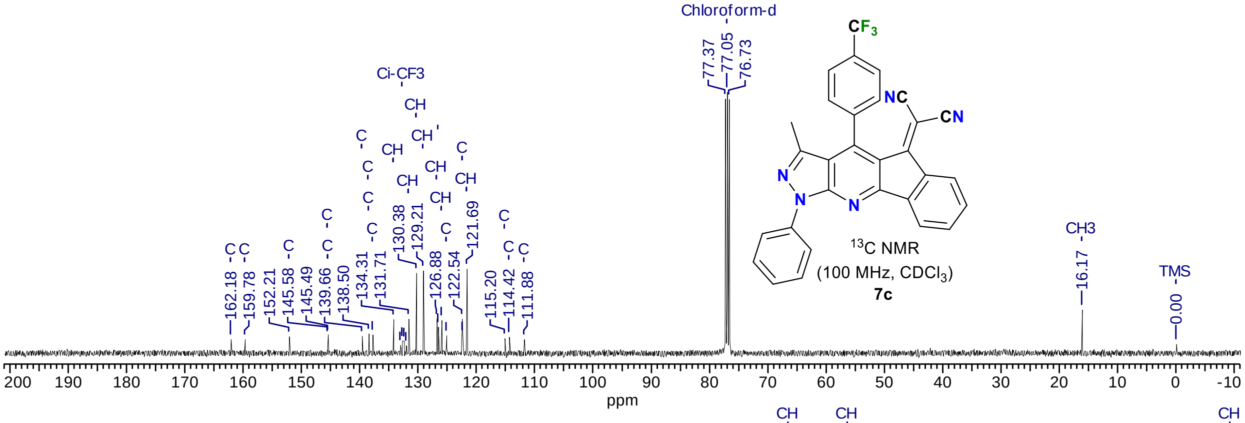

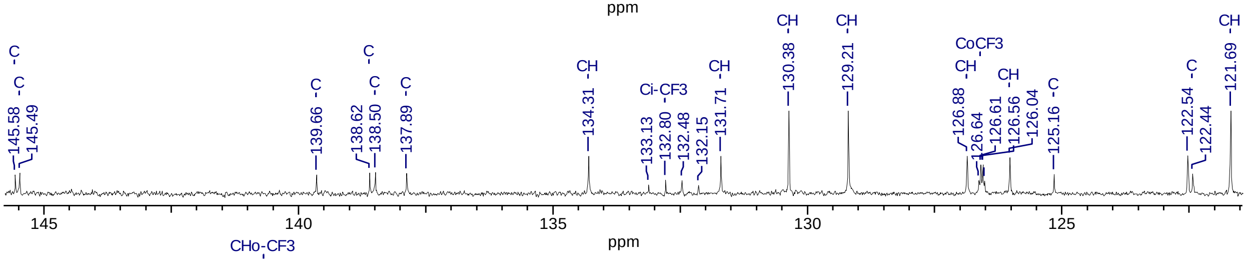

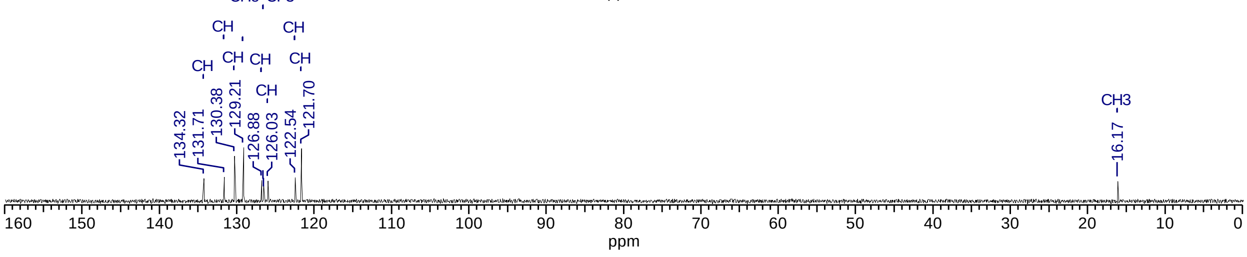

^{1}H and ^{13}C{^{1}H} NMR spectra of 2-(4-(4-(Dimethylamino)phenyl)-3-methyl-1-phenylindeno[1,2-*b*]pyrazolo[4,3-*e*]pyridin-5(1*H*)-ylidene)malononitrile (**7d**)

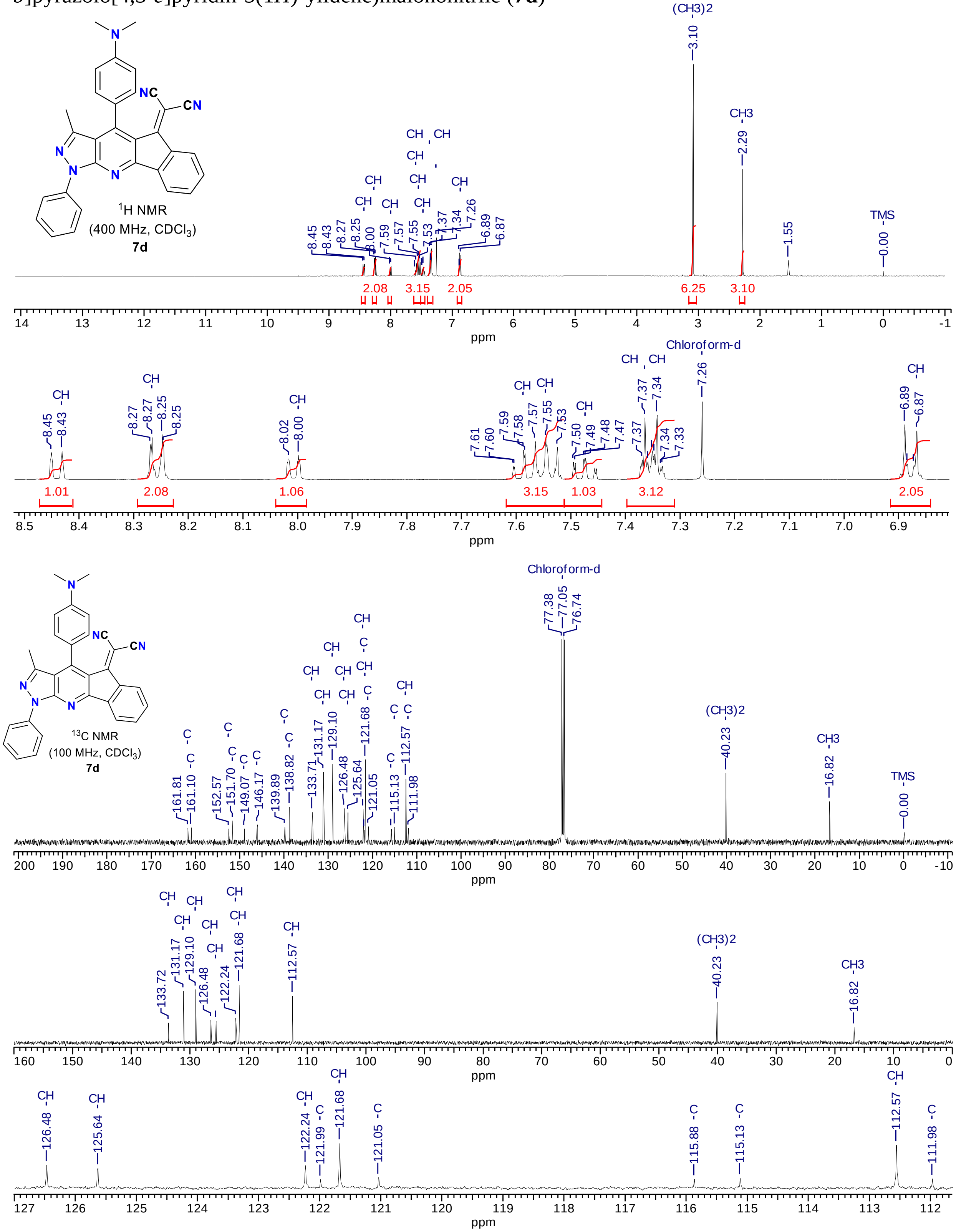

8. ORTEP Drawing for Structure 7b

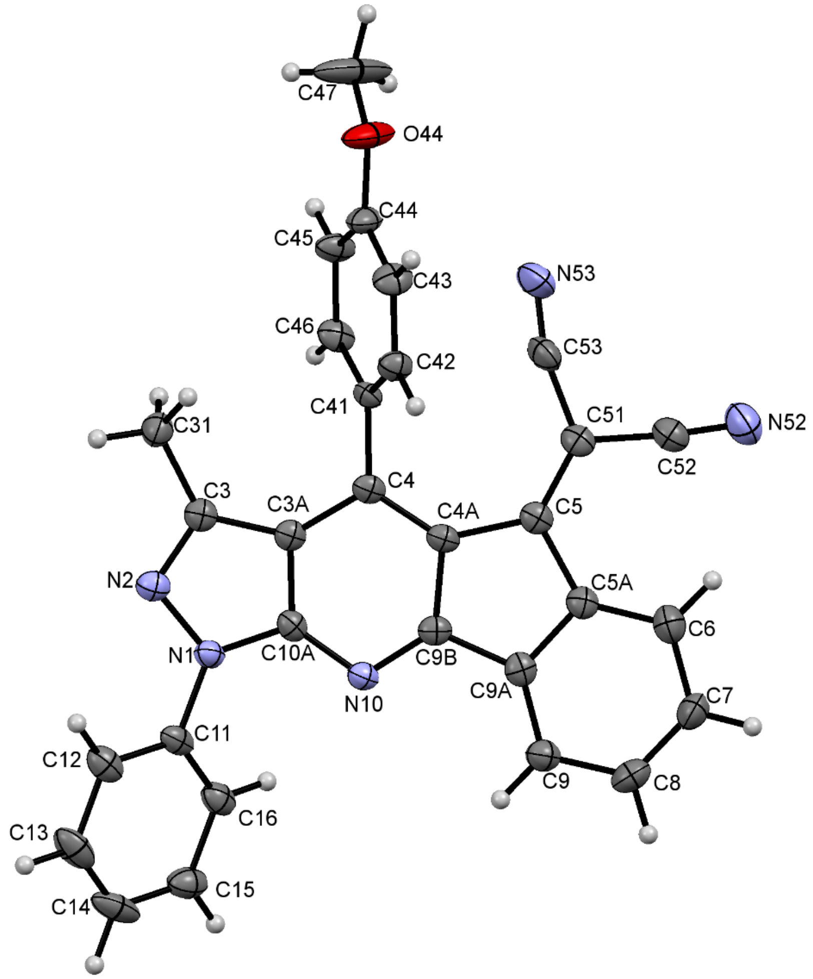

Fig. S12. ORTEP drawing for structure **7b**. Displacement ellipsoids are drawn at the 70% probability level and hydrogen atoms are shown as small spheres of arbitrary radius.

9. HRMS analysis data of the final products 7a-d

Qualitative Compound Report

**Data File**	JOH-CR-MEZCLA.d	**Sample Name**	Unavailable
**Sample Type**	Unavailable	**Position**	Unavailable
**Instrument Name**	Unavailable	**User Name**	Unavailable
**Acq Method**		**Acquired Time**	Unavailable
**IRM Calibration Status**	Success	**DA Method**	generalESI+.m
**Comment**	Sample information is unavailable		

Compound Table

Compound Label	RT	Mass	Abund	Formula	Tgt Mass	Diff (ppm)	MFG Formula	DB Formula
Cpd 1: C29 H17 N5	7.535	435.1477	5796	C29 H17 N5	435.1484	-1.6	C29 H17 N5	C29 H17 N5

Compound Label	*m/z*	RT	Algorithm	Mass
Cpd 1: C29 H17 N5	436.1548	7.535	Find By Formula	435.1477

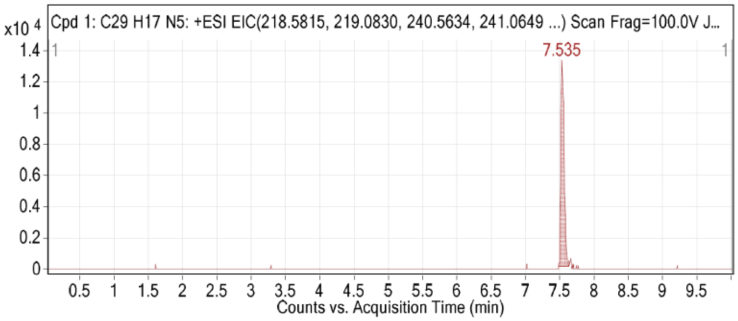

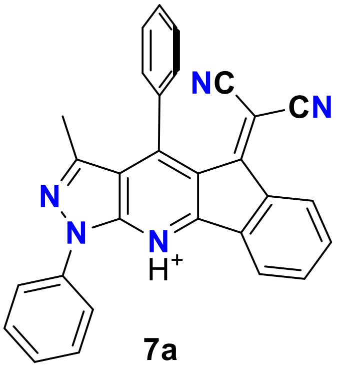

7a

[M+H$^+$] $C_{29}H_{18}N_5^+$
Calc. for 436.1557

MS Spectrum

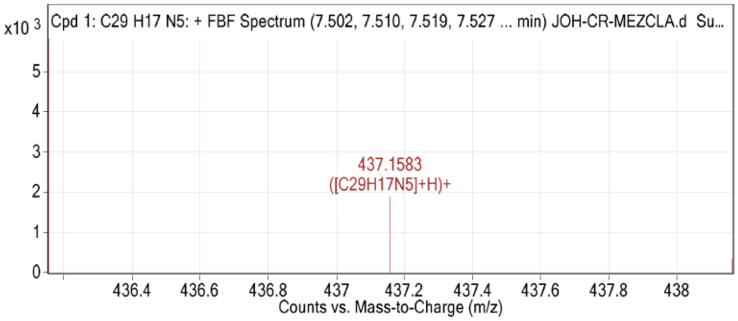

MS Zoomed Spectrum

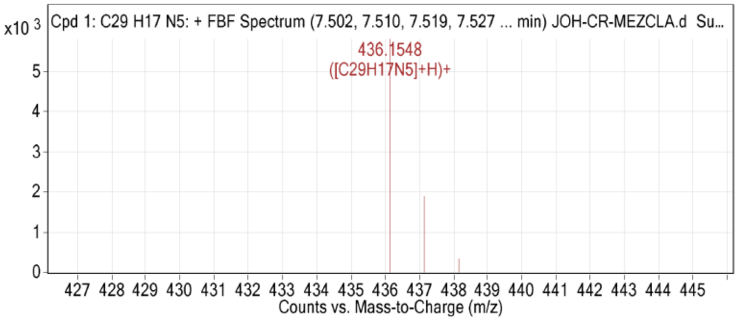

MS Spectrum Peak List

m/z	z	Abund	Formula	Ion
436.1548	1	5795.55	C29H17N5	(M+H)+
437.1583	1	1895.17	C29H17N5	(M+H)+

Qualitative Compound Report

**Data File**	JOH-CR-MEZCLA.d	**Sample Name**	Unavailable
**Sample Type**	Unavailable	**Position**	Unavailable
**Instrument Name**	Unavailable	**User Name**	Unavailable
**Acq Method**		**Acquired Time**	Unavailable
**IRM Calibration Status**	Success	**DA Method**	generalESI+.m
**Comment**	Sample information is unavailable		

Compound Table

Compound Label	RT	Mass	Abund	Formula	Tgt Mass	Diff (ppm)	MFG Formula	DB Formula
Cpd 2: C30 H19 N5 O	7.494	465.1579	2019	C30 H19 N5 O	465.159	-2.34	C30 H19 N5 O	C30 H19 N5 O

Compound Label	*m/z*	RT	Algorithm	Mass
Cpd 2: C30 H19 N5 O	466.1647	7.494	Find By Formula	465.1579

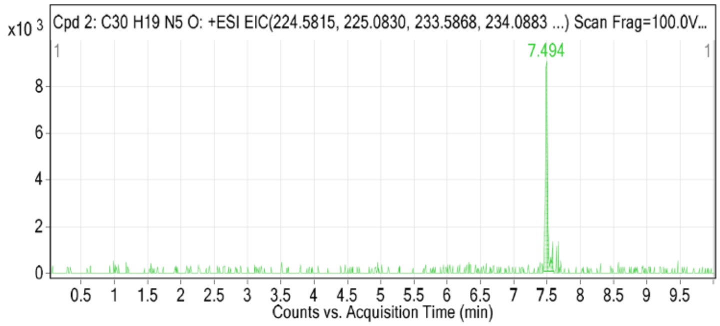

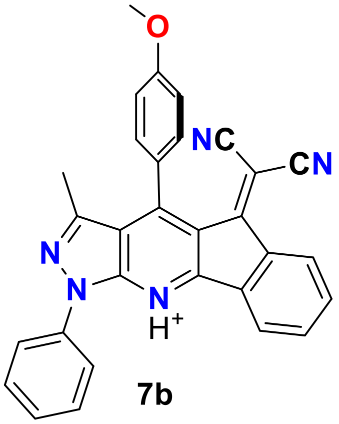

7b

[M+H$^+$] $C_{30}H_{20}N_5O^+$
Calc. for 466.1662

MS Spectrum

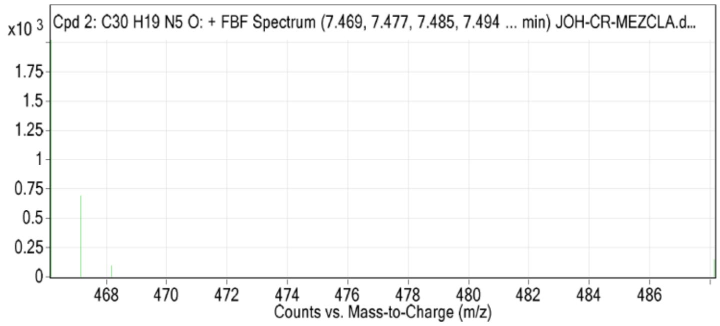

MS Zoomed Spectrum

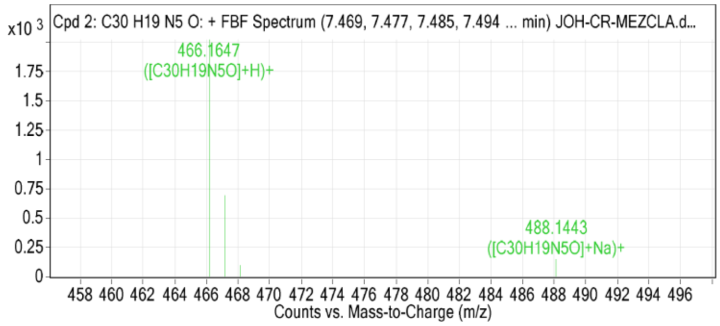

MS Spectrum Peak List

m/z	z	Abund	Formula	Ion
466.1647	1	2018.9	C30H19N5O	(M+H)+
467.1694	1	686.01	C30H19N5O	(M+H)+

Qualitative Compound Report

**Data File**	JOH-CR-MEZCLA.d	**Sample Name**	Unavailable
**Sample Type**	Unavailable	**Position**	Unavailable
**Instrument Name**	Unavailable	**User Name**	Unavailable
**Acq Method**		**Acquired Time**	Unavailable
**IRM Calibration Status**	Success	**DA Method**	generalESI+.m
**Comment**	Sample information is unavailable		

Compound Table

Compound Label	RT	Mass	Abund	Formula	Tgt Mass	Diff (ppm)	MFG Formula	DB Formula
Cpd 5: C30 H16 F3 N5	7.627	503.1348	3360	C30 H16 F3 N5	503.1358	-1.95	C30 H16 F3 N5	C30 H16 F3 N5

Compound Label	*m/z*	RT	Algorithm	Mass
Cpd 5: C30 H16 F3 N5	504.1419	7.627	Find By Formula	503.1348

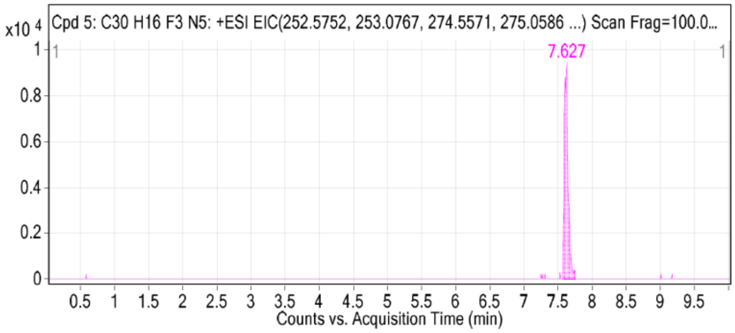

MS Spectrum

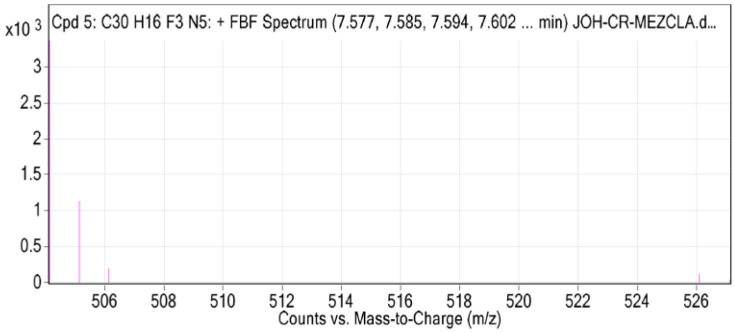

MS Zoomed Spectrum

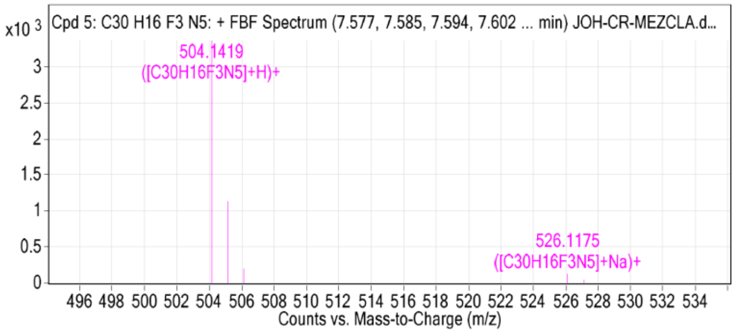

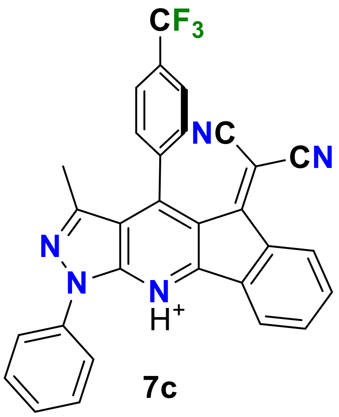

7c

$[M+H^+]$ $C_{30}H_{27}F_3N_5^+$
Calc. for 504.1431

MS Spectrum Peak List

m/z	z	Abund	Formula	Ion
504.1419	1	3360.43	C30H16F3N5	(M+H)+
505.1464	1	1131.82	C30H16F3N5	(M+H)+

Qualitative Compound Report

**Data File**	JOH-CR-MEZCLA.d	**Sample Name**	Unavailable
**Sample Type**	Unavailable	**Position**	Unavailable
**Instrument Name**	Unavailable	**User Name**	Unavailable
**Acq Method**		**Acquired Time**	Unavailable
**IRM Calibration Status**	Success	**DA Method**	generalESI+.m
**Comment**	Sample information is unavailable		

Compound Table

Compound Label	RT	Mass	Abund	Formula	Tgt Mass	Diff (ppm)	MFG Formula	DB Formula
Cpd 4: C31 H22 N6	7.761	478.1895	18643	C31 H22 N6	478.1906	-2.22	C31 H22 N6	C31 H22 N6

Compound Label	*m/z*	RT	Algorithm	Mass
Cpd 4: C31 H22 N6	479.1969	7.761	Find By Formula	478.1895

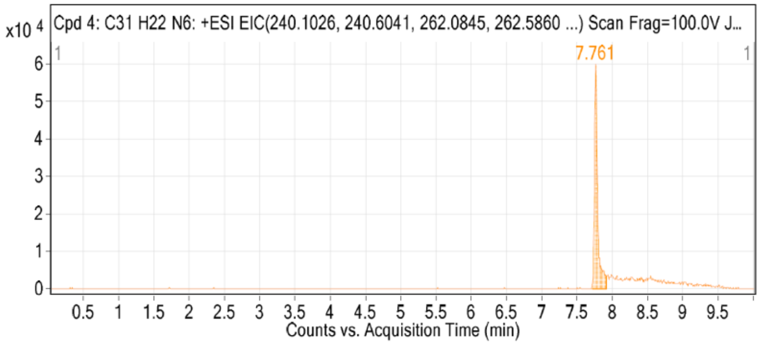

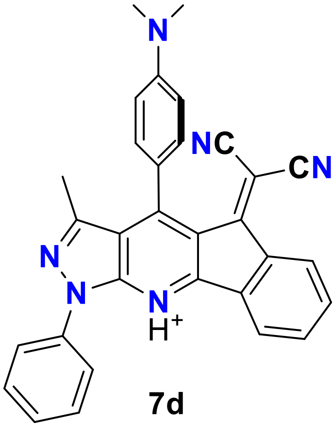

7d

[M+H$^+$] $C_{31}H_{23}N_6^+$

Calc. for 479.1979

MS Spectrum

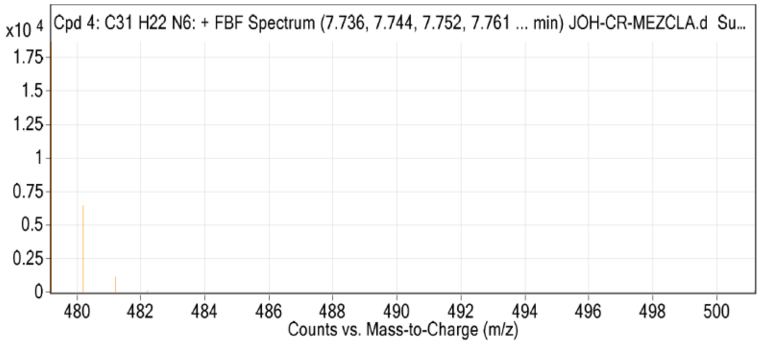

MS Zoomed Spectrum

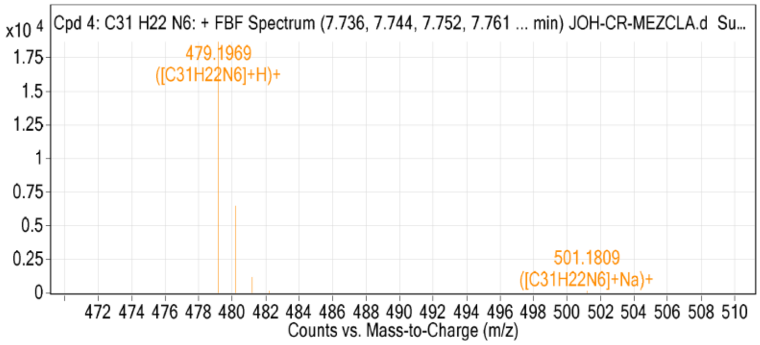

MS Spectrum Peak List

m/z	z	Abund	Formula	Ion
479.1969	1	18642.89	C31H22N6	(M+H)+
480.1996	1	6443.86	C31H22N6	(M+H)+

10. References

1 L. Palatinus and G. Chapuis, *J. Appl. Cryst.*, 2007, **40**, 786-790

2 G. M. Sheldrick, *Acta Cryst.*, 2015, **C71**, 3-8.

3 C. F. Macrae, I. J. Bruno, J. A. Chisholm, P. R. Edgington, P. McCabe, E. Pidcock, L. Rodriguez-Monge, R. Taylor, J. van de Streek and P. A. Wood, *J. Appl. Cryst.*, 2008, **41**, 466-470.

4 J. Quiroga, D. Cobo, B. Insuasty, R. Abonía, S. Cruz, M. Nogueras and J. Cobo, *J. Heterocycl. Chem.*, 2008, **45**, 155–159.

5 J. Portilla, C. Lizarazo, J. Cobo and C. Glidewell, *Acta Cryst.*, 2011, **C67**, 479–483.

6 C. Würth, M. Grabolle, J. Pauli, M. Spieles and U. Resch-Genger, *Nat. Protoc.*, 2013, **8**, 1535–1550.

7 U. Resch-Genger and K. Rurack, *Pure Appl. Chem.*, 2013, **85**, 2005–2026.

8 G. A. Crosby and J. N. Demas, *J. Phys. Chem.*, 1971, **75**, 991–1024.

9 M. D. Hanwell, D. E. Curtis, D. C. Lonie, T. Vandermeersch, E. Zurek and G. R. Hutchison, *J. Cheminform.*, 2012, **4**, 1–17.

10 M. J. Frisch, G. W. Trucks, H. B. Schlegel, G. E. Scuseria, M. A. Robb, J. R. Cheeseman, G. Scalmani, V. Barone, G. A. Petersson, H. Nakatsuji, X. Li, M. Caricato, A. Marenich, J. Bloino, B. G. Janesko, R. Gomperts, B. Mennucci, H. P. Hratchian, J. V. Ortiz, A. F. Izmaylov, J. L. Sonnenberg, D. Williams-Young, F. Ding, F. Lipparini, F. Egidi, J. Goings, B. Peng, A. Petrone, T. Henderson, D. Ranasinghe, V. G. Zakrzewski, J. Gao, N. Rega, G. Zheng, W. Liang, M. Hada, M. Ehara, K. Toyota, R. Fukuda, J. Hasegawa, M. Ishida, T. Nakajima, Y. Honda, O. Kitao, H. Nakai, T. Vreven, K. Throssell, J. A. Montgomery, Jr., J. E. Peralta, F. Ogliaro, M. Bearpark, J. J. Heyd, E. Brothers, K. N. Kudin, V. N. Staroverov, T. Keith, R. Kobayashi, J. Normand, K. Raghavachari, A. Rendell, J. C. Burant, S. S. Iyengar, J. Tomasi, M. Cossi, J. M. Millam, M. Klene, C. Adamo, R. Cammi, J. W. Ochterski, R. L. Martin, K. Morokuma, O. Farkas, J. B. Foresman and D. J. Fox, Gaussian 09, Revision A.1. Wallingford CT, 2009.
